# Mothers’ neural response to valenced infant interactions predicts postpartum depression and anxiety

**DOI:** 10.1371/journal.pone.0250487

**Published:** 2021-04-27

**Authors:** Megan Kate Finnegan, Stephanie Kane, Wendy Heller, Heidemarie Laurent

**Affiliations:** 1 Psychology Department, University of Illinois at Urbana-Champaign, Champaign, IL, United States of America; 2 Neuroscience Program, University of Illinois at Urbana-Champaign, Urbana, IL, United States of America; 3 Department of Psychological Science, University of Arkansas, Fayetteville, AR, United States of America; Icahn School of Medicine at Mount Sinai Hospital, UNITED STATES

## Abstract

It is currently unknown whether differences in neural responsiveness to infant cues observed in postpartum affective disturbance are specific to depression/anxiety or are better attributed to a common component of internalizing distress. It is also unknown whether differences in mothers’ brain response can be accounted for by effects of past episodes, or if current neural processing of her child may serve as a risk factor for development of future symptoms. Twenty-four mothers from a community-based sample participated in an fMRI session viewing their 3-month- old infant during tasks evoking positive or negative emotion. They were tracked across the ensuing 15 months to monitor changes in affective symptoms. Past and current episodes of depression and anxiety, as well as future symptoms, were used to predict differences in mothers’ hemodynamic response to their infant in positive compared to negative emotion contexts. Lower relative activation in largely overlapping brain regions involving frontal lobe structures to own infant positive vs. negative emotion was associated with concurrent (3-month) depression diagnosis and prospective (3–18 month) depression and anxiety symptoms. There was little evidence for impacts of past psychopathology (more limited effect of past anxiety and nonsignificant effect of past depression). Results suggest biased maternal processing of infant emotions during postpartum depression and anxiety is largely accounted for by a shared source of variance (internalizing distress). Furthermore, differential maternal responsiveness to her infant’s emotional cues is specifically associated with the perpetuation of postpartum symptoms, as opposed to more general phenotypic or scarring effects of past psychopathology.

## 1. Introduction

The transition to motherhood represents a vulnerable time marked by increasing prevalence of affective symptomatology [1–3]. Though demographic and contextual risk factors for peripartum depression are known, the proximal processes driving symptoms remain unclear. In particular, how current responding to affective cues in the social environment relates to future depression or anxiety remains largely unexplored. This study seeks to understand how differences in mothers’ neural responsiveness to their infants relate to postpartum affective symptoms, and whether such differences represent (a) effects of past psychopathology versus propensity to symptoms going forward, and (b) effects specific to depression or anxiety versus a common internalizing distress component that gives rise to both of these syndromes.

Affective neuroscience has largely based conclusions about brain bases of psychopathology on responses to stimuli with standardization and clearly defined valence (*i*.*e*., [4–6]), yet whose ecological validity is limited. While these may provide insight into the fundamental mechanisms underlying emotion, the context of personally meaningful images and sounds can elucidate how psychological distress manifests in ways relevant for daily function. That is, to understand how a new mother navigates more positively or negatively valenced situations and how that relates to affective psychopathology, it makes sense to incorporate stimuli closer to her everyday experience. Guided by this consideration, the present study examines neural response to salient affective stimuli in the mother’s social environment—*i*.*e*., her own infant in emotion-eliciting situations. Acknowledging that such neural responses both feed into and result from affective symptoms, we investigate both potential phenotypic/scarring effects of past psychopathology and prospective relations between early postpartum brain function and future symptoms.

Previous work on normative maternal brain response has identified several regions exhibiting preferential response to a mother’s own child across emotional contexts. A meta-analysis by Rigo and colleagues [7] revealed preferential activity for their own compared to other infants subcortically in the basal ganglia, thalamus, and amygdala; and cortically in the insula and inferior frontal gyrus (IFG). These were predominately left lateralized, which the authors suggested reflects the socially rewarding experience of one’s own child. Indeed, reward-inducing positive emotions may be a critical component of innate mammalian parenting behaviors [8]. These same regions show reduced activity during reward processing when individuals are depressed [9], suggesting a potential biological mechanism for the caregiving behavioral deficits observed with peripartum depression (such as impaired sensitivity to their infant’s emotional cues; [10]). A systematic review of neurofunctional changes of postpartum depression observed substantial overlap with major depressive disorder (MDD) [11], strengthening the idea that depression may stem from and/or perpetuate a disruption in the rewarding aspects of mother-child interactions. This could manifest as blunted processing of her infant’s cues in emotionally positive relative to negative contexts, a possibility supported by previous findings of reduced activation to own infant joy relative to distress faces associated with concurrent depressive symptoms [12]. However, the cross-sectional design of that study cannot answer whether biased processing of infant emotions prospectively predicts postpartum psychopathology. It is also unclear how altered neural response may relate to history of affective disturbance. Behaviorally, previous episodes of affective disorders have been identified as an important predictor of postpartum depression [13] and anxiety [14], and there is evidence for both overlapping and distinct associations between past/current/future depression and affect regulation strategies [15]. However, research disentangling contributions of past vs. current psychopathology to maternal brain response is lacking.

Another important question is whether brain differences attributed to postpartum depression are syndrome-specific or represent a general internalizing distress shared with anxiety. In fact, comorbidity with anxiety appears to be more frequent than unipolar depression alone [16]. The small body of research investigating peripartum anxiety has yielded fragmentary evidence, with one study showing no state anxiety-related difference in brain response to their own compared to other infants’ negative emotions [17], and another study finding reduced amygdala response to own and unfamiliar infant positive emotion with increasing trait anxiety only among mothers who were not depressed [18]. Underlining the potential overlap [19], documented an association between IFG and insula activation to negative emotion faces and both anxiety and depression symptoms at different postpartum times. These findings are consistent with previous anxiety research in non-peripartum populations showing differences in processing valenced stimuli in some of the same regions implicated in both normative maternal response and depression such as the insula, prefrontal cortex, anterior cingulate, and amygdala [20–22]. However, it remains unclear how maternal brain response to own infant across differing emotion contexts may uniquely contribute to anxiety in the postpartum.

The current study aims to elucidate how maternal brain responses to their infants’ socio-emotional cues relate to past, current, and future depression and anxiety. We address the following questions: **(1)** How does maternal neural response to their infant in positive vs. negative emotion situations relate to depression and/or anxiety symptoms in the ensuing 15 months? **(2)** Are there unique effects associated with current vs. past depression and/or anxiety diagnoses? and **(3)** Are there unique effects associated with postpartum depression vs. anxiety symptoms? Based on the available literature, we predicted relatively greater neural activation to positive compared to negative infant emotion situations would predict lower levels of postpartum affective symptoms. Lacking prior work probing temporal (past/current) and syndromal (depression/anxiety) specificity in this population, the latter questions were approached in a more exploratory manner. These questions were tested in a community sample of mothers assessed longitudinally. This dataset was originally described in [23] where it was used to explore the association of dispositional mindfulness with preferential neural activity to infant affect. Maternal brain response to videos of their infant during arm-restraint and peek-a-boo interactions was measured at the initial (3-month) assessment, as were past and current affective disorder diagnoses. Self-reported symptoms at each assessment provided an estimate of overall depression and anxiety symptom severity across 3–18 months postpartum.

## 2. Methods and materials

### 2.1. Participants and procedures

Results reported here are based on a subset of women from a larger study examining mother-infant stress regulation. Women were recruited from community agencies serving low-income women in the Pacific Northwest. Twenty-five mothers agreed to participate in the optional neuroimaging component, with 24 included in the final analysis (see Table 1). One mother was dropped from the analysis due to excessive head movement. Mothers were included if they could speak English, had an infant younger than 12 weeks-old, did not present with psychotic symptoms on clinical interview, and planned to remain in the region until the infant was 18 months old. Inclusion in the neuroimaging portion was based on absence of MRI contraindications; all women meeting criteria (See S1 File for more information on changes made in the initial recruitment strategies) were offered participation. Mothers were scanned at the initial 3-month postpartum assessment (T1), and returned for assessments at 6, 12, and 18-months postpartum (T2, T3, and T4). Of the 25 original mothers, all returned for the 6-month assessment, 19 returned at 12-months and 16 returned at 18-months. This dataset is also described in [23] and [24]. None of the mothers reported using psychotropic medication either during pregnancy or at the time of the scan (T1 assessment). One mother reported taking antidepressant medication at T2 only. Mothers who completed all assessments did not significantly differ from those who dropped out in demographic or psychological measures tested (See S1 File). The study was approved by the University of Oregon Institutional Review Board, and participants gave written informed consent.

**Table 1:** Demographic information for mothers included in final analysis.

**Demographic**		**Mean (SD) [Min, Max]**	
*Age*		26.7 years (3.9) [19, 33]	
		**Median [Min, Max]**	
*Annual Income*		$20,000 - $30,00 [Under $5,000, Between $75k –$100k]	
	**Category**		**Frequency *(n = 24)***
*Primary Racial Identification*	Caucasian		18
	Latina		3
	Asian American		1
	Other		2
*Education Completed*	High School or equivalent		4
	Vocational School		1
	Some College		13
	4-year College Degree		1
	Master’s Degree		3
	Other		2
*Partner Status*	Single		1
	Dating		1
	Living with Someone		8
	Married		12
	Separated		1
	Domestic Partnership		1
*Mode of Delivery*	Vaginal		13
	Cesarean		9
Breastfeeding*	Yes		21
*Child Birth Order*	First		13
	Second		9
	Third		2

*Taken at time of first assessment (3-month postpartum).

### 2.2. Psychological data

#### 2.2.1. Postpartum symptoms

Participants completed the Center for Epidemiologic Studies Depression Scale (CESD) [25] and the Beck Anxiety Inventory (BAI) [26] at every assessment. One participant did not complete the T3, but returned for the T4 assessment (See *S1 File* for demographic comparisons). The CESD captures general symptoms of depression with an emphasis on affective components [25], and the BAI measures both somatic and cognitive symptoms of anxiety [27]. Hierarchical linear modeling was used to estimate mothers’ postpartum symptom trajectories in the presence of missing data (See Table 2).

**Table 2:** Psychopathology data for mothers included in final analysis.

**Measure**		**Mean (SD) [Min, Max]**
*CESD-T1raw*		8.38 (9.54) [0, 43]
*BAI-T1raw*		7.56 (7.23) [0, 28]
*CESD-T4raw*		11.00 (8.38) [1, 29]
*BAI-T4raw*		9.00 (8.82) [1, 30]
*CESD-T1-T4*		10.36 (5.29) [2.92, 28.85]
*BAI-T1-T4*		8.93 (6.90) [1.43, 30.01]
	**Category**	**Frequency *(n = 24)***
*SCID*	Dep Hx	4
	Anx Hx	2
	Dep Dx	2
	Anx Dx	4

**Note.** *CESD-T1raw and CESD-T4raw are observed CESD scores for the 3-month and 18-month assessments*. *Similarly*, *the BAI-T1raw and BAI-T4raw are observed BAI scores*. *CESD[T1-4] = estimated mean CESD score across all assessments time point; BAI[T1-4] = estimated mean BAI score; Dep Hx = history of depressive disorder based on the Structured Clinical Interview for DSM-IV(SCID); Dep Dx = current depressive disorder; Anx Hx = anxiety disorder history; Anx Dx = current anxiety disorder*

#### 2.2.2. Past/current diagnoses

The presence of past (prior to T1) and current (at T1) depression and anxiety disorders were coded using the research version of the Structured Clinical Interview for DSM-IV (SCID) [28]. Six met criteria for major depressive disorder (2 current, 4 past) and six met criteria for an anxiety disorder (4 current, 2 past). Although exact age of onset for past depression or anxiety was not gathered, all mothers meeting criteria for past depressive episodes and/or anxiety disorders reported their worst episode occurring prior to pregnancy. Depression history (Dep-Hx), depression current diagnosis (Dep-Dx), anxiety history (Anx-Hx), and anxiety current diagnosis (Anx-Dx) were used as covariates in subsequent regression analyses.

### 2.3. fMRI task

Participants engaged in two separate block-designed paradigms repeated for two runs each. The first involved viewing infants during emotion-eliciting tasks, and the second (not reported here) involved viewing and/or labeling emotionally expressive faces.

Before scanning, mothers were guided in arm restraint (designed to elicit negative emotion; [29]) and peekaboo (designed to elicit positive emotion; [30]) interactions during a video recorded home visit. Recordings were framed to center the infant’s face and zoomed to show no body parts below the neck. Videos contained both audio and visual streams. The 15s segments displaying maximal positive (peekaboo) and negative (arm restraint) emotions were selected for presentation through reference to the Baby FACS [31] emotional expression coding framework.

**Fig 1:**
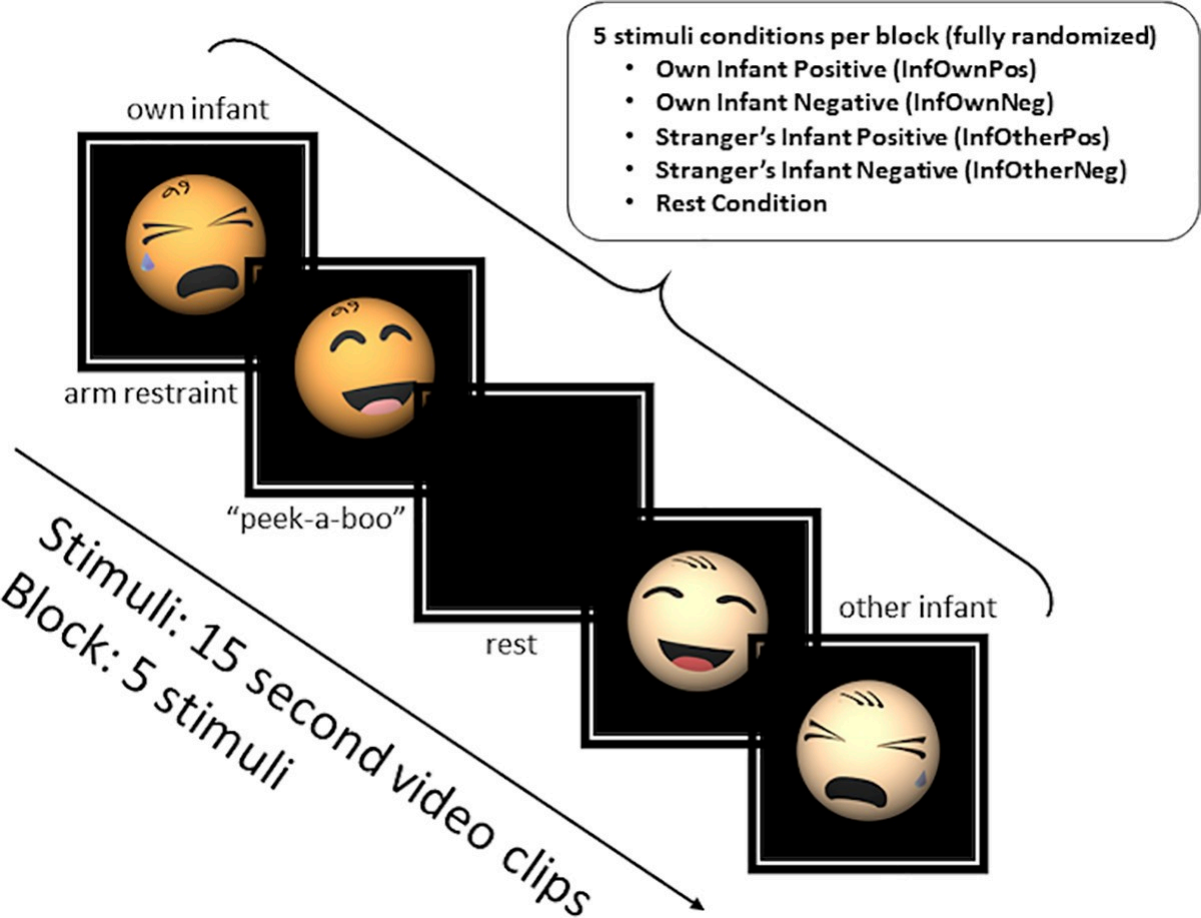
Overview of infant viewing task. Note that the mother’s infant and a stranger’s age-matched infant were used in each of the videos represented in this diagram as infant caricatures.

Mothers were verbally instructed to “watch and respond as you would naturally” to videos of their own or unknown infants. The unknown infant (a pilot drawn from the same overall study sample) displayed to all participants was matched for age and completed the same arm-restraint and peekaboo tasks. The stranger’s infant was not matched for any other demographic features such as ethnicity or gender. Each 7.5-min run comprised 6 blocks with five 15s trial conditions: four infant videos (own/other infant positive/negative tasks, abbreviated InfOwnPos, InfOwnNeg, InfOtherPos, InfOtherNeg) and a black screen (Rest). Trials were fully randomized within blocks, with each block including one instance of each trial type (See Fig 1). Presentation^®^ software [32] was used to display stimuli.

### 2.4. Valence of infant videos

As originally reported in Laurent, Wright, and Finnegan [23], immediately after scanning mothers re-watched videos and provided valence ratings from -100 (maximum negative) to +100 (maximum positive) with 0 as the neutral point, and intensity ratings from 0 to +100 (maximum intensity).

Although arm-restraint did not on average evoke strong negatively valenced emotions in mothers (*μ* = 22.39, 95% CI = [0.46, 44.33]) or perceptions of negative infant emotions (*μ* = 3.18, 95% CI = [-21.51, 27.87]), peekaboo videos evoked significantly positive valence maternal emotions (*μ* = 85.86, 95% CI = [74.40, 97.35]) and perceptions of positive infant emotions (*μ* = 69.42, 95% CI = [53.62, 85.21]). There was a clear difference in relative valence between conditions: mothers *t*(22) = 5.44, *p* < .001, Cohen’s *d* = 1.57 (95% CI .90–2.24); infants *t*(21) = 4.20, *p* < .001, *d* = 1.41 (95% CI 0.74–2.07). Thus, contrasts represent difference in emotional valence processing, with one task relatively more positive and the other more negative. Comparisons across mother/infant intensity ratings showed no significant differences between stimuli.

### 2.5. Image acquisition

All imaging occurred during a single session at the University of Oregon Robert and Beverly Lewis Center for Neuroimaging using a 3T Siemens Allegra 3 magnet and standard 32-channel phase array birdcage coil. Following a shimming routine to optimize signal-to-noise ratio, a fast localizer scan (FISP), Siemens Autoalign routine, field mapping, functional runs, and anatomical scan were completed.

#### 2.5.1. Functional

T2*-weighted gradient echo sequence, TR = 2000 ms, TE = 30 ms, flip angle = 90°, 32 contiguous slices acquired ascending and interleaved, thickness = 4 mm, 64 × 64 voxel matrix; 226 volumes per run.

#### 2.5.2. Anatomical

T1-weighted 3D MPRAGE sequence, TI = 1100 ms, TR = 2500 ms, TE = 3.41 ms, flip angle = 7°, 176 sagittal slices 1.0 mm thick, 256 × 256 voxel matrix, FOV = 256 mm.

### 2.6. Preprocessing

Functional images were preprocessed using SPM12 (v.6906) [33] with MATLAB 2018a running on Windows 10. If not otherwise stated, default values or recommended values provided by the batch interface were used. First, slice-timing correction interpolated values to the midpoint collected slice. Realignment used a 2-pass procedure, calculating the mean image and realigning images to the mean. A 2mm/2° framewise displacement threshold was established with subjects discarded if > 40% of volumes in any run exceeded threshold. Images were unwarped to correct for image distortion caused by gradient inhomogeneities and a mean image calculated. Because subjects were allowed to move between scans, the field map was not used, and no phase correction was applied. The anatomical MPRAGE image was then coregistered to the mean image from unwarping. A set of warps calculated using the anatomical image was applied to functional images to map them into MNI space. Finally, an 8mm FWHM Gaussian smoothing kernel was applied to all images.

### 2.7. fMRI analysis

#### 2.7.1. Main effects

Functional data were analyzed in a 2-level process. For each subject a General Linear Model (GLM) was estimated at each voxel using restricted maximum likelihoods (ReML). Stimuli as specified above—InfOwnPos, InfOwnNeg, InfOtherPos, InfOtherNeg, and Rest—were modeled using the SPM12 canonical hemodynamic response function. Variations in hemodynamic responses were accounted for with time and spatial dispersion derivatives. Regressors of no interest included a global approximate AR(1) autocorrelation model to account for serial correlations, and scanner drift was fitted with a Discrete Cosine Transform basis (128s cut-off). Head motion was modeled using 6 movement estimation parameters from realignment, and high motion volumes deweighted with indicator variables for volumes exceeding 2mm/2° framewise displacement. All runs from both tasks were included in a single model with regressors from the emotional faces task treated as regressors of no interest.

Contrasts were created for each participant with regressor coefficients averaged across runs. Contrast images were passed to a 1-sample t-test to examine group-level whole-brain voxelwise effects. Statistical maps were thresholded with clusterwise inference using a cluster-defining threshold (CDT) of *p <* .001 (uncorrected) and FWE-corrected cluster threshold of *p <* .05, which has demonstrated reasonable control for false positives [34]. See S1 File for a list of all main effects tested and results. Clusters were labeled using the AAL atlas [35]. To address the aim of identifying psychopathology-related differences in the way a mother responds to her own infant in more positive versus negative contexts, the main effect of interest was (InfOwnPos > InfOwnNeg).

#### 2.7.2. Association with affective psychopathology

To examine how (InfOwnPos > InfOwnNeg) related to anxiety and depression, individual-level contrasts were submitted to ReML multiple regression analysis using the following sets of predictors, tested in separate models: **1.** CESD[T1-T4], **2.** BAI[T1-T4], **3.** Dep-Hx, Dep-Dx, **4.** Anx-Hx, Anx-Dx. Depression and anxiety terms were also included in joint models, and potential “dosage” effects explored by testing summed scores of past and current diagnoses. Results were consistent with the simplified models and are not reported in detail here (see *S1 File*). All models included an intercept term, and continuous psychological covariates were mean-centered. Contrasts tested significance of each model regression weight in both positive and negative directions. Again, images were thresholded with a CDT of p < .001 (uncorrected) and cluster threshold of p < .05 (FWE-corrected).

A *post hoc* exploratory analysis was conducted to examine how depression and anxiety relate to positive and negative contexts individually. Regression coefficients (*β* values) for InfOwnPos and InfOwnNeg were extracted for each subject from clusters determined by negative relationships of (InfOwnPos > InfOwnNeg) with BAI[T1-4] and CESD[T1-4]. The Marsbar toolbox (v.0.44) [36] was used to extract average *β* values, producing a representative value for each subject-cluster. These were plotted against the psychological variable that produced the clusters to visually inspect direction of change. BAI[T1-T4] and CESD[T1-T4] were chosen for illustration because they showed extensive relations with maternal responsiveness.

## 3. Results

### 3.1. Main effects

The contrast of (InfOwnPos > InfOwnNeg) did not demonstrate significant clusters after correction (see S1 File for all main effects).

### 3.2. Associations with depression

**Fig 2:**
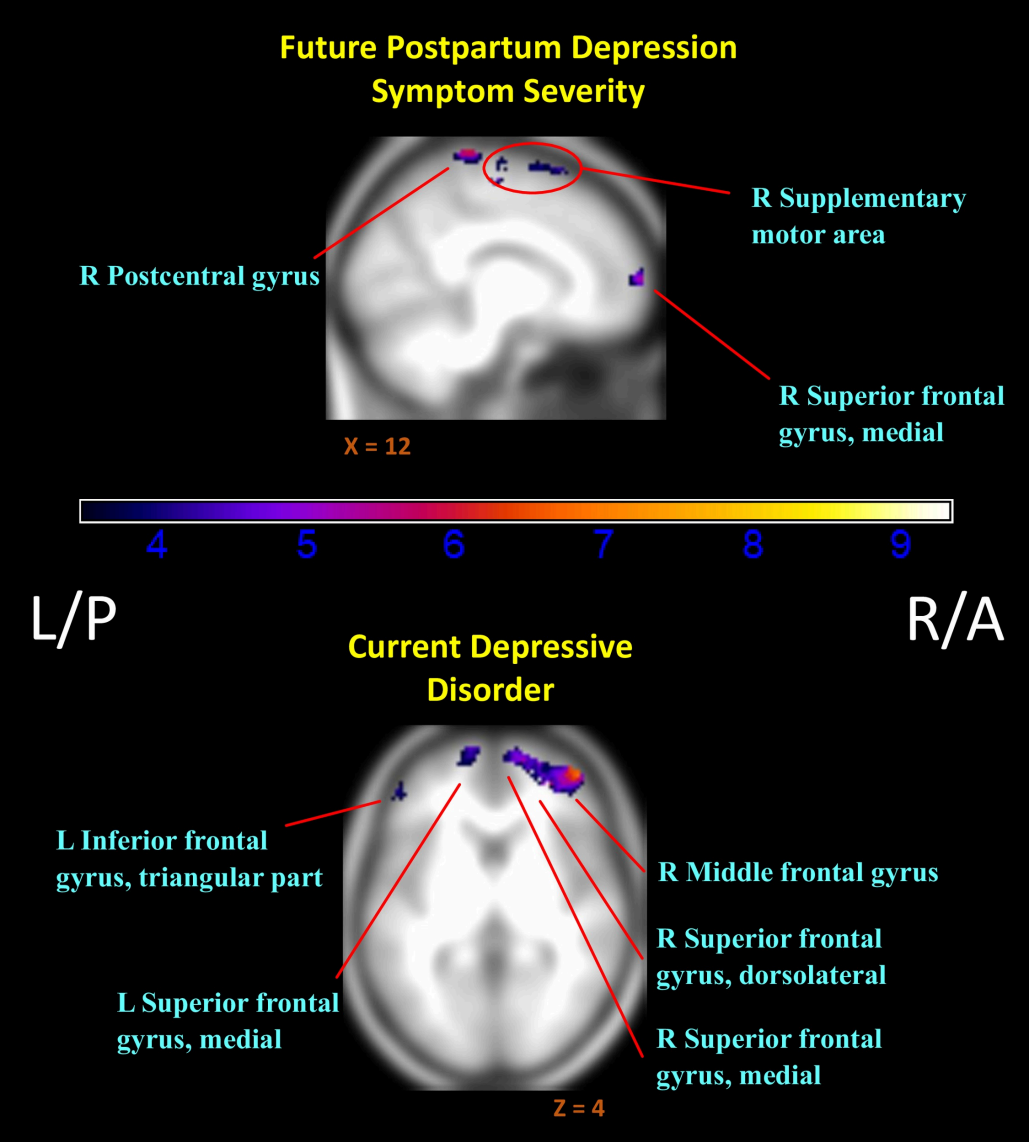
Regions of reduced activation to positive versus negative infant emotion tasks with increasing markers of depression. (*i*.*e*. Negative associations of (InfOwnPos > InfOwnNeg) with CESD[T1-T4] & Dep-Dx. *Anatomical labels added for reference are taken from the AAL atlas*, *but do not represent an exhaustive list of cluster extent*. *Activation overlaid on smoothed (8mm Gaussian kernel) average of subject anatomical images*. *Past episodes of depression did not relate to differential responding for this contrast*.

Mothers who later reported greater depression symptoms showed predominantly lower relative activation to their own infant in positive versus negative contexts (negative association of InfOwnPos > InfOwnNeg with CESD[T1-T4]), with reductions most evident in the right superior frontal gyrus and some clusters overlapping the parietal lobe and caudal portions of the frontal lobe (Fig 2). See Table 3 for an abbreviated summary of cluster labels and S1 File for an expanded list.

**Table 3:** Model of estimated future postpartum depression. Brain regions showing differences in maternal activation to own infant in positive versus negative emotion tasks (InfOwnPos > InfOwnNeg) related to increasing postpartum depression symptoms (CESD[T1-4]–estimated postpartum average depression).

Cluster	Regions	Number of voxels in region	Cluster size	FWE-corrected p-value
***positive association with CESD Time1-4 Intercept***			*cluster extent threshold 537 voxels*.	
1	R Cerebellum 6	203	537	< 0.001
	OUTSIDE	119		
***negative association with CESD Time1-4 Intercept***			*cluster extent threshold 124 voxels*.	
1	L Paracentral lobule	272	1363	< 0.001
	R SFG, dorsolateral	176		
	L Postcentral gyrus	174		
	R Precentral gyrus	132		
2	R Postcentral gyrus	329	950	< 0.001
	OUTSIDE	200		
3	OUTSIDE	295	345	< 0.001
4	L Inferior temporal gyrus	53	168	0.004
	OUTSIDE	37		
5	R SFG, dorsolateral	120	151	0.006
6	R SFG, dorsolateral	64	124	0.017
***negative association with Model Intercept***			*cluster extent threshold 127 voxels*.	
1	R Insula	76	127	0.015

**Note.** *Only regions of largest coverage up to half the cluster are reported for brevity*. *See* S1 *File for full table*. *SFG = Superior frontal gyrus; OUTSIDE = not defined in the AAL atlas*. *Whole-brain analysis*. *Primary voxel-wise correction at p <* .*001 (unc*.*) for cluster definition; significance threshold T(22) = 3*.*50; Cluster-level correction conducted at p <* .*05 (FWE)*. *Clusters labelled using AAL atlas*. *Labels listed in decreasing order of volume coverage of cluster*.

Current depression also related to widespread reductions in differential response (negative association of InfOwnPos > InfOwnNeg with Dep-Dx). As shown in Table 4 and Fig 2, this expanded over the right (dorsolateral) superior, middle, and inferior frontal gyri, left inferior and middle temporal lobe, and bilateral angular gyri. A history of depressive episodes did not independently impact relative (InfOwnPos > InfOwnNeg) neural response.

**Table 4:** Model of current and past depressive disorders. Brain regions showing differences in maternal activation to own infant in positive versus negative emotion tasks (InfOwnPos > InfOwnNeg) related to current diagnosis of depression.

Cluster	Regions	Number of voxels in region	Cluster size	FWE-corrected p-value
***negative association with Current Depression***			*cluster extent threshold 97 voxels*.	
1	R SFG, dorsolateral	990	4185	< 0.001
	R Postcentral gyrus	504		
	R Angular gyrus	425		
	R Middle frontal gyrus	392		
2	L Postcentral gyrus	616	2503	< 0.001
	L Precentral gyrus	489		
	OUTSIDE	448		
3	R Middle frontal gyrus	577	1355	< 0.001
	R IFG, triangular part	228		
4	L Middle temporal gyrus	106	309	< 0.001
	OUTSIDE	86		
5	L IFG, triangular part	280	294	< 0.001
6	L Inferior temporal gyrus	92	130	0.011
7	OUTSIDE	59	114	0.021
8	L Angular gyrus	56	113	0.021
	L Middle occipital gyrus	41		
9	L SFG, medial	49	97	0.042

**Note.** *History of depressive episodes was not associated with change in neural response between conditions*. *Only regions of largest coverage up to half the cluster are reported for brevity*. *See* S1 *File for full table*. *SFG = Superior frontal gyrus; IFG = Inferior frontal gyrus; OUTSIDE = not defined in the AAL atlas*. *Whole-brain analysis*. *Primary voxel-wise correction at p <* .*0001 (unc*.*) for cluster definition; significance threshold T(21) = 3*.*53; Cluster-level correction conducted at p <* .*05 (FWE)*. *Clusters labelled using AAL atlas*. *Labels listed in decreasing order of volume coverage of cluster*.

### 3.3. Associations with anxiety

**Fig 3:**
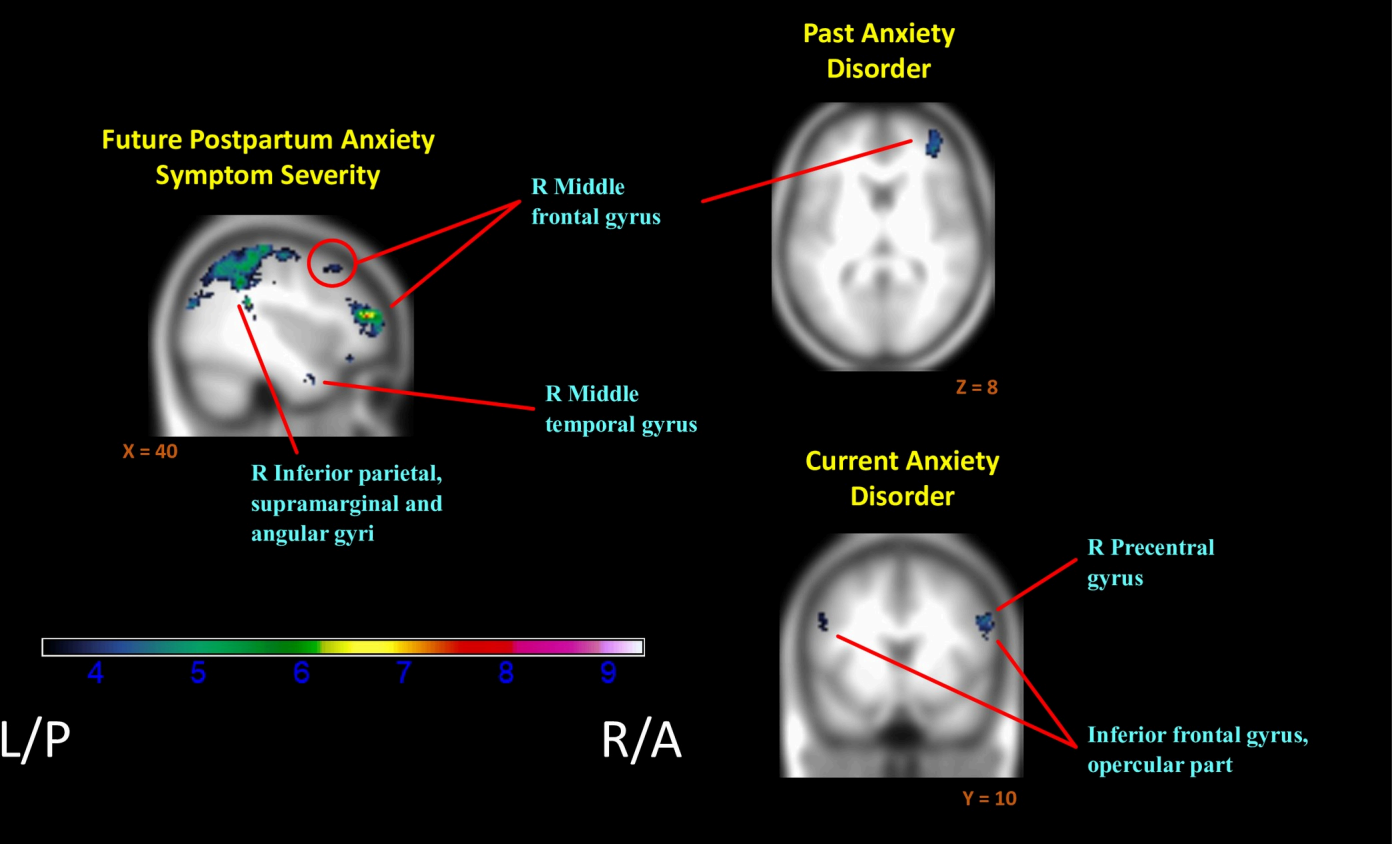
Regions of reduced activation to positive versus negative infant emotion tasks with increasing markers of anxiety. (*i*.*e*. Negative associations of (InfOwnPos > InfOwnNeg) with BAI[T1-T4], Anx-Hx, & Anx-Dx). *Anatomical labels added for reference are taken from the AAL atlas*, *but do not represent an exhaustive list of cluster extent*. *Activation overlaid on smoothed (8mm Gaussian kernel) average of subject anatomical images*.

Greater subsequent or “future” anxiety also related to extensive decreases in relative neural activation to positive versus negative contexts (InfOwnPos > InfOwnNeg). This included bilateral postcentral gyri and adjacent fronto-parietal areas, left middle temporal and right parahippocampal gyri, and right superior and middle frontal gyri (Fig 3). See Table 5 and S1 File for cluster labels.

**Table 5:** Model of estimated future postpartum anxiety. Brain regions showing differences in maternal activation to own infant in positive versus negative emotion tasks (InfOwnPos > InfOwnNeg) related to increasing postpartum anxiety symptoms (BAI[T1-4]–estimated postpartum average anxiety).

Cluster	Regions	Number of voxels in region	Cluster size	FWE-corrected p-value
***negative association with BAI Time1-4 Intercept***			*cluster extent threshold 99 voxels*.	
1	OUTSIDE	2823	17043	< 0.001
	R Postcentral gyrus	1082		
	L Postcentral gyrus	1057		
	R SFG, dorsolateral	995		
	L IP, supramarginal and angular gyri	886		
	L Paracentral lobule	867		
	R Supplementary motor area	849		
	L Precentral gyrus	669		
	R Superior parietal gyrus	659		
	R Precentral gyrus	633		
	L Superior parietal gyrus	604		
2	R Middle frontal gyrus	536	1017	< 0.001
3	L Middle temporal gyrus	63	197	< 0.001
	L Fusiform gyrus	47		
4	L Crus Cerebellum 2	110	164	0.002
5	OUTSIDE	65	147	0.005
	R IFG, orbital part	57		
6	L Middle occipital gyrus	107	129	0.009
7	R Parahippocampal gyrus	81	117	0.015
8	OUTSIDE	55	106	0.024
9	OUTSIDE	43	99	0.033
	R Temporal pole: MTG	31		
***negative association with Model Intercept***			*cluster extent threshold 117 voxels*.	
1	R Insula	75	117	0.015

**Note.** *Only regions of largest coverage up to half the cluster are reported for brevity*. *See* S1 *File for full table*. *SFG = Superior frontal gyrus; IP = Inferior parietal; IFG = Inferior frontal gyrus; MFG = Middle frontal gyrus; MTG = Middle temporal gyrus; STG = Superior temporal gyrus; OUTSIDE = not defined in the AAL atlas*. *Whole-brain analysis*. *Primary voxel-wise correction at p <* .*0001 (unc*.*) for cluster definition; significance threshold T(22) = 3*.*50; Cluster-level correction conducted at p <* .*05 (FWE)*. *Clusters labelled using AAL atlas*. *Labels listed in decreasing order of volume coverage of cluster*.

Consistent with the above, both current and past anxiety disorders were associated with an overall reduction in mothers’ relative neural response (Table 6, Fig 3). Past anxiety predicted reductions in the right frontal areas with superior, middle and inferior frontal gyri implicated. Current anxiety exhibited more widespread effects than anxiety history and was further associated with reduced differential responding in parietal and temporal regions, along with left inferior frontal and bilateral middle frontal gyri.

**Table 6:** Model of current and past anxiety disorders. Brain regions showing differences in maternal activation to own infant in positive versus negative emotion tasks (InfOwnPos > InfOwnNeg) related to current diagnosis or history of anxiety disorders.

Cluster	Regions	Number of voxels in region	Cluster size	FWE-corrected p-value
***positive association with Anxiety History***			*cluster extent threshold 139 voxels*.	
1	R Cerebellum 6	101	139	0.007
	OUTSIDE	33		
	R Cerebellum 8	5		
***negative association with Anxiety History***			*cluster extent threshold 106 voxels*.	
1	R Middle frontal gyrus	159	181	0.002
	OUTSIDE	16		
	R IFG, triangular part	4		
	R SFG, dorsolateral	2		
Clusters Spanning a Single Region	OUTSIDE		106	0.028
***negative association with Current Anxiety***			*cluster extent threshold 102 voxels*.	
1	R Precentral gyrus	128	195	0.001
	R Middle frontal gyrus	43		
	OUTSIDE	24		
2	L Precentral gyrus	100	160	0.003
	L IFG, opercular part	29		
	L IFG, triangular part	24		
	L Middle frontal gyrus	5		
	L Postcentral gyrus	2		
3	OUTSIDE	43	142	0.007
	R Angular gyrus	41		
	R Middle temporal gyrus	40		
	R Superior temporal gyrus	18		
4	L Middle occipital gyrus	125	135	0.009
	OUTSIDE	10		
5	R IFG, opercular part	53	102	0.033
	R Precentral gyrus	49		

**Note.** *Only regions of largest coverage up to half the cluster are reported for brevity*. *See* S1 *File for full table*. *IFG = Inferior frontal gyrus; SFG = Superior frontal gyrus; OUTSIDE = not defined in the AAL atlas*. *Whole-brain analysis*. *Primary voxel-wise correction at p <* .*0001 (unc*.*) for cluster definition; significance threshold T(21) = 3*.*53; Cluster-level correction conducted at p <* .*05 (FWE)*. *Clusters labelled using AAL atlas*. *Labels listed in decreasing order of volume coverage of cluster*.

### 3.4. Syndromal specificity

**Fig 4:**
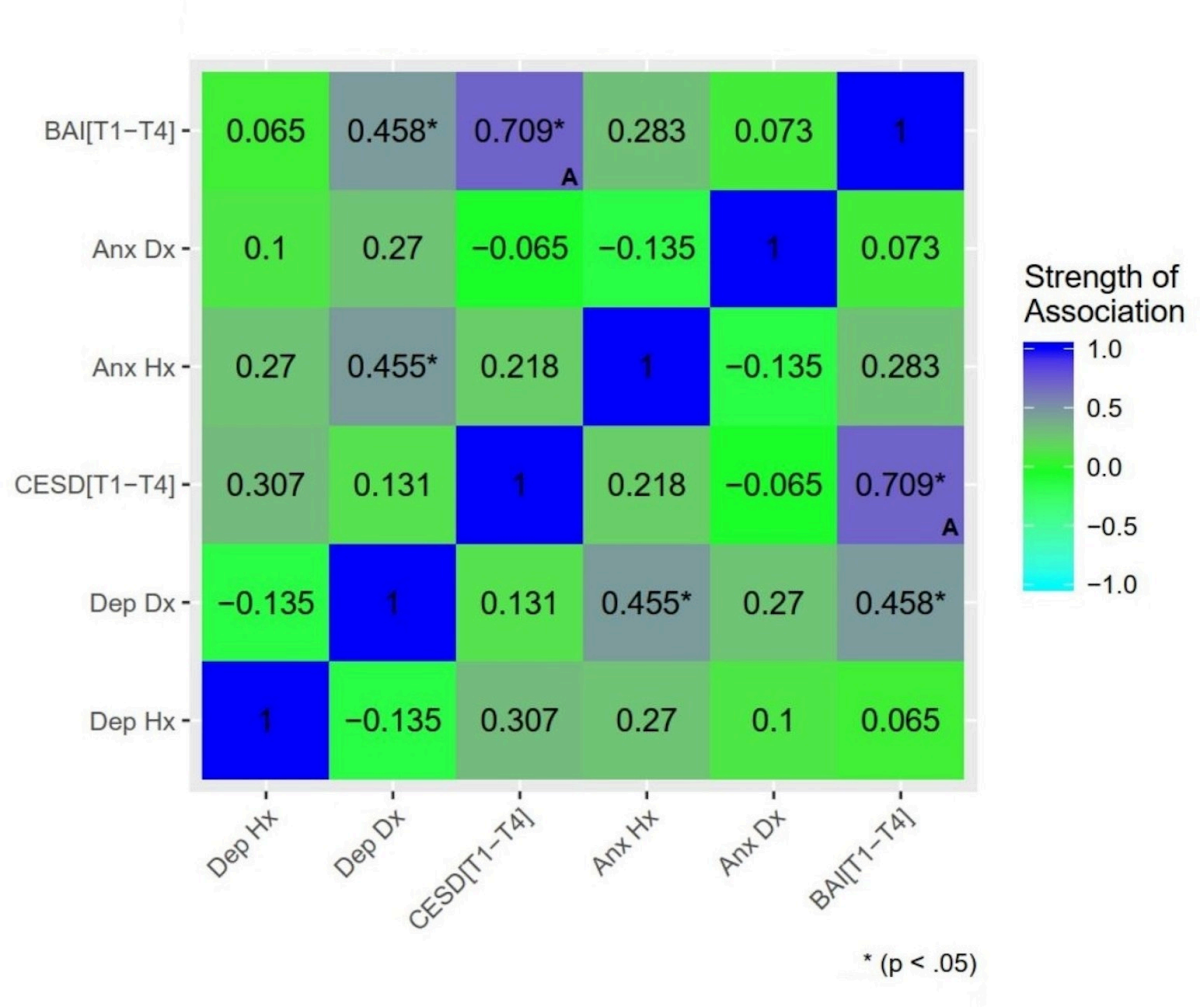
Associations between markers of psychopathology: Past, present, and future depression and anxiety. *Dep Hx = history of depressive disorder based on the Structured Clinical Interview for DSM-IV (SCID); Dep Dx = current depressive disorder; Anx Hx = anxiety disorder history; Anx Dx = current anxiety disorder; CESD[T1-4] = estimated mean CESD score across all assessment time points (i*.*e*. *Estimated Future Depression Symptom Severity); BAI[T1-4] = estimated mean BAI score (i*.*e*. *Estimated Future Anxiety Severity)*. *Significance delineated at uncorrected thresholds*. **(A)** *Denotes Pearson’s correlation between a continuous-continuous pair*. *All other associations reported (categorical-categorical or categorical-continuous pairs) are reported using Spearman’s rank correlation*.

Extensive overlap between regions of differential activation associated with depression and anxiety suggests effects are best attributed to a common internalizing distress component, rather than distinct syndromal components. To further probe specificity, a model including both prospective depression and anxiety symptoms was estimated (see S1 File). Lower differential activation was associated with BAI[T1-4] only, suggesting effects related to postpartum depression were driven by shared variance with anxiety. The reduction (over 50%) in cluster extent further suggests a substantial proportion of postpartum anxiety effects were driven by shared variance with depression. Given the collinearity of these predictors (Fig 4) and absence of regularization, results should be interpreted with caution. Future work either employing factor analytic models dissociating the shared and unique variance of depression/anxiety symptoms or construction of clear group delineations of anxiety-only and depression-only may help clarify this. In addition, regression models with mean subject-level contrast values (InfOwnPos>InfOwnNeg) from the clusters identified in the above analysis tested whether T1 maternal brain response predicted T4 depression or anxiety over and above T1-T4 maternal stress (life events and parenting stress). Maternal brain response measures continued to significantly predict later symptoms with no meaningful reduction in effect size when controlling for stress, supporting independent prospective effects of infant-directed brain response on maternal psychopathology.

### 3.5. Direction of relationship with anxiety and depression

**Fig 5:**
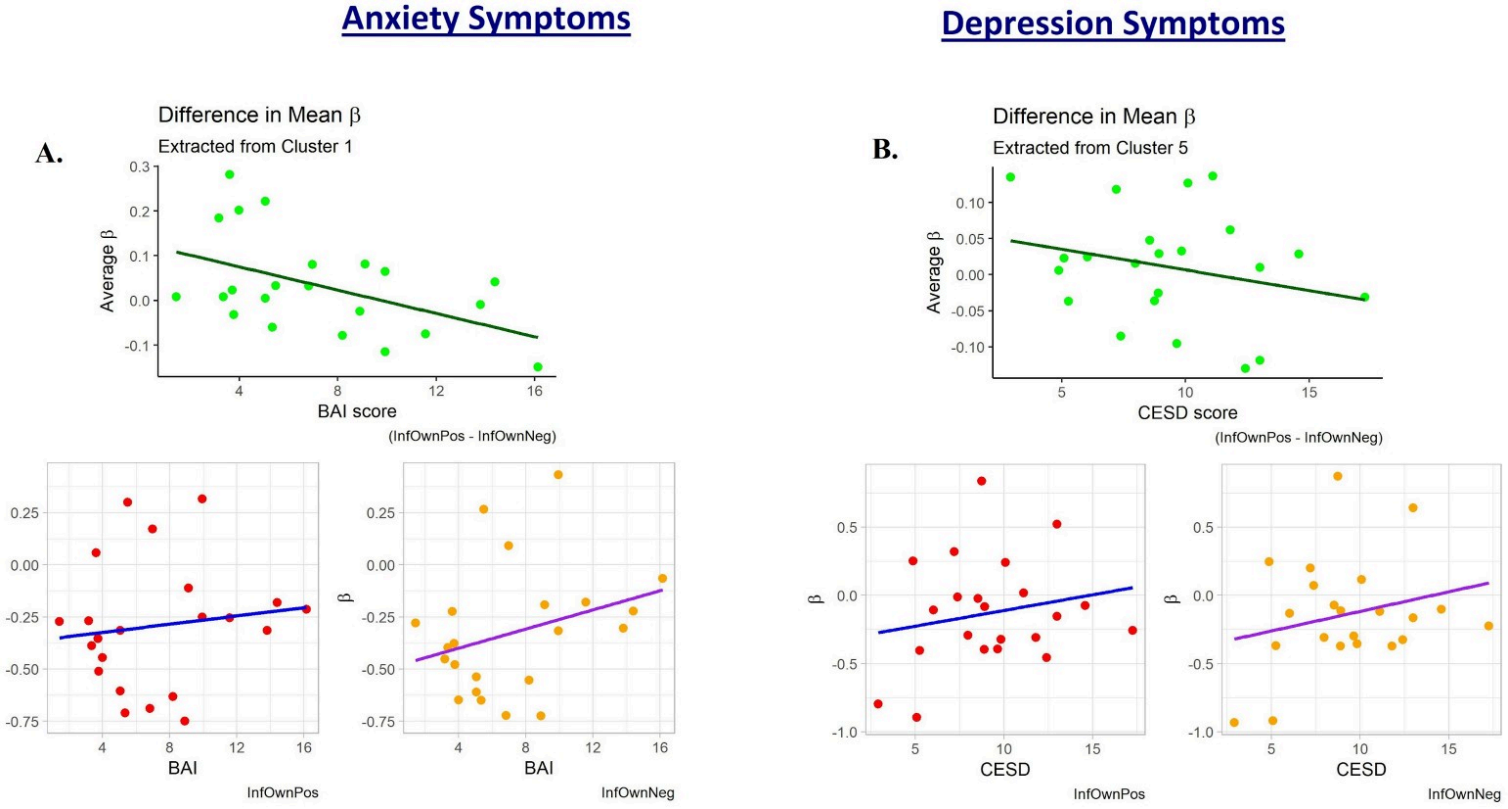
Plots of the subject-level average regression coefficient predicting maternal neural response to own infant positive and negative emotion tasks. One subject with an elevated CESD[T1-T4] and two with elevated BAI[T1-T4] scores appeared to deviate from the general trend of their less symptomatic peers. The plots are displayed with these potential outliers removed. **A.)** shows these values plotted against participant anxiety scores (BAI). This cluster was defined from the regression against BAI and is a large area covering primarily parietal lobe structures. The upper plot is the difference between these two mean coefficients for each subject and is reflective of the general decreases observed in the whole-brain analysis. **B.)** shows a similar trend when coefficients are plotted against depression scores (CESD). This cluster was defined from the regression against CESD and is located mostly within the right dorsolateral superior frontal gyrus.

To probe the source of mothers’ reduced relative neural activation with increasing affective psychopathology, exploratory plots of the mean InfOwnPos and InfOwnNeg *β* values for each cluster against BAI[T1-T4] and CESD[T1-T4] were created. These revealed general upward trends of both coefficients with increasing symptoms across most clusters, but this was especially pronounced for the negative situation. Fig 5 provides a representative plot.

## 4. Discussion

The current findings point to a relation between maternal brain response–specifically, decreasing relative activation to positive compared to negative infant emotion situations–and a shared internalizing component of postpartum symptoms that may fuel ongoing distress. This was related in varying degrees to past, current, and future affective psychopathology; the strongest effects involved prospective associations with anxiety symptoms and concurrent associations with diagnosed depression, alongside more limited effects of past anxiety and nonsignificant effects of past depression. This presents a picture in which postpartum depression plays a role in the mother’s ability to differentially process the emotional cues of her child, with anxiety having a more prominent bidirectional longitudinal relationship to brain response. Despite some regions of unique covariance, largely overlapping clusters support the idea that biased brain responding is involved in a common internalizing symptom dimension. At the same time, the preponderance of concurrent/prospective psychopathology effects implicate disruptions in maternal response to their infants specifically in the perpetuation of postpartum symptoms, as opposed to more general phenotypic or scarring effects. However, a more careful examination of unique components of anxiety and depression may yield evidence for differential associations with outcomes and patterns of brain activity (see [37]).

Exploratory plots of regression coefficients within significant clusters suggested differences were due to steeper increases in response to the negative situations with increasing affective symptoms. It appears framing maternal postpartum depression (or anxiety) as primarily a deficit in reward responsiveness is overly simplistic, and preferential sensitization to negative emotional contexts with their infants may better capture the nature of response imbalance. The current analysis does not allow us to discern whether this represents a homogenization (response to positive and negative context becoming more alike) or an exaggeration of differential response. Further investigation to clarify the nature of this response bias, as well as when it emerges and how it may relate to mother/infant cognitions and behaviors, is needed. For example, a maternal response bias toward more negative/stressful emotion situations with her infant may heighten mutual physiological dysregulation [38, 39] while impairing attunement to infant emotional needs and behavioral synchrony that would help repair mother/infant wellbeing [40]. Over time, repeated failures in behavioral synchrony in turn shape the child’s risk for emotion dysregulation and disorder, in part through changes in the child’s neural processing of social stress/distress [41, 42]. As noted by other researchers, such shifts in the mother’s neural processing—perhaps based on her own history of nonoptimal caregiving—may contribute to disruptions in maternal reflective functioning and mother-infant attachment that put the child at further risk of psychological difficulties later in life [43, 44]. *Post hoc* tests exploring the role of neural responsivity to her own infant compared to another infant when controlling for valence (See S1 File sections 5.D. and 5.E) revealed associations with internalizing distress in similar regions, supporting the idea that ongoing postpartum depression and anxiety are associated with specific deficits in maternal response bias to her own child (as opposed to general disfunction in socio-emotional processes). Notably, these follow-up analyses also contained clusters not found in the contrasts isolating differences in valance functioning, warranting future work disentangling the relationship between affective distress and specific components of the maternal response pattern. Thus, the currently detected associations should be used to guide further exploration of upstream (maternal response bias) mechanisms that may contribute to a broader chain of dyadic dysregulation and psychopathology transmission.

Regions of lower relative activation in distressed mothers comprised a number of prefrontal areas, notably the left medial and right medial orbital sections of the superior frontal gyrus (SFG). This area is hypothesized to coordinate task set switching [45], a cognitive function known to be impaired in unipolar depression [46] and thought to reflect inability to inhibit processing of prior information [47]. The medial SFG has also been observed to coordinate activity with other frontal lobe areas and limbic structures during successful emotion regulation [48], and the left medial prefrontal cortex in particular shows preferential activity toward sad videos in depressed compared to non-depressed participants [49]. Our paradigm required mothers to switch between viewing positive and negative emotional contexts of infant interaction; as such, the disrupted medial SFG function observed could represent a difficulty in shifting the response repertoire needed to successfully meet her infant’s needs in each context and/or a larger breakdown in emotional regulation of response to her child’s emotions. Future work examining coordinated functional activity may help resolve the role of the SFG in the brain response of a mother to her child.

We also observed reduced relative response in the inferior frontal gyrus (IFG) related to past, current, and future anxiety and current and future depression. Increased IFG response to negatively valenced facial expressions has been associated with early (within 48 hours of birth) postpartum anxiety symptoms and later (4–6 weeks) postpartum depression symptoms in prior region-of-interest analyses [19]. The explanation offered by these researchers that exaggerated IFG engagement represents an exacerbation of normative postpartum increases in emotional reactivity and/or inability to scale back inhibitory control demands when confronting negatively valenced stimuli may apply to the current results. This could potentially provide a neural basis for the behavioral patterns observed in depressed and anxious mothers interacting with their infants. For depressed mothers, interactions are often characterized by less maternal engagement and emotional availability, or conversely by increased intrusiveness with the infant (with a subset of depressed mothers displaying normative interactions) [50]. Similarly, anxious mothers exhibit increased intrusiveness [51] and impaired sensitivity in responding to infant needs [52]. Which behaviors predominate could be a result of the particular balance of increasing maternal neural response to negative and/or decreasing response to positive cues during infant interactions. Subsequent reactions of the infant (*e*.*g*. avoidance of eye contact, reduced emotional expressiveness, less contingent responding [50, 53]) could serve to exacerbate already biased neural processing of infant cues with implications for chronicity of symptoms. This would suggest more far-reaching links between this neural response pattern and both prior and future affective psychopathology. Further work is needed to elucidate the relationship between maternal brain response, mother-infant interactions, and the development of psychopathology in both mother and child. Such avenues could provide a rich source of information to allow pediatricians and other health providers to identify and provide additional resources to promote resilience in dyads that may be at risk for development of psychological disorders.

Consistent with some [18], but not all [12], prior work involving mothers, we did not find associations between anxiety or depression and differential response in the amygdala or insula. We also did not find evidence that affective distress involved differences in neural activity in the thalamus, and minimal evidence for involvement of the caudate and putamen—areas that, together with the amygdala and insula, were implicated in the literature as reward processing regions with preference for one’s own infant that may be impacted by depression [7, 9, 11]. If postpartum depression and/or anxiety primarily represented a deficit in a mother’s ability to process the reward-inducing positive emotions of her infant’s socio-emotional cues as originally hypothesized, we would have expected more prominent changes in differential response to positive vs negative infant cues in these regions. This was not supported by the current results and further strengthens the conclusion that postpartum affective distress is not simply a reduced responsiveness to reward.

It is notable that several regions implicated in this analysis did not fully align with our original hypotheses which identified the basal ganglia, thalamus, amygdala, insula, and inferior frontal gyrus as potential regions impacted. Although some regions, such as the IFG were consistent with prior work, others like the SFG were unexpected. Much of the preexisting literature examining peripartum affective disorders has focused on functional masking or region-of-interest analyses to target hypothesized brain structures (for example, see the work of [54–58]). While this has the advantage of increased statistical power, it also means that many of the potential changes in neural activity across the postpartum brain have been left unexamined. This could have driven some of the divergence between the hypothesized brain regions and our results. More work examining whole-brain response may help to elucidate these discrepancies.

Limitations to the current study include a modest sample size of research participants, the somewhat mild of intensity of the stimuli, the low prevalence of clinically significant depression and anxiety (with symptom-level severity predominantly in subclinical ranges), and the potential existence of explanatory confounds (for example, trauma exposure). Although our sample size is typical of this research domain (our sample size is either greater than or comparable to those reported in [12, 54–57, 59, 60]), it is well known that the standard error of regression coefficients increases with decreasing sample size. Thus, test statistics derived from small sample designs may exhibit more variability upon replication. This study included several strengths known to increase metrics of reliability, such as the use of a naturalistic stimuli [61], collecting data over two runs (to minimize participant fatigue) [62], and block design with target vs non-target contrasts [61]. While the lower bounds of reliability for task-based fMRI has been purported to be low [63], the degree of improvement provided by design and analysis considerations is still an active area of debate [64] with potential for achieving high levels of reliability. The results of this work represent the first attempt to characterize the relationship between neural response to infants and the developmental course of postpartum depression and anxiety, but future replication will be essential for establishing the most robust predictors of symptom trajectories. We address the implications of the other identified limitations in the discussion that follows.

The current null findings may also be attributable to the relatively mild nature of the emotional tasks with which mothers were presented or the subclinical levels of maternal distress in the majority of cases. Future stimuli could involve a prescreening process for emotional intensity and valence similar to that employed by [65] to ensure only the highest rated samples are chosen. Employing probe measures during the task may facilitate greater engagement with the stimuli. Future investigation could also assess the role of habituation to the stimuli as a source of decreased responsivity through more detailed time series analysis. Clearly, more work involving a range of infant emotional stimuli and maternal clinical presentations will be needed.

Taken in the context of relations among psychopathology indicators in this sample, these findings may point to a particular risk profile in women. In particular, mothers’ concurrent (3-month) depression diagnosis was most strongly associated with prospective (3–18 month) anxiety symptoms, and to a lesser extent with past anxiety diagnosis (See Fig 4). Certain women may show a profile of sensitivity to interpersonal stress or challenge that gives rise to problems with anxiety prior to parenthood, leading to a depressive episode in the early postpartum as the mother struggles to adjust to infant emotional demands, and continuing as a pervasive sense of stress/distress going forward as the child develops. It is important to note that elevated symptoms did not correlate with difference in maternal self-report of their emotional response or assessment of their infant’s emotions. That is, mothers did not necessarily *think* their baby was more negative in these situations, but rather, their brains *responded differently* to viewing their babies. This dissociation from conscious appraisal of their infant’s or their own emotions highlights the importance of considering brain measures to detect underlying response biases that may help drive postpartum psychopathology.

It should be noted that this work restricted analysis to the constructs of depression and anxiety, which may overlap with other unexplored sources of the observed bias in neural response. In particular, the role of trauma exposure and trauma symptoms has not been fully separated from neural responses related to disordered affective processing. Traumatic stress exposure is known to affect maternal behavior in both humans and rodent models [66–69] and post-traumatic stress disorders are highly comorbid with depression and anxiety [70] (although this was not evident in our sample; see S1 File). Previous work by [71] found mothers with PTSD displayed greater differential response in the entorhinal cortex and caudate to videos of their own toddlers during separation compared to free play. Reduced difference was also noted in the superior frontal gyrus and bilateral superior parietal lobes, both regions in this study that displayed an altered magnitude of differential response (although in the opposite direction) with greater severity of future depression or anxiety. It is possible that trauma exposure may provide a common source of covariance, potentially mediated by physiological changes to homeostatic set points such as changes to HPA stress reactivity [72]. Teasing apart the potential causal mechanisms for our reported observations will be essential for understanding how brain response may influence, or be influenced by, affective symptom trajectories. Future work will also be needed for replication and validation of these findings; however, such neural markers of differences in maternal response to their infants may be useful as additional evidence to aid in identifying mothers at risk for worsening depression and anxiety. Combined with information on other risk factors for postpartum depression, it may be possible to construct a “risk score” that pinpoints mothers most in need of additional support services. Risk scores could in turn allow health providers to maximize resource utilization by targeting early interventions to mothers that are particularly vulnerable.

Our study focused on a small non-clinical cohort with a relatively low incidence of past anxiety and current depression (each comprising about 8% of the sample), and future work should assess whether these observations apply to higher levels of psychological distress. Ideally such work would be complemented with behavioral observations, which may help to interpret the disconnect between maternal self-reported emotions and changes in brain response with depression or anxiety. It will also be important to determine how early identification and intervention on these patterns could impact postpartum symptom trajectories. Although we did not find strong evidence for an association of maternal brain response with history of psychopathology, future work in larger and more diagnostically varied samples could help make finer-grained distinctions regarding effects that depend on the recency, severity, or number of earlier episodes.

Based on the results highlighted here, intervention approaches that help mothers shift attention toward pleasant/potentially rewarding interactions with their infant while lowering the level of perceived threat and/or effortful regulation of response to more difficult/potentially distressing interactions should be explored. Mother-infant interventions that aim to increase maternal savoring of positive emotional experiences and offer tools to regulate negative emotions, such as mindfulness-based childbirth and parenting programs, may prove particularly beneficial (See [73] and [74] for a review of evidence-based interventions for mothers with young children). We provide here some of the first observations that alterations in mothers’ relative brain response to valenced infant interactions predict ongoing affective psychopathology in the postpartum. These findings further suggest biased brain responding represents a prospective risk for postpartum psychopathology (as opposed to a more stable phenotypic pattern) that can best be characterized as internalizing distress (as opposed to depression or anxiety). Such insights may allow us to better identify and remediate psychopathology-perpetuating patterns for the good of both parent and child.

## Supporting information

S1 File Detailed results and models not reported in main manuscript. Contains full results of main contrasts and results of regression against psychological variables reported in the main manuscript. This also contains additional participant demographics, analysis of post-scan ratings of infant videos, and models not reported in the main manuscript.
(DOCX)
