# Supplementary material for: Mothers’ neural response to valenced infant interactions predicts postpartum depression and anxiety
Source: PLoS One. 2021 Apr 27;16(4):e0250487. doi: 10.1371/journal.pone.0250487 (PMC8078806; doi:10.1371/journal.pone.0250487)
Supplement: S1 File — Contains full results of main contrasts and results of regression against psychological variables reported in the main manuscript. This also contains additional participant demographics, analysis of post-scan ratings of infant videos, and models not reported in the main manuscript. (DOCX) [file pone.0250487.s001.docx]

**S1 File**

**Table of Contents**

**[I.](#Section_1)** [Results of Main Contrasts](#Section_1)

[**II.** Results of Regression against Psychological Variables](#Section_2)

1. [Regression against (InfOwnPos > InfOwnNeg)](#Section_2_A)

[**III.** Participant Demographics](#Section_3)

1. [Recruitment Strategy](#Section_3_A)
2. [Dropout Comparisons](#Section_3_B)
3. [Contingency Tables of History and Diagnosis Variables.](#Section_3_C)

[**IV.** Post-Scan Rating of Infant Videos](#Section_4)

1. [Correlations among Post-Scan Ratings](#Section_4_A)
2. [Association of Post-Scan Ratings with Psychopathology](#Section_4_B)

[**V.** Models not Reported in Main Manuscript](#Section_5)

1. [Table of Association for Supplementary Models](#Section_5_A)
2. [Regression against (InfOwnPos > InfOwnNeg)](#Section_5_B)
3. [Regression against (InfPos > InfNeg)](#Section_5_C)
4. [Regression against (InfOwnPos > InfOtherPos)](#Section_5_D)
5. [Regression against (InfOwnNeg > InfOtherNeg)](#Section_5_E)
6. [Additional Exploratory Models](#Section_5_F)

**[Section I.](#Table_of_Contents)** [Results of Main Contrasts](#Table_of_Contents)

This section reports the group-level analysis examining differences between stimulus conditions. Results represent whole-brain voxel-wise analysis. Primary voxel-wise correction was used at p < .0001 (unc.) for cluster definition; Cluster-level correction conducted at p < .05 (FWE). All clusters labelled using the AAL^[1]^ atlas with labels listed in decreasing order of volume coverage of cluster.

Below is a summary of all main effects tested. Models showing significant results are in red.

| **Infant Viewing Task** | |
| --- | --- |
| InfOwnPos > InfOwnNeg  [**InfOwnNeg > InfOwnPos**](#Section_1_InfOwnNeg_InfOwnPos)  **[InfOwn > InfOther](#Section_1_InfOwn_InfOther)**  [**InfOther > InfOwn**](#Section_1_InfOther_InfOwn) | **[InfPos > InfNeg](#Section_1_InfPos_InfNeg)**  **[InfNeg > InfPos](#Section_1_InfNeg_InfPos)**  InfPos > Fixation  InfNeg > Fixation |
| *Key: Inf = infant viewing task; Own = mothers’ own infant; Other = stranger’s infant; Pos = peek-a-boo videos; Neg = arm restraint videos; Fixation = rest condition* | |

*Abbreviation Key:*

*IFG = Inferior frontal gyrus; SFG = Superior frontal gyrus; MFG = Middle frontal gyrus; OUTSIDE = not defined in the AAL atlas.*

**[InfOwnNeg > InfOwnPos](#Section_1_Table)**

| **Cluster** | **Regions** | **Number of voxels in region** | **Cluster size** | **FWE-corrected p-value** |
| --- | --- | --- | --- | --- |
| 1 | R Insula OUTSIDE R Rolandic operculum R IFG, opercular part R Putamen | 75 24 12 3 2 | 116 | 0.027 |

*Group level contrast showing greater neural engagement when mothers view their own infants engaged in a negative emotion task compared to viewing them in a positive emotion task. Significance threshold T(23) = 3.48; Cluster extent threshold = 116 voxels.*

**[InfOwn > InfOther](#Section_1_Table)**

| **Cluster** | **Regions** | **Number of voxels in region** | **Cluster size** | **FWE-corrected p-value** |
| --- | --- | --- | --- | --- |
| 1 | OUTSIDE R Caudate nucleus R SFG, orbital part L Caudate nucleus R Olfactory cortex R MFG, orbital part R Anterior cingulate and paracingulate gyri L Olfactory cortex R SFG, medial orbital R Gyrus rectus | 196 45 30 28 27 14 13  9 2 1 | 365 | < 0.001 |
| 2 | R Inferior temporal gyrus OUTSIDE R Middle temporal gyrus R Inferior occipital gyrus R Fusiform gyrus | 180 108 40 26 6 | 360 | < 0.001 |
| 3 | L Crus Cerebellum 1 L Crus Cerebellum 2 L Cerebellum 8 L Cerebellum 7b OUTSIDE L Cerebellum 9 L Cerebellum 6 | 200 51 35 35 13 4 4 | 342 | < 0.001 |
| 4 | OUTSIDE L Cerebellum 4-5 L Cerebellum 3 | 190 22 11 | 223 | < 0.001 |
| 5 | OUTSIDE L Posterior cingulate gyrus L Lingual gyrus L Cerebellum 4-5 L Precuneus L Calcarine fissure  Vermis 4-5 | 73 46 41 34 17 7 2 | 220 | < 0.001 |
| 6 | Vermis 6 L Cerebellum 6 Vermis 7 L Crus Cerebellum 1 OUTSIDE L Cerebellum 4-5 L Crus Cerebellum 2 | 95 95 9 6 2 1 1 | 209 | 0.001 |
| 7 | L Superior occipital gyrus L Middle occipital gyrus L Cuneus L Calcarine fissure OUTSIDE | 93 51 40 22 1 | 207 | 0.001 |
| 8 | R Cerebellum 6 OUTSIDE R Crus Cerebellum 1 R Crus Cerebellum 2 R Fusiform gyrus R Cerebellum 4-5 R Cerebellum 7b | 48 35 26 14 10 5 2 | 140 | 0.012 |

*Group level contrast showing greater neural engagement when mothers are viewing their own infants compared to viewing others’ infants. Significance threshold T(23) = 3.48; Cluster extent threshold = 140 voxels.*

**[InfOther > InfOwn](#Section_1_Table)**

| **Cluster** | **Regions** | **Number of voxels in region** | **Cluster size** | **FWE-corrected p-value** |
| --- | --- | --- | --- | --- |
| 1 | L Superior temporal gyrus L Middle temporal gyrus L Heschl gyrus | 226 19 2 | 247 | < 0.001 |
| 2 | R Superior temporal gyrus R Middle temporal gyrus | 183 8 | 191 | 0.002 |

*Group level contrast showing greater neural engagement when mothers are viewing others’ infants compared to viewing their own infants. Significance threshold T(23) = 3.48; Cluster extent threshold = 191 voxels.*

**[InfPos > InfNeg](#Section_1_Table)**

| **Cluster** | **Regions** | **Number of voxels in region** | **Cluster size** | **FWE-corrected p-value** |
| --- | --- | --- | --- | --- |
| 1 | R Lingual gyrus R Calcarine fissure | 139 3 | 142 | 0.009 |

*Group level contrast showing greater neural engagement when mothers view infants engaged in a positive emotion task compared to viewing them in a negative emotion task. Significance threshold T(23) = 3.48; Cluster extent threshold = 142 voxels.*

**[InfNeg > InfPos](#Section_1_Table)**

| **Cluster** | **Regions** | **Number of voxels in region** | **Cluster size** | **FWE-corrected p-value** |
| --- | --- | --- | --- | --- |
| 1 | L Insula OUTSIDE L Superior temporal gyrus L Rolandic operculum L Putamen L Heschl gyrus L IFG, opercular part L IFG, orbital part L Postcentral gyrus L Middle temporal gyrus | 440 190 144 129 105 63 14 4 3 1 | 1093 | < 0.001 |
| 2 | R Rolandic operculum R Insula R Superior temporal gyrus R Heschl gyrus R IFG, opercular part OUTSIDE R Precentral gyrus R Temporal pole: superior temporal gyrus R Putamen | 312 221 193 50 39 26 5 4  1 | 851 | < 0.001 |
| 3 | R Amygdala OUTSIDE R Putamen R Hippocampus R Temporal pole: superior temporal gyrus | 48 35 18 3 2 | 106 | 0.04 |

*Group level contrast showing greater neural engagement when mothers view infants engaged in a negative emotion task compared to viewing them in a positive emotion task. Significance threshold T(23) = 3.48; Cluster extent threshold = 106 voxels.*

**[Section II.](#Table_of_Contents)** [Results of Regression against Psychological Variables](#Table_of_Contents)

This section reports the regression analysis of main contrasts against markers of anxiety and depression discussed in the accompanying manuscript. See Section IV for a summary of all other models tested. Continuous behavioral scores (CESD and BAI) are mean centered while categorical variables are dummy coded with no centering (Dep Hx, Dep Dx, Anx Hx, and Anx Dx), and an intercept is included in all models for interpretability. F-Tests were conducted for all regression coefficients tests; however, only t-tests for directionality are reported here. All regression coefficients were tested for positive and negative effects. If a variable/direction is not reported, this implies there were no significant results for this test. For ease of conceptualization, the regression model used is listed before each set of results. Primary voxel-wise correction was used at p < .0001 (unc.) for cluster definition; Cluster-level correction conducted at p < .05 (FWE). All clusters labelled using the AAL^[1]^ atlas with labels listed in decreasing order of volume coverage of cluster.

Although not described in the main manuscript, each model was also tested against (InfPos > InfNeg) to understand the role of infant emotional cues more broadly (see Section IV).

***Note on Use of Hierarchical Modeling* —** Although a linear (intercept and slope) model could be fit to the CESD scores, neural response effects were found to involve symptom levels (intercepts) and not slopes, and BAI scores did not support a linear model. Therefore, intercept estimates representing mothers’ mean depression (CESD[T1-4]) and anxiety (BAI[T1-4]) symptoms were used in subsequent regression analysis. See Table 2 in the main manuscript for descriptives including observed scores at the T1 and T4 assessments.

Below is a summary of all regression models tested.

| **Infant Viewing Task** | |
| --- | --- |
| **InfOwnPos > InfOwnNeg** | |
| [(1) CESD[T1-4]](#Section_2_A_Model_1)  [(2) BAI[T1-4]](#Section_2_A_Model_2) | [(3) Dep Hx + Dep Dx](#Section_2_A_Model_3)  [(4) Anx Hx + Anx Dx](#Section_2_A_Model_4) |
| *Key:*  (Continuous)  *CESD[T1-4] = estimated average CESD (depression) symptom scores across all 4 post-natal assessments; BAI[T1-4] = estimated average BAI (anxiety) symptom scores*  (Categorical)  *Dep Hx = history of depressive disorder; Dep Dx = current depressive disorder; Anx Hx = history of anxiety disorder; Anx Dx = current anxiety disorder* | |

*Abbreviation Key:*

*SFG = Superior frontal gyrus; IP = Inferior parietal; IFG = Inferior frontal gyrus; MFG = Middle frontal gyrus; MTG = Middle temporal gyrus; STG = Superior temporal gyrus; OUTSIDE = not defined in the AAL atlas.*

**[Section II.A.](#Table_of_Contents)** [Regression against (InfOwnPos > InfOwnNeg)](#Table_of_Contents)

*------------------------------------------------------------------------------------------------------------------------------------------*

***[Model 1:](#Section_2_Table)*** $\left( InfOwnPos > InfOwnNeg \right)= \beta_{0}+ \beta_{1}\left( CESD[T1\text{-}4] \right)$

*------------------------------------------------------------------------------------------------------------------------------------------*

**( InfOwnPos > InfOwnNeg ) – positive association with CESD[T1-4]**

| **Cluster** | **Regions** | **Number of voxels in region** | **Cluster size** | **FWE-corrected p-value** |
| --- | --- | --- | --- | --- |
| 1 | R Cerebellum 6 OUTSIDE R Cerebellum 8 Vermis 7 Vermis 6 R Crus Cerebellum 1 R Cerebellum 9 R Cerebellum 4-5 R Crus Cerebellum 2 L Cerebellum 6 L Crus Cerebellum 1 | 203 119 98 33 30 23 14 8 5 3 1 | 537 | < 0.001 |

*Group level contrast showing more positive differences between the estimated contributions to neural activity of mothers viewing their infants engaged in positive emotion tasks subtracted from viewing them in negative emotion tasks* *as postnatal depression symptoms increase. Significance threshold T(22) = 3.50; Cluster extent threshold = 537 voxels.*

**( InfOwnPos > InfOwnNeg ) – negative association with CESD[T1-4]**

| **Cluster** | **Regions** | **Number of voxels in region** | **Cluster size** | **FWE-corrected p-value** |
| --- | --- | --- | --- | --- |
| 1 | L Paracentral lobule R SFG, dorsolateral L Postcentral gyrus R Precentral gyrus R Supplementary motor area L Superior parietal gyrus R Paracentral lobule L Supplementary motor area L Precuneus L Precentral gyrus L SFG, dorsolateral OUTSIDE R Postcentral gyrus R Middle frontal gyrus | 272 176 174 132 114 80 77 64 62 58 55 44 42 13 | 1363 | < 0.001 |
| 2 | R Postcentral gyrus OUTSIDE R IP, supramarginal and angular gyri R Superior parietal gyrus R Angular gyrus R Precentral gyrus R Supramarginal gyrus | 329 200  152  141 109 18 1 | 950 | < 0.001 |
| 3 | OUTSIDE L IP, supramarginal and angular gyri L Postcentral gyrus L Paracentral lobule L Supplementary motor area | 295 29  17 2 2 | 345 | < 0.001 |
| 4 | L Inferior temporal gyrus OUTSIDE L Temporal pole: STG L Fusiform gyrus L Parahippocampal gyrus L Amygdala L Middle temporal gyrus L IFG, orbital part | 53 37 24 22 14 8 5 5 | 168 | 0.004 |
| 5 | R SFG, dorsolateral OUTSIDE R Middle frontal gyrus | 120 19 12 | 151 | 0.006 |
| 6 | R SFG, dorsolateral R SFG, medial | 64 60 | 124 | 0.017 |

*Group level contrast showing more negative differences between the estimated contributions to neural activity of mothers viewing their infants engaged in positive emotion tasks subtracted from viewing them in negative emotion tasks* *as postnatal depression symptoms increase. Significance threshold T(22) = 3.50; Cluster extent threshold = 124 voxels.*

**( InfOwnPos > InfOwnNeg ) – negative association with Model Intercept**

| **Cluster** | **Regions** | **Number of voxels in region** | **Cluster size** | **FWE-corrected p-value** |
| --- | --- | --- | --- | --- |
| 1 | R Insula OUTSIDE R Rolandic operculum R Putamen R IFG, opercular part | 76 32 12 5 2 | 127 | 0.015 |

*Group level contrast showing regions in the brain where having postnatal anxiety symptoms less than the group average is predictive of the difference between mothers viewing their infants engaged in positive emotion tasks compared to negative emotion tasks. Significance threshold T(22) = 3.50; Cluster extent threshold = 127 voxels.*

*------------------------------------------------------------------------------------------------------------------------------------------*

***[Model 2:](#Section_2_Table)*** $\left( InfOwnPos > InfOwnNeg \right)= \beta_{0}+ \beta_{1}\left( BAI[T1\text{-}4] \right)$

*------------------------------------------------------------------------------------------------------------------------------------------*

**( InfOwnPos > InfOwnNeg ) – negative association with BAI[T1-4]**

| **Cluster** | **Regions** | **Number of voxels in region** | **Cluster size** | **FWE-corrected p-value** |
| --- | --- | --- | --- | --- |
| 1 | OUTSIDE R Postcentral gyrus L Postcentral gyrus R SFG, dorsolateral L IP, supramarginal and angular gyri L Paracentral lobule R Supplementary motor area L Precentral gyrus R Superior parietal gyrus R Precentral gyrus L Superior parietal gyrus L Precuneus R IP, but supramarginal and angular gyri R Middle frontal gyrus R Precuneus L Supplementary motor area R Paracentral lobule R Angular gyrus R Median cingulate and paracingulate gyri R Middle occipital gyrus L Median cingulate and paracingulate gyri L SFG, dorsolateral R Superior occipital gyrus L Middle occipital gyrus L Angular gyrus R Supramarginal gyrus R Posterior cingulate gyrus R SFG, medial L Superior occipital gyrus L Middle frontal gyrus L Posterior cingulate gyrus L Supramarginal gyrus R Cuneus L Anterior cingulate and paracingulate gyri R Superior temporal gyrus R IFG, opercular part | 2823 1082 1057 995 886  867 849 669 659 633 604 589 536  508 485 466 453 439  404  367 350  318 274 224 140 103 64 62 51 36 18 9  9 9  4 1 | 17043 | < 0.001 |
| 2 | R Middle frontal gyrus R SFG, dorsolateral R IFG, triangular part R SFG, medial OUTSIDE R SFG, orbital part | 536 204 167 60 46 4 | 1017 | < 0.001 |
| 3 | L Middle temporal gyrus L Fusiform gyrus L Parahippocampal gyrus OUTSIDE L Inferior temporal gyrus L Hippocampus | 63 47 36 26 19 6 | 197 | < 0.001 |
| 4 | L Crus Cerebellum 2 L Cerebellum 8 L Cerebellum 7b L Crus Cerebellum 1 | 110 19 18 17 | 164 | 0.002 |
| 5 | OUTSIDE R IFG, orbital part R SFG, orbital part R Caudate nucleus R Gyrus rectus R MFG, orbital part | 65 57 18 3 3 1 | 147 | 0.005 |
| 6 | L Middle occipital gyrus L Superior occipital gyrus L Cuneus | 107 21 1 | 129 | 0.009 |
| 7 | R Parahippocampal gyrus R Fusiform gyrus R Hippocampus OUTSIDE | 81 19 12 5 | 117 | 0.015 |
| 8 | OUTSIDE L Middle frontal gyrus L IFG, triangular part L Insula | 55 38 12 1 | 106 | 0.024 |
| 9 | OUTSIDE R Temporal pole: MTG R Inferior temporal gyrus R Middle temporal gyrus R Temporal pole: STG | 43 31 11 11 3 | 99 | 0.033 |

*Group level contrast showing more negative differences between the estimated contributions to neural activity of mothers viewing their infants engaged in positive emotion tasks subtracted from viewing them in negative emotion tasks* *as postnatal anxiety symptoms increase. Significance threshold T(22) = 3.50; Cluster extent threshold = 99 voxels.*

**( InfOwnPos > InfOwnNeg ) – negative association with Model Intercept**

| **Cluster** | **Regions** | **Number of voxels in region** | **Cluster size** | **FWE-corrected p-value** |
| --- | --- | --- | --- | --- |
| 1 | R Insula OUTSIDE R Rolandic operculum R Putamen R IFG, opercular part | 75 25 12 3 2 | 117 | 0.015 |

*Group level contrast showing regions in the brain where having postnatal depression symptoms less than the group average is predictive of the difference between mothers viewing their infants engaged in positive emotion tasks compared to negative emotion tasks. Significance threshold T(22) = 3.50; Cluster extent threshold = 117 voxels.*

*------------------------------------------------------------------------------------------------------------------------------------------*

***[Model 3:](#Section_2_Table)*** $\left( InfOwnPos > InfOwnNeg \right)= \beta_{0}+ \beta_{1}\left( Dep Hx \right)$

$+ \beta_{2}\left( Dep Dx \right)$

*------------------------------------------------------------------------------------------------------------------------------------------*

**( InfOwnPos > InfOwnNeg ) – negative association with Dep Dx**

| **Cluster** | **Regions** | **Number of voxels in region** | **Cluster size** | **FWE-corrected p-value** |
| --- | --- | --- | --- | --- |
| 1 | R SFG, dorsolateral R Postcentral gyrus R Angular gyrus R Middle frontal gyrus R Precentral gyrus R IP, supramarginal and angular gyri R Supplementary motor area OUTSIDE R Superior parietal gyrus R SFG, medial L SFG, medial R Precuneus L SFG, dorsolateral R Superior occipital gyrus R Supramarginal gyrus R Middle occipital gyrus R Paracentral lobule R Median cingulate and paracingulate gyri R Middle temporal gyrus L Middle frontal gyrus | 990 504 425 392 335  300  295 236 199 122 117 77 51 38 35 30 20 11  7 1 | 4185 | < 0.001 |
| 2 | L Postcentral gyrus L Precentral gyrus OUTSIDE L IP, supramarginal and angular gyri L Superior parietal gyrus L Precuneus L SFG, dorsolateral L Middle frontal gyrus L Paracentral lobule L Supplementary motor area L Median cingulate and paracingulate gyri L Angular gyrus | 616 489 448 298  284 207 107 23 13 9 8  1 | 2503 | < 0.001 |
| 3 | R Middle frontal gyrus R IFG, triangular part R SFG, dorsolateral R SFG, medial R IFG, opercular part OUTSIDE R Precentral gyrus R SFG, medial orbital R IFG, orbital part R MFG, orbital part R Postcentral gyrus | 577 228 185 97 92 75 68 26 4 2 1 | 1355 | < 0.001 |
| 4 | L Middle temporal gyrus OUTSIDE L Inferior temporal gyrus L Fusiform gyrus L Hippocampus | 106 86 64 44 9 | 309 | < 0.001 |
| 5 | L IFG, triangular part OUTSIDE L IFG, opercular part L Insula L Middle frontal gyrus | 280 8 4 1 1 | 294 | < 0.001 |
| 6 | L Inferior temporal gyrus OUTSIDE L Middle temporal gyrus L Fusiform gyrus | 92 22 10 6 | 130 | 0.011 |
| 7 | OUTSIDE R Postcentral gyrus R Supramarginal gyrus R Precentral gyrus | 59 51 3 1 | 114 | 0.021 |
| 8 | L Angular gyrus L Middle occipital gyrus L IP, supramarginal and angular gyri OUTSIDE | 56 41 11  5 | 113 | 0.021 |
| 9 | L SFG, medial L SFG, dorsolateral L Middle frontal gyrus L SFG, medial orbital | 49 45 2 1 | 97 | 0.042 |

*Group level contrast showing areas of more negative difference in neural demands when mothers view infants in a positive emotion task compared to negative when there is a current diagnosis of depression. Significance threshold T(21) = 3.53; Cluster extent threshold = 97 voxels.*

*------------------------------------------------------------------------------------------------------------------------------------------*

***[Model 4:](#Section_2_Table)*** $\left( InfOwnPos > InfOwnNeg \right)= \beta_{0}+ \beta_{1}\left( Anx Hx \right)+ \beta_{2}\left( Anx Dx \right)$

*------------------------------------------------------------------------------------------------------------------------------------------*

**( InfOwnPos > InfOwnNeg ) – positive association with Anx Hx**

| **Cluster** | **Regions** | **Number of voxels in region** | **Cluster size** | **FWE-corrected p-value** |
| --- | --- | --- | --- | --- |
| 1 | R Cerebellum 6 OUTSIDE R Cerebellum 8 | 101 33 5 | 139 | 0.007 |

*Group level contrast showing areas of more positive difference in neural demands when mothers view infants in a positive emotion task compared to negative when there is a history of anxiety. Significance threshold T(21) = 3.53; Cluster extent threshold = 139 voxels.*

**( InfOwnPos > InfOwnNeg ) – negative association with Anx Hx**

| **Cluster** | **Regions** | **Number of voxels in region** | **Cluster size** | **FWE-corrected p-value** |
| --- | --- | --- | --- | --- |
| 1 | R Middle frontal gyrus OUTSIDE R IFG, triangular part R SFG, dorsolateral | 159 16 4 2 | 181 | 0.002 |
| Clusters Spanning a Single Region | OUTSIDE |  | 106 | 0.028 |

*Group level contrast showing areas of more negative difference in neural demands when mothers view infants in a positive emotion task compared to negative when there is a history of anxiety. Significance threshold T(21) = 3.53; Cluster extent threshold = 106 voxels.*

**( InfOwnPos > InfOwnNeg ) – negative association with Anx Dx**

| **Cluster** | **Regions** | **Number of voxels in region** | **Cluster size** | **FWE-corrected p-value** |
| --- | --- | --- | --- | --- |
| 1 | R Precentral gyrus R Middle frontal gyrus OUTSIDE | 128 43 24 | 195 | 0.001 |
| 2 | L Precentral gyrus L IFG, opercular part L IFG, triangular part L Middle frontal gyrus L Postcentral gyrus | 100 29 24 5 2 | 160 | 0.003 |
| 3 | OUTSIDE R Angular gyrus R Middle temporal gyrus R Superior temporal gyrus | 43 41 40 18 | 142 | 0.007 |
| 4 | L Middle occipital gyrus OUTSIDE | 125 10 | 135 | 0.009 |
| 5 | R IFG, opercular part R Precentral gyrus | 53 49 | 102 | 0.033 |

*Group level contrast showing areas of more negative difference in neural demands when mothers view infants in a positive emotion task compared to negative when there is a current diagnosis of anxiety. Significance threshold T(21) = 3.53; Cluster extent threshold = 102 voxels.*

**[Section III.](#Table_of_Contents)** [Participant Demographics](#Table_of_Contents)

The following section contains further information characterizing the reported demographics of the study participants.

**[Section III.A.](#Table_of_Contents)** [Recruitment Strategy](#Table_of_Contents)

The initial study began by approaching women who presented with elevated depression symptoms but no other psychological disorder, who were MR eligible, and met other inclusion criteria for the larger study as reported in the methods section of the main manuscript (“could speak English, had an infant younger than 12 weeks-old, and planned to remain in the region until the infant was 18 months old”). Following consistently low enrollment rates due to low numbers of eligible women, the sampling criteria was changed to recruit anyone from the larger study who was willing to participate, regardless of levels of depression or comorbidity, so long as all other inclusion criteria were met. All women who were approached agreed to participate in the imaging portion of the study. The original intent was to recruit 30 participants, but 25 participants were reached by the time the overall study was ready to be discontinued.

**[Section III.B.](#Table_of_Contents)** [Dropout Comparisons](#Table_of_Contents)

Although all (*n=25*) mothers completed the first (3-month) and second (6-month) assessments, 19 mothers returned for the third (12-month) and 16 mothers returned for the fourth (18-month) assessments. One mother did not complete the third assessment, but returned for the fourth. Group means/contingency comparisons were conducted between those that completed all assessments and those that did not. Values were calculated for all demographics reported in the main manuscript. Additionally, anxiety and depression measures taken at the first assessment and reported in the main manuscript were tested for differences. For continuous data, an unpaired 2-sample, 2-tail Welch approximation t-test was employed, and a 2-sided Fischer’s exact test was used for categorical outcomes. After applying Bonferonni correction for multiple comparisons (*p_crit_=* *0.00357*), no measure was significantly different between completers and dropouts.

| **Continuous Measures** | | | | |
| --- | --- | --- | --- | --- |
| **Demographic** | **Mean of Completers** | **Mean of Dropouts** | **Statistic** | **p-value** |
| Age | 27.86 | 25.0 | t(21.26) = 1.92 | 0.069 |
| CESD-T1raw | 6.36 | 11.20 | t(13.14) = -1.13 | 0.278 |
| BAI-T1raw | 6.59 | 8.93 | t(19.46) = -0.77 | 0.448 |

| **Categorical Measures** | | | | |  |
| --- | --- | --- | --- | --- | --- |
| **Demographic** | **p-value** |  | **Demographic** | **p-value** | |
| Annual Income | 0.267 |  | Child Birth Order | 0.3294 | |
| Primary Racial Identification | 0.037 |  | DepHx | 1.00 | |
| Education Completed | 0.601 |  | AnxHx | 0.163 | |
| Partner Status | 0.568 |  | DepDx | 1.00 | |
| Mode of Delivery | 0.408 |  | AnxDx | 0.272 | |
| Breastfeeding* | 0.550 |  |  |  | |

**Taken at time of first assessment (3-month postpartum).*

*CESD-T1raw are observed Center for Epidemiologic Studies Depression Scale (CESD)^[2]^ scores for the 3-month assessment. Similarly, the BAI-T1raw observed Beck Anxiety Inventory (BAI)^[3]^ scores.* *Dep Hx = history of depressive disorder based on the Structured Clinical Interview for DSM-IV(SCID)^[4]^; Dep Dx = current depressive disorder; Anx Hx = anxiety disorder history; Anx Dx = current anxiety disorder*

**[Section III.C.](#Table_of_Contents)** [Contingency Tables of History and Diagnosis Variables](#Table_of_Contents)

Below is a contingency describing the co-occurrence of past anxiety and depressive episodes and current anxiety and depressive disorder for participants included in the reported analysis. Rating were made by trained graduate students using the Structured Clinical Interview for DSM-IV (SCID)^[4]^ during the first assessment (T1), the assessment when brain imaging occurred.

|  | ***Dep-Hx*** | ***Dep-Dx*** | ***Dep-Hx&Dx*** | ***No-Dep*** |
| --- | --- | --- | --- | --- |
| ***Anx-Hx*** | *1* | *1* |  |  |
| ***Anx-Dx*** | *1* | *1* |  | *2** |
| ***Anx-Hx&Dx*** |  |  |  |  |
| ***No-Anx*** | *2* |  |  | *16* |

** The participant excluded for excessive head movement did not exhibit evidence for any depressive disorder, but did meet criteria for a current anxiety disorder.*

Additionally, 4 women included in the final analysis also met criteria for post-traumatic stress disorder (PTSD). Exposure to traumatic stress may manifest as depression and/or anxiety and provide a potential source of common variance that is related to maternal neural response to their infants. Although it is beyond the scope of the current study to tease apart the role of trauma in influencing the relationship between brain response and depression/anxiety symptom development, we include a contingency table of current PTSD diagnosis and current depression/anxiety disorder diagnosis to allow the reader to evaluate the joint distribution of these disorders.

|  | ***Anx*** | | ***No-Anx*** | |
| --- | --- | --- | --- | --- |
|  | ***Dep-Dx*** | ***No-Dep*** | ***Dep-Dx*** | ***No-Dep*** |
| ***PTSD*** | *1* | *1* | *1* | *1* |
| ***No-PTSD*** | *0* | *2** | *0* | *18* |

** The participant excluded for excessive head movement did not exhibit evidence for PTSD or any current depressive disorder, but did meet criteria for a current anxiety disorder.*

**[Section IV.](#Table_of_Contents)** Post-Scan Rating of Infant Videos

Immediately after the scanning session on the mothers’ first visit (T1), they were replayed all videos shown and asked to rate the valence and intensity (arousal) of the videos both for their infants and for themselves while viewing the videos. The following questions were asked for each video:

**Emotions of infant**

**Own Infant**

1. In this video clip, how positive or negative is your baby's emotion?-Negative-Positive
2. In this video clip, how intense is your baby's emotion?-Intensity

**Other’s Infant**

1. In this video clip, how positive or negative is this baby's emotion?-Negative-Positive
2. In this video clip, how intense is this baby's emotion?-Intensity

**Mother’s emotions while viewing infant**

1. While watching this video clip, how positive or negative is your emotion?-Negative-Positive
2. While watching this video clip, how intense is your emotion?-Intensity

Negative-Positive (emotional valence) questions were all rated on a scale of -100 (*most negative*) to +100 (*most positive*). Intensity (emotional arousal) question were all rated on a scale of 0 (*no intensity*) to 100 (*most intense*).

The table below provides descriptive statistics of these self-report measures for own-infant positive (peek-a-boo) and negative (arm-restraint) videos used in the current analysis. Note that not all mothers answered all questions and thus could not be included in this summary.

| **Own Infant Negative Videos** | | | | |
| --- | --- | --- | --- | --- |
|  | ***Mother’s Emotions*** | | ***Perception of Infant Emotions*** | |
|  | *Valence (n = 23)* | *Intensity (n = 21)* | *Valence (n = 22)* | *Intensity (n = 24)* |
| **[Min, Max]** | [-72, 100] | [2, 100] | [-100, 100] | [3, 100] |
| **Mean [95% CI]** | 22.39 (50.72)  [0.46, 44.32] | 50.90 (33.84)  [35.50, 66.31] | 3.18 (55.69)  [-21.51, 27.87] | 47.71 (35.18)  [32.85, 62.56] |

| **Own Infant Positive Videos** | | | | |
| --- | --- | --- | --- | --- |
|  | ***Mother’s Emotions*** | | ***Perception of Infant Emotions*** | |
|  | *Valence (n = 24)* | *Intensity (n = 22)* | *Valence (n = 24)* | *Intensity (n = 22)* |
| **[Min, Max]** | [0, 100] | [17, 100] | [-25, 100] | [13, 100] |
| **Mean (SD)**  **[95% CI]** | 85.88 (27.18)  [74.40, 97.35] | 66.23 (27.10)  [54.21, 78.24] | 69.4 (37.4)  [53.62, 85.21] | 54.45 (25.99)  [32.85, 62.56] |

**[Section IV.A.](#Table_of_Contents)** Correlations among Post-Scan Ratings

Pearson’s correlations were calculated to get a sense of associations among post-scan ratings of infant videos were. Statistical tests were Bonferonni corrected for all tests performed in this section (32 tests in total). In summary, mothers tended to rate their own and their infant’s valence similarly so that when they reported that their valence or intensity was high/low, they were also likely to report the same in their infant’s emotions.

|  |  |  | **Mother’s Emotions** | | | |
| --- | --- | --- | --- | --- | --- | --- |
|  |  |  | **Positive Videos** | | **Negative Videos** | |
|  |  |  | **Valence** | **Intensity** | **Valence** | **Intensity** |
| **Perception of Infant Emotions** | **Positive Videos** | **Valence** | **0.69^†^**  *n = 24* | 0.21  *n = 22* | -0.10  *n = 23* | 0.44*  *n = 21* |
|  |  | **Intensity** | 0.33  *n = 22* | **0.66^†^**  *n = 22* | 0.37  *n = 21* | 0.53*  *n = 20* |
|  | **Negative Videos** | **Valence** | -0.04  *n = 22* | 0.35  *n = 21* | **0.73^†^**  *n = 22* | 0.28  *n = 19* |
|  |  | **Intensity** | 0.24  *n = 24* | 0.41  *n = 22* | 0.11  *n = 23* | **0.93^†^**  *n = 21* |

Not all mother answered all questions. Only complete observation pairs were used to calculate associations. * (p < .05); ** (p < .01); ^†^ (p < .001) uncorrected. Those in bold also passed Bonferroni correction at p < .05 (FWE).

|  |  |  | **Mother’s Emotions** | | | |
| --- | --- | --- | --- | --- | --- | --- |
|  |  |  | **Positive Videos** | | **Negative Videos** | |
|  |  |  | **Valence** | **Intensity** | **Valence** | **Intensity** |
| **Mothers Emotions** | **Positive Videos** | **Valence** |  |  |  |  |
|  |  | **Intensity** | 0.38  *n = 22* |  |  |  |
|  | **Negative Videos** | **Valence** | 0.10  *n = 23* | 0.39  *n = 21* |  |  |
|  |  | **Intensity** | 0.33  *n = 21* | 0.49  *n = 20* | 0.22  *n = 20* |  |

Not all mother answered all questions. Only complete observation pairs were used to calculate associations. * (p < .05); ** (p < .01); ^†^ (p < .001) No correlations passed significance at p < .05

|  |  |  | **Perception of Infant Emotions** | | | |
| --- | --- | --- | --- | --- | --- | --- |
|  |  |  | **Positive Videos** | | **Negative Videos** | |
|  |  |  | **Valence** | **Intensity** | **Valence** | **Intensity** |
| **Perception of Infant Emotions** | **Positive Videos** | **Valence** |  |  |  |  |
|  |  | **Intensity** | 0.36  *n = 22* |  |  |  |
|  | **Negative Videos** | **Valence** | -0.12  *n = 22* | 0.20  *n = 21* |  |  |
|  |  | **Intensity** | 0.38  *n = 24* | 0.63**  *n = 22* | 0.04  *n = 22* |  |

Not all mother answered all questions. Only complete observation pairs were used to calculate associations. * (p < .05); ** (p < .01); ^†^ (p < .001). No correlations passed significance after Bonferroni correction at p < .05 (FWE).

**[Section IV.B.](#Table_of_Contents)** Association of Post-Scan Ratings with Psychopathology

To assess whether markers of depression or anxiety were associated with mother’s self-reported ratings of their and their infant’s emotions (valence and arousal/intensity), cross-measure correlations were calculated and are reported below.

|  |  | **Mother’s Emotions** | | | |
| --- | --- | --- | --- | --- | --- |
|  |  | **Positive Videos** | | **Negative Videos** | |
|  |  | **Valence**  **(n = 24)** | **Intensity**  **(n = 22)** | **Valence**  **(n = 23)** | **Intensity**  **(n = 21)** |
| **Psychological Measures** | **Dep Hx** | 0.12 | -0.23 | 0.03 | -0.08 |
|  | **Anx Hx** | -0.27 | -0.32 | -0.33 | -0.13 |
|  | **Dep Dx** | 0.08 | -0.07 | -0.35 | 0.34 |
|  | **Anx Hx** | 0.11 | 0.07 | 0.04 | -0.15 |
|  | **CESD[T1-4]** | -0.06 ^A^ | -0.36 ^A^ | -0.23 ^A^ | 0.2 ^A^ |
|  | **BAI[T1-4]** | -0.12^A^ | -0.47* ^A^ | -0.55** ^A^ | 0.24 ^A^ |

Association Matrix between markers of psychological distress and mothers’ post-task perceptions of their infants’ emotions in videos presented during the scan. Dep Hx = history of depressive disorder based on criteria gathered from the Structured Clinical Interview for DSM-5 (SCID); Dep Dx = current depressive disorder; Anx Hx = anxiety disorder history; Anx Dx = current anxiety disorder; CESD[T1-4] = estimated mean CESD score across all assessments time point (i.e. Estimated Future Depression Symptom Severity); BAI[T1-4] = estimated mean BAI score (i.e. Estimated Future Anxiety Severity). Although psychological measures were collected for all mothers not all mother answered all questions. Only complete observation pairs were used to calculate associations. (A) Denotes Pearson’s correlation between a continuous-continuous pair. All other associations reported (categorical-categorical or categorical-continuous pairs) are reported using Spearman’s rank correlation. * (p < .05); ** (p < .01). Note that both significant associations did not pass Bonferroni correction for multiple comparisons at p < .05 (FWE).

|  |  | **Perception of Infant Emotions** | | | |
| --- | --- | --- | --- | --- | --- |
|  |  | **Positive Videos** | | **Negative Videos** | |
|  |  | **Valence**  **(n = 24)** | **Intensity**  **(n = 22)** | **Valence**  **(n = 22)** | **Intensity**  **(n = 24)** |
| **Psychological Measures** | **Dep Hx** | 0.27 | -0.2 | 0.13 | -0.22 |
|  | **Anx Hx** | -0.06 | -0.09 | -0.25 | -0.09 |
|  | **Dep Dx** | 0.23 | 0.26 | -0.33 | 0.34 |
|  | **Anx Hx** | 0.13 | 0.02 | 0.2 | -0.15 |
|  | **CESD[T1-4]** | 0.08 ^A^ | 0.03 ^A^ | -0.15 ^A^ | 0.13 ^A^ |
|  | **BAI[T1-4]** | 0.08 ^A^ | -0.13 ^A^ | -0.38 ^A^ | 0.13 ^A^ |

Association Matrix between markers of psychological distress and mothers’ post-task perceptions of their infants’ emotions in videos presented during the scan. Dep Hx = history of depressive disorder based on criteria gathered from the Structured Clinical Interview for DSM-5 (SCID); Dep Dx = current depressive disorder; Anx Hx = anxiety disorder history; Anx Dx = current anxiety disorder; CESD[T1-4] = estimated mean CESD score across all assessments time point (i.e. Estimated Future Depression Symptom Severity); BAI[T1-4] = estimated mean BAI score (i.e. Estimated Future Anxiety Severity). Although psychological measures were collected for all mothers not all mother answered all questions. Only complete observation pairs were used to calculate associations. (A) Denotes Pearson’s correlation between a continuous-continuous pair. All other associations reported (categorical-categorical or categorical-continuous pairs) are reported using Spearman’s rank correlation. No significant associations were found.

**[Section V.](#Table_of_Contents)** Models not Reported in Main Manuscript

Beyond the regression models testing primary study hypotheses reported in the main manuscript, several models examining relations between brain response and additional measures of maternal stress/distress collected for the larger study were explored. In an effort to adhere to transparent reporting, these results are summarized in this section. Similar to the contrasts in Section II, all models were tested for significance with a whole-brain voxel-wise analysis, cluster-corrected with a p < 0.001 primary (voxel-level) threshold and FWE-corrected p < .05 at cluster-level, using implicit masking only. F-Tests were conducted for all regression coefficients tests; however, only t-tests for directionality are reported here. All regression coefficients were tested for positive and negative effects. If a variable/direction is not reported, this implies there were no significant results for this test. All clusters are labelled using the AAL^[1]^ atlas with labels listed in decreasing order of volume coverage of cluster.

To explore the role of chronicity of psychopathology, “dosage” models were constructed for depression and anxiety in which participants with no past or current psychopathology received a 0 score, either history or current diagnosis a 1 score, and both history and current a 2 score (no participants were scored 2). A similar dosage model combined depression and anxiety to examine broader effects of total affective psychopathology. Participants who were missing the relevant observation (not all mothers returned for the time 4 assessment, precluding complete observation pairs for the raw scores of continuous measures; although all time 1 observations were complete) were dropped from those models.

In addition to the CESD measure of depression levels at the time of assessment, which is reported in the main manuscript, the Edinburgh Postnatal Depression Scale^[5]^ retrospectively evaluating depression symptoms in the first month postpartum was administered to mothers during the first assessment time. Retrospective reports of anxiety and depression symptoms during pregnancy were also gathered via a subset of questions taken from existing scales. See [6] for a full description of these measures. Salivary cortisol preceding and following tasks designed to produce stress in mothers and infants were gathered during data collection for the larger study at 6, 12, and 18 months postnatal—details of this paradigm are reported in [6].

A model of exposure to stressful events was also estimated in order to understand the relative importance of chronic vs acute stress in differential brain response to mother’s infants. This model used the Life Experiences Survey (LE)^[7]^ and the Parenting Stress Scale (PS)^[8]^ as markers of lifetime stressor exposure and recent exposure related to the transition to motherhood, respectively. Scores taken at the first assessment when brain imaging occurred were used in this model.

Our primary measures reported were the BAI and CESD, which were considered the most reliable measures of maternal psychopathology across the postnatal period. In particular, these measures were not retrospective and used fully validated measures of anxiety and depression. We did however wish to explore the role of earlier (pregnancy-early postpartum) anxiety and depression on maternal response to infants, understanding that these measures are inherently subject to recall bias. We also chose to explore the associations with cortisol responsivity due its known association with maternal risk for psychopathology. Many of these models were run in the larger contrasts of both a mother’s own and unknown infants both to understand how parental brain networks may be exhibit hyper or hypo-reactivity to more general infant cues and for the statistical benefit of a larger number of observed data points. We consider these tests exploratory and hope that they may inform further hypothesis-driven research questions.

All continuous behavioral observations (Cortisol, Retrospective Pregnancy Depression/Anxiety, EPDS, CESD and BAI), both observed (raw scores) and predicted (intercepts, linear terms, and slopes from the hierarchical linear model discussed in the main manuscript) are mean centered. Categorical variables (Dep Hx, Dep Dx, Anx Hx, Anx Dx, Dep/Anx Hx, Dep/Anx Dx, Dep “Dose”, and Anx “Dose”) include an intercept but are not mean centered. All models include an intercept term. Please also note that none of these models incorporated regularization. Collinearity was assessed for all predictors and is reported below in [Section V.A](#Section_5_A). We encourage the reader to interpret models using multiple predictors with caution.

Below is a summary of all regression models tested.

| **Infant Viewing Task** | |
| --- | --- |
| **InfOwnPos > InfOwnNeg** | |
| [(5) CESD[T4-slope] + CESD[T4-intercept]](#Section_5_B_Model_5)  [(6) BAI[T4-intercept]](#Section_5_B_Model_6)  [(7) CESD[T1-4-intercept] + BAI[T1-4-intercept]](#Section_5_B_Model_7)  [(8) Dep/Anx Hx](#Section_5_B_Model_8)  [(9) Dep/Anx Dx](#Section_5_B_Model_9) | [(10) Dep Dose](#Section_5_Table)  [(11) Anx Dose](#Section_5_B_Model_11)  [(12) Cortisol[T4-linear-term] + Cortisol[T4-intercept]](#Section_5_B_Model_12)  [(13) LE[T1-raw] + PS[T1-raw]](#Section_5_B_Model_13) |
| **InfPos > InfNeg** | |
| [(14) CESD[T4-slope] + CESD[T4-intercept]](#Section_5_C_Model_14)  [(15) CESD[T1-4-intercept]](#Section_5_C_Model_15)  [(16) BAI[T4-intercept]](#Section_5_C_Model_16)  [(17) BAI[T1-4-intercept]](#Section_5_C_Model_17)  [(18) CESD[T4-intercept] + BAI[T4-intercept]](#Section_5_C_Model_18)  [(19) Prepartum Dep Sx](#Section_5_C_Model_19)  [(20) Prepartum Anx Sx](#Section_5_C_Model_20)  [(21) EPDS[T1-raw]](#Section_5_C_Model_21)  [(22) CESD[T1-raw]](#Section_5_C_Model_22) | [(23) BAI[T1-raw]](#Section_5_C_Model_23)  [(24) CESD[T4-raw]](#Section_5_C_Model_24)  [(25) BAI[T4-raw]](#Section_5_C_Model_25)  [(26) CESD[T1-raw] + CESD[T4-raw]](#Section_5_C_Model_26)  [(27) BAI[T1-raw] + BAI[T4-raw]](#Section_5_C_Model_27)  [(28) Dep/Anx Hx](#Section_5_C_Model_28)  [(29) Dep/Anx Dx](#Section_5_C_Model_29)  [(30) Dep Dose + Anx Dose](#Section_5_C_Model_30)  [(31) Cortisol[T4-linear-term] + Cortisol[T4-intercept]](#Section_5_C_Model_31) |
| **InfOwnPos > InfOtherPos** | |
| [(32) CESD[T1-4]](#Section_5_D_Model_32)  [(33) BAI[T1-4]](#Section_5_D_Model_33) | [(34) Dep Hx + Dep Dx](#Section_5_D_Model_34)  [(35) Anx Hx + Anx Dx](#Section_5_D_Model_35) |
| **InfOwnNeg > InfOtherNeg** | |
| [(36) CESD[T1-4]](#Section_5_E_Model_36)  [(37) BAI[T1-4]](#Section_5_E_Model_37) | [(38) Dep Hx + Dep Dx](#Section_5_E_Model_38)  [(39) Anx Hx + Anx Dx](#Section_5_E_Model_39) |
| *Key:*  (Continuous)  *CESD[T4-slope] = estimated change in CESD (depression) symptom scores across all 4 postnatal assessments; CESD[T4-intercept] = estimated CESD scores at time 4 assessment; BAI[T4-intercept] = estimated BAI (anxiety) symptom scores at time 4 assessment; CESD[T1-4-intercept] = estimated average CESD scores across all 4 post-natal assessments (same as used in models reported in main manuscript); BAI[T1-4-intercept] = estimated average BAI scores (same as used in models reported in main manuscript); Prepartum Dep Sx =Retrospective report of depression symptoms during pregnancy; Prepartum Anx Sx = Retrospective report of depression symptoms during pregnancy; EPSD[T1-raw] = Observed EPSD scores at time 1; CESD[T1-raw] = Observed CESD scores at time 1; BAI[T1-raw] = Observed BAI scores at time 1; CESD[T4-raw] = Observed CESD scores at time 4; BAI[T4-raw] = Observed BAI scores at time 4; Cortisol[T4-linear-term] = linear rate of change of cortisol stress response at time 4; Cortisol[T4- intercept] = Estimated mean cortisol levels at time 4 assessment; LE[T1-raw] = Life Experiences Survey at time 1 assessment; PS[T1-raw] = Parenting Stress Scale at time 1 assessment*  (Categorical)  *Dep Hx = history of depressive disorder; Dep Dx = current depressive disorder; Anx Hx = history of anxiety disorder; Anx Dx = current anxiety disorder; Dep/Anx Hx = history of depression and/or anxiety disorder; Dep/Anx Dx = current depression and/or anxiety disorder; Dep Dose = amount of exposure to depressive disorder (history and/or current); Anx Dose = amount of exposure to anxiety disorder (history and/or current)* | |

*Abbreviation Key:*

*SFG = Superior frontal gyrus; IFG = Inferior frontal gyrus; IP = Inferior parietal; MFG = Middle frontal gyrus; STG = Superior temporal gyrus; MTG = Middle temporal gyrus; OUTSIDE = not defined in the AAL atlas.*

**[Section V.](#Table_of_Contents)A.** Table of Associations for Supplementary Models


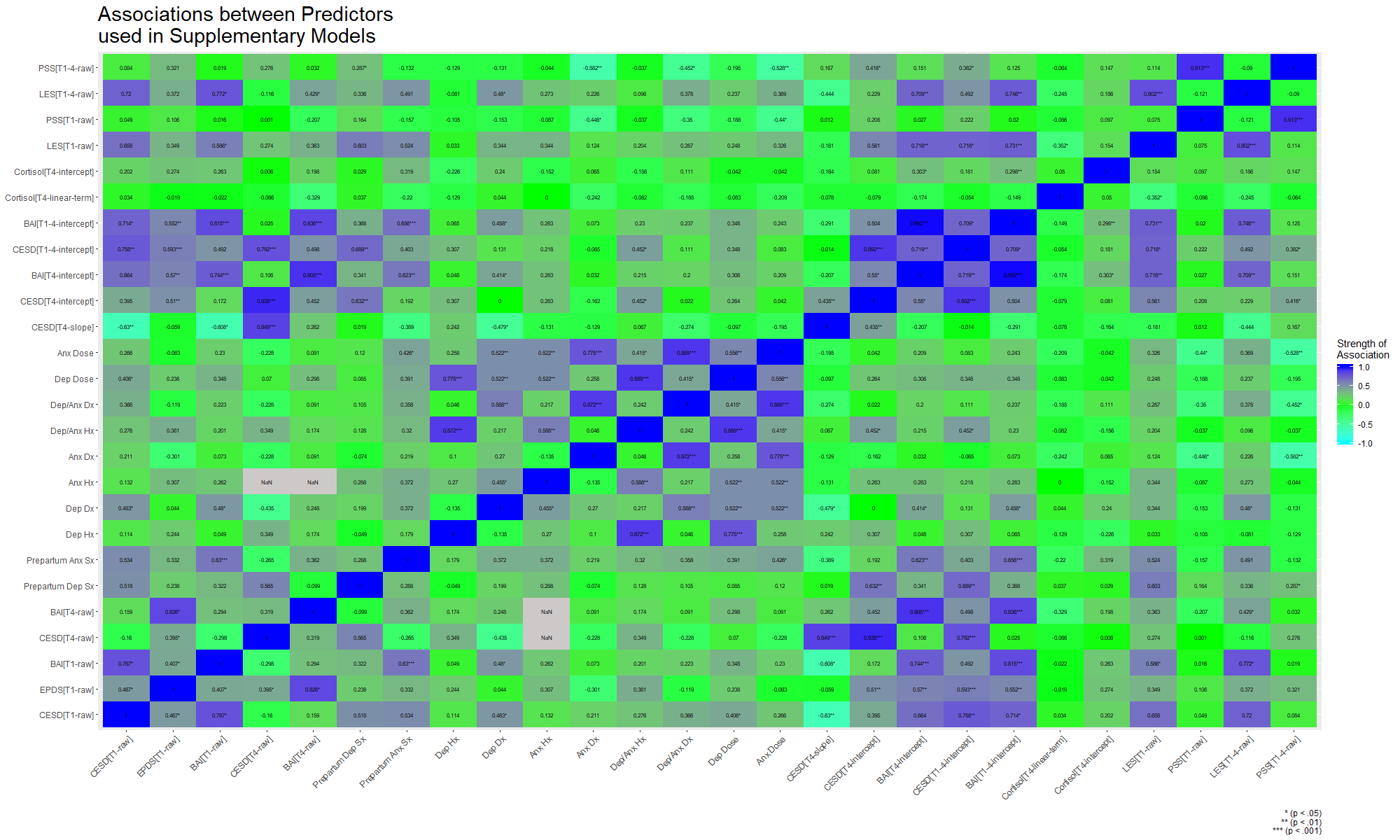
Association Matrix between predictors used in supplementary models. See key in model summary table in [Section IV](#Section_5_Table) above for descriptions of variables used. *Significance delineated at uncorrected thresholds.* (A) *Denotes Pearson’s correlation between a continuous-continuous pair. All other associations reported (categorical-categorical or categorical-continuous pairs) are reported using Spearman’s rank correlation. Regressors from models reported in the main manuscript are also included for comparison. Note that due to concomitant removal of variance, not all associations with anxiety history could be calculated after deleting incomplete observation pairs.*

**[Section V.B.](#Table_of_Contents)** Regression against (InfOwnPos > InfOwnNeg)

*------------------------------------------------------------------------------------------------------------------------------------------*

***[Model 5:](#Section_5_Table)*** $\left( InfOwnPos > InfOwnNeg \right)= \beta_{0}+ \beta_{1}\left( CESD[T4\text{-}slope] \right)+\beta_{2}(CESD[T4\text{-}intercept])$

*------------------------------------------------------------------------------------------------------------------------------------------*

**( InfOwnPos > InfOwnNeg ) – positive association with CESD Time4 Slope**

| **Cluster** | **Regions** | **Number of voxels in region** | **Cluster size** | **FWE-corrected p-value** |
| --- | --- | --- | --- | --- |
| 1 | R SFG, dorsolateral R Middle frontal gyrus R SFG, medial R Supplementary motor area R Precentral gyrus L SFG, medial R IFG, triangular part OUTSIDE R IFG, opercular part L SFG, dorsolateral | 701 582 212 135 113 90 77 55 22 21 | 2008 | < 0.001 |
| 2 | OUTSIDE L Precentral gyrus L Postcentral gyrus L Superior parietal gyrus L Precuneus L IP, supramarginal and angular gyri L SFG, dorsolateral L Middle frontal gyrus L Angular gyrus | 301 274 273 201 107 76  35 15 7 | 1289 | < 0.001 |
| 3 | R Angular gyrus R IP, supramarginal and angular gyri R Middle temporal gyrus R Middle occipital gyrus OUTSIDE R Superior parietal gyrus R Supramarginal gyrus R Superior temporal gyrus | 463 191  28 26 10 4 3 1 | 726 | < 0.001 |
| 4 | R Middle frontal gyrus R SFG, dorsolateral R SFG, medial OUTSIDE R SFG, medial orbital R MFG, orbital part | 221 170 50 32 8 1 | 482 | < 0.001 |
| 5 | R Postcentral gyrus R IP, supramarginal and angular gyri R Superior parietal gyrus R Precentral gyrus OUTSIDE | 179 54  15 12 1 | 261 | < 0.001 |
| 6 | OUTSIDE L Middle temporal gyrus L Inferior temporal gyrus L Fusiform gyrus L Hippocampus | 64 59 29 19 2 | 173 | 0.003 |
| 7 | L Inferior temporal gyrus L Fusiform gyrus L Middle temporal gyrus OUTSIDE | 82 16 15 13 | 126 | 0.014 |

*Significance threshold T(21) = 3.53; Cluster extent threshold = 126 voxels.*

**( InfOwnPos > InfOwnNeg ) – positive association with CESD Time4 Intercept**

| **Cluster** | **Regions** | **Number of voxels in region** | **Cluster size** | **FWE-corrected p-value** |
| --- | --- | --- | --- | --- |
| 1 | R Cerebellum 6 OUTSIDE R Cerebellum 8 R Cerebellum 9 R Crus Cerebellum 1 R Cerebellum 4-5 | 157 117 76 10 10 7 | 377 | < 0.001 |

*Significance threshold T(21) = 3.53; Cluster extent threshold = 377 voxels.*

**( InfOwnPos > InfOwnNeg ) – negative association with CESD Time4 Intercept**

| **Cluster** | **Regions** | **Number of voxels in region** | **Cluster size** | **FWE-corrected p-value** |
| --- | --- | --- | --- | --- |
| 1 | R SFG, dorsolateral L Paracentral lobule L Postcentral gyrus R Precentral gyrus R Supplementary motor area L Superior parietal gyrus OUTSIDE L Precuneus R Middle frontal gyrus L Precentral gyrus R Paracentral lobule L Supplementary motor area R Postcentral gyrus L SFG, dorsolateral | 354 255 176 167 114 102 75 71 71 71 68 60 40 10 | 1634 | < 0.001 |
| 2 | R Postcentral gyrus OUTSIDE R IP, supramarginal and angular gyri R Angular gyrus R Superior parietal gyrus R Precentral gyrus R Supramarginal gyrus | 359 203 169  137 131 21 2 | 1022 | < 0.001 |
| 3 | OUTSIDE L IP, but supramarginal and angular gyri L Postcentral gyrus L Supplementary motor area | 301 18  13 1 | 333 | < 0.001 |
| 4 | R SFG, dorsolateral R SFG, medial R Middle frontal gyrus OUTSIDE | 99 63 49 2 | 213 | < 0.001 |
| 5 | L Inferior temporal gyrus OUTSIDE L Fusiform gyrus L Temporal pole: STG L Parahippocampal gyrus L Middle temporal gyrus L Amygdala L IFG, orbital part | 74 39 25 23 14 9 8 5 | 197 | 0.001 |
| 6 | L Precentral gyrus L SFG, dorsolateral L Middle frontal gyrus | 104 6 2 | 112 | 0.025 |
| 7 | R SFG, dorsolateral R SFG, medial | 58 39 | 97 | 0.046 |

*Significance threshold T(21) = 3.53; Cluster extent threshold = 97 voxels.*

**( InfOwnPos > InfOwnNeg ) – negative association with Model Intercept**

| **Cluster** | **Regions** | **Number of voxels in region** | **Cluster size** | **FWE-corrected p-value** |
| --- | --- | --- | --- | --- |
| 1 | R Insula OUTSIDE R Rolandic operculum R Putamen R IFG, opercular part | 72 26 12 4 2 | 116 | 0.021 |

*Significance threshold T(21) = 3.53; Cluster extent threshold = 116 voxels.*

*------------------------------------------------------------------------------------------------------------------------------------------*

***[Model 6:](#Section_5_Table)*** $\left( InfOwnPos > InfOwnNeg \right)= \beta_{0}+ \beta_{1}\left( BAI[T4\text{-}intercept] \right)$

*------------------------------------------------------------------------------------------------------------------------------------------*

**( InfOwnPos > InfOwnNeg ) – negative association with BAI Time4 Intercept**

| **Cluster** | **Regions** | **Number of voxels in region** | **Cluster size** | **FWE-corrected p-value** |
| --- | --- | --- | --- | --- |
| 1 | OUTSIDE R Postcentral gyrus L Postcentral gyrus L Paracentral lobule R SFG, dorsolateral L IP, supramarginal and angular gyri R Supplementary motor area R Superior parietal gyrus L Precuneus L Precentral gyrus L Superior parietal gyrus R Precentral gyrus R IP, supramarginal and angular gyri R Precuneus L Supplementary motor area R Paracentral lobule R Middle frontal gyrus R Median cingulate and paracingulate gyri R Angular gyrus R Middle occipital gyrus L Median cingulate and paracingulate gyri L SFG, dorsolateral R Superior occipital gyrus L Middle occipital gyrus L Angular gyrus R Supramarginal gyrus R Posterior cingulate gyrus L Superior occipital gyrus L Middle frontal gyrus R SFG, medial L Posterior cingulate gyrus R Cuneus L Supramarginal gyrus L Anterior cingulate and paracingulate gyri R Calcarine | 2560 979 873 854 854 818  778 616 597 593 541 540 510  502 470 441 397 379  374 360 342  291 288 232 126 89 67 43 32 31 18 17 8 8  4 | 15632 | < 0.001 |
| 2 | R Middle frontal gyrus R SFG, dorsolateral R IFG, triangular part R SFG, medial OUTSIDE R SFG, orbital part | 453 202 119 54 18 2 | 848 | < 0.001 |
| 3 | OUTSIDE R IFG, orbital part R MFG, orbital part R SFG, orbital part R Caudate nucleus R Gyrus rectus R SFG, medial orbital | 67 65 38 22 4 3 1 | 200 | < 0.001 |
| 4 | L Crus Cerebellum 2 L Cerebellum 8 L Crus Cerebellum 1 L Cerebellum 7b | 98 20 17 14 | 149 | 0.004 |
| 5 | R Parahippocampal gyrus R Fusiform gyrus R Hippocampus OUTSIDE | 77 20 12 4 | 113 | 0.019 |
| 6 | OUTSIDE R Temporal pole: middle temporal gyrus R Middle temporal gyrus R Inferior temporal gyrus R Temporal pole: STG | 50 33 12 9 4 | 108 | 0.023 |
| 7 | L Middle occipital gyrus L Superior occipital gyrus | 92 15 | 107 | 0.024 |
| 8 | OUTSIDE L Middle frontal gyrus L IFG, triangular part L Insula | 58 40 4 1 | 103 | 0.029 |
| 9 | L Fusiform gyrus L Parahippocampal gyrus OUTSIDE L Hippocampus L Inferior temporal gyrus L Middle temporal gyrus | 45 35 7 6 4 2 | 99 | 0.034 |

*Significance threshold T(22) = 3.50; Cluster extent threshold = 99 voxels.*

**( InfOwnPos > InfOwnNeg ) – negative association with Model Intercept**

| **Cluster** | **Regions** | **Number of voxels in region** | **Cluster size** | **FWE-corrected p-value** |
| --- | --- | --- | --- | --- |
| 1 | R Insula OUTSIDE R Rolandic operculum R Putamen R IFG, opercular part | 74 25 12 4 2 | 117 | 0.016 |

*Significance threshold T(22) = 3.50; Cluster extent threshold = 117 voxels.*

*------------------------------------------------------------------------------------------------------------------------------------------*

***[Model 7:](#Section_5_Table)*** $\left( InfOwnPos > InfOwnNeg \right)= \beta_{0}+ \beta_{1}\left( CESD[T1\text{-}4\text{-}intercept] \right)$

$+\beta_{2}(BAI[T1\text{-}4\text{-}intercept])$

*------------------------------------------------------------------------------------------------------------------------------------------*

**( InfOwnPos > InfOwnNeg ) – positive association with CESD Time1-4 Intercept**

| **Cluster** | **Regions** | **Number of voxels in region** | **Cluster size** | **FWE-corrected p-value** |
| --- | --- | --- | --- | --- |
| 1 | R Cerebellum 6  R Crus Cerebellum 1  L Fusiform gyrus  L Lingual gyrus  R Lingual gyrus  R Calcarine fissure and surrounding cortex  L Cerebellum 6  OUTSIDE  L Crus Cerebellum 1  L Calcarine fissure and surrounding cortex  R Cuneus  R Cerebellum 8  Vermis 6  R Crus Cerebellum 2  Vermis 8  Vermis 7  R Superior occipital gyrus  Vermis 9  L Inferior occipital gyrus  R Fusiform gyrus  R Cerebellum 7b  L Middle occipital gyrus  L Middle temporal gyrus  R Cerebellum 9  R Cerebellum 4-5 | 372  313  271  198  163  162  148  148  141  94  64  52  51  45  35  30  30  26  20  19  14  5  4  1  1 | 2407 | < 0.001 |
| 2 | L Cuneus  L Superior occipital gyrus  L Middle occipital gyrus  L Calcarine fissure and surrounding cortex  OUTSIDE | 104  91  5  4  1 | 205 | < 0.001 |

*Significance threshold T(21) = 3.53; Cluster extent threshold = 205 voxels.*

**( InfOwnPos > InfOwnNeg ) – negative association with BAI Time1-4 Intercept**

| **Cluster** | **Regions** | **Number of voxels in region** | **Cluster size** | **FWE-corrected p-value** |
| --- | --- | --- | --- | --- |
| 1 | L Median cingulate and paracingulate gyri OUTSIDE L IP, supramarginal and angular gyri R Supplementary motor area R Median cingulate and paracingulate gyri L Precuneus L Superior parietal gyrus L Supplementary motor area L Postcentral gyrus L Paracentral lobule L Precentral gyrus R Paracentral lobule R Precuneus L Middle occipital gyrus L Angular gyrus R SFG, dorsolateral L Superior occipital gyrus R Posterior cingulate gyrus L Posterior cingulate gyrus L SFG, dorsolateral L Middle frontal gyrus L Supramarginal gyrus R Postcentral gyrus L Anterior cingulate and paracingulate gyri R Precentral gyrus | 535  476 456  402 368  366 315 302 282 273 221 183 88 52 47 41 28 27 24 17 14 11 9 9  3 | 4549 | < 0.001 |
| 2 | L Cuneus L Superior occipital gyrus R Lingual gyrus L Calcarine fissure and surrounding cortex R Calcarine fissure and surrounding cortex R Cuneus R Superior occipital gyrus OUTSIDE L Middle occipital gyrus R Inferior occipital gyrus R Middle occipital gyrus | 187 156 133 130  115  112 55 6 4 2 2 | 902 | < 0.001 |
| 3 | L Middle frontal gyrus OUTSIDE L IFG, triangular part L Insula L SFG, dorsolateral L IFG, opercular part | 273 68 52 8 6 1 | 408 | < 0.001 |
| 4 | R Middle frontal gyrus R SFG, dorsolateral OUTSIDE | 281 97 30 | 408 | < 0.001 |
| 5 | R Postcentral gyrus R IP, supramarginal and angular gyri OUTSIDE R Precentral gyrus R Supramarginal gyrus | 335 27  21 7 1 | 391 | < 0.001 |
| 6 | L Crus Cerebellum 1 L Crus Cerebellum 2 L Cerebellum 8 L Cerebellum 7b L Fusiform gyrus L Cerebellum 6 | 121 120 12 10 10 1 | 274 | < 0.001 |
| 7 | L Lingual gyrus L Calcarine fissure and surrounding cortex L Middle occipital gyrus L Cerebellum 6 L Superior occipital gyrus L Fusiform gyrus OUTSIDE | 101 39  34 11 9 5 4 | 203 | < 0.001 |
| 8 | R Cerebellum 6 R Crus Cerebellum 1 R Fusiform gyrus | 109 53 27 | 189 | 0.001 |
| 9 | L Fusiform gyrus OUTSIDE L Lingual gyrus L Cerebellum 6 L Cerebellum 4-5 L Inferior occipital gyrus | 84 43 13 12 2 1 | 155 | 0.003 |
| 10 | R Precentral gyrus OUTSIDE R SFG, dorsolateral R Middle frontal gyrus | 72 26 24 1 | 123 | 0.01 |
| 11 | L SFG, dorsolateral L Supplementary motor area OUTSIDE | 66 25 1 | 92 | 0.039 |
| 12 | R Cuneus R Precuneus R Superior occipital gyrus R Middle occipital gyrus | 53 30 8 1 | 92 | 0.039 |

*Significance threshold T(21) = 3.53; Cluster extent threshold = 92 voxels.*

**( InfOwnPos > InfOwnNeg ) – negative association with Model Intercept**

| **Cluster** | **Regions** | **Number of voxels in region** | **Cluster size** | **FWE-corrected p-value** |
| --- | --- | --- | --- | --- |
| 1 | R Insula OUTSIDE R Rolandic operculum R Putamen R IFG, opercular part | 68 28 12 4 2 | 114 | 0.015 |

*Significance threshold T(21) = 3.53; Cluster extent threshold = 114 voxels.*

*------------------------------------------------------------------------------------------------------------------------------------------*

***[Model 8:](#Section_5_Table)*** $\left( InfOwnPos > InfOwnNeg \right)= \beta_{0}+ \beta_{1}\left( Dep/Anx Hx \right)$

*------------------------------------------------------------------------------------------------------------------------------------------*

**( InfOwnPos > InfOwnNeg ) – positive association with Dep/Anx History**

| **Cluster** | **Regions** | **Number of voxels in region** | **Cluster size** | **FWE-corrected p-value** |
| --- | --- | --- | --- | --- |
| 1 | OUTSIDE L Middle temporal gyrus L Superior temporal gyrus | 107 14 4 | 125 | 0.018 |

*Significance threshold T(22) = 3.50; Cluster extent threshold = 125 voxels.*

*------------------------------------------------------------------------------------------------------------------------------------------*

***[Model 9:](#Section_5_Table)*** $\left( InfOwnPos > InfOwnNeg \right)= \beta_{0}+ \beta_{1}\left( Dep/Anx Dx \right)$

*------------------------------------------------------------------------------------------------------------------------------------------*

**( InfOwnPos > InfOwnNeg ) – negative association with Current Dep/Anx Diagnosis**

| **Cluster** | **Regions** | **Number of voxels in region** | **Cluster size** | **FWE-corrected p-value** |
| --- | --- | --- | --- | --- |
| 1 | R IFG, opercular part R Precentral gyrus R IFG, triangular part R Middle frontal gyrus OUTSIDE | 340 135 45 42 14 | 576 | < 0.001 |
| 2 | R Precentral gyrus R Middle frontal gyrus OUTSIDE R SFG, dorsolateral | 289 128 54 40 | 511 | < 0.001 |
| 3 | R Postcentral gyrus R IP, supramarginal and angular gyri R Superior parietal gyrus OUTSIDE R Angular gyrus | 144 84  79 22 8 | 337 | < 0.001 |
| 4 | R Angular gyrus OUTSIDE R Middle temporal gyrus R Superior temporal gyrus | 100 71 22 12 | 205 | 0.001 |
| 5 | R IFG, triangular part R Middle frontal gyrus OUTSIDE | 114 31 4 | 149 | 0.006 |
| 6 | L Superior parietal gyrus L IP, supramarginal and angular gyri L Postcentral gyrus L Precuneus | 124 16  4 1 | 145 | 0.006 |
| 7 | L Precentral gyrus L IFG, opercular part L Postcentral gyrus  L IFG, triangular part OUTSIDE | 94 21 11 10 7 | 143 | 0.007 |
| 8 | L Postcentral gyrus L IP, supramarginal and angular gyri L Superior parietal gyrus | 66 24  6 | 96 | 0.045 |
| Clusters Spanning a Single Region | L Middle occipital gyrus |  | 94 | 0.049 |

*Significance threshold T(22) = 3.50; Cluster extent threshold = 94 voxels.*

*------------------------------------------------------------------------------------------------------------------------------------------*

***[Model 11:](#Section_5_Table)*** $\left( InfOwnPos > InfOwnNeg \right)= \beta_{0}+ \beta_{1}\left( Anx Dose \right)$

*------------------------------------------------------------------------------------------------------------------------------------------*

**( InfOwnPos > InfOwnNeg ) – negative association with Anxiety Dose**

| **Cluster** | **Regions** | **Number of voxels in region** | **Cluster size** | **FWE-corrected p-value** |
| --- | --- | --- | --- | --- |
| 1 | R Precentral gyrus OUTSIDE R Middle frontal gyrus R Supplementary motor area R SFG, dorsolateral | 126 107 72 16 12 | 333 | < 0.001 |
| 2 | R IFG, opercular part R Precentral gyrus R IFG, triangular part | 105 78 1 | 184 | 0.002 |
| 3 | L Precentral gyrus L Middle frontal gyrus L Postcentral gyrus L IFG, opercular part | 92 30 10 5 | 137 | 0.009 |
| 4 | L IFG, triangular part L Middle frontal gyrus | 108 20 | 128 | 0.013 |
| 5 | R IFG, triangular part R Middle frontal gyrus OUTSIDE | 53 46 3 | 102 | 0.037 |

*Significance threshold T(22) = 3.50; Cluster extent threshold = 102 voxels.*

*------------------------------------------------------------------------------------------------------------------------------------------*

***[Model 12:](#Section_5_Table)*** $\left( InfOwnPos > InfOwnNeg \right)= \beta_{0}+ \beta_{1}\left( Cortisol[T4\text{-}linear\text{-t}erm] \right)$

$+ \beta_{2}\left( Cortisol[T4\text{-}intercept] \right)$

*------------------------------------------------------------------------------------------------------------------------------------------*

**( InfOwnPos > InfOwnNeg ) – positive association with Cortisol Time4 Linear Term**

| **Cluster** | **Regions** | **Number of voxels in region** | **Cluster size** | **FWE-corrected p-value** |
| --- | --- | --- | --- | --- |
| 1 | L Supramarginal gyrus L Angular gyrus L Middle temporal gyrus L IP, supramarginal and angular gyri | 51 43 14 3 | 111 | 0.031 |

*Significance threshold T(21) = 3.53; Cluster extent threshold = 111 voxels.*

**( InfOwnPos > InfOwnNeg ) – negative association with Model Intercept**

| **Cluster** | **Regions** | **Number of voxels in region** | **Cluster size** | **FWE-corrected p-value** |
| --- | --- | --- | --- | --- |
| 1 | R Insula OUTSIDE R Rolandic operculum R IFG, opercular part R Putamen | 101 19 15 4 1 | 140 | 0.010 |

*Significance threshold T(21) = 3.53; Cluster extent threshold = 140 voxels.*

*------------------------------------------------------------------------------------------------------------------------------------------*

***[Model 13:](#Section_5_Table)*** $\left( InfOwnPos > InfOwnNeg \right)= \beta_{0}+ \beta_{1}\left( LES[T1\text{-}raw] \right)$

$+ \beta_{2}\left( PSS[T1\text{-}raw] \right)$

*------------------------------------------------------------------------------------------------------------------------------------------*

**( InfOwnPos > InfOwnNeg ) – negative association with FES Time1 Raw Scores**

| **Cluster** | **Regions** | **Number of voxels in region** | **Cluster size** | **FWE-corrected p-value** |
| --- | --- | --- | --- | --- |
| 1 | OUTSIDE  R Postcentral gyrus  L Paracentral lobule  L Postcentral gyrus  R Precentral gyrus  L IP, supramarginal and angular gyri  L Precuneus  R Superior parietal gyrus  L Superior parietal gyrus  L Precentral gyrus  R Supplementary motor area  R Paracentral lobule  R SFG, dorsolateral  R Angular gyrus  R Precuneus  R IP, supramarginal and angular gyri  L SFG, dorsolateral  L Supplementary motor area  R Superior occipital gyrus  L Middle occipital gyrus  R Median cingulate and paracingulate gyri  R Middle frontal gyrus  L Angular gyrus  L Median cingulate and paracingulate gyri  L Middle frontal gyrus  R Middle occipital gyrus  R Supramarginal gyrus  L Superior occipital gyrus  R Superior temporal gyrus  L Anterior cingulate and paracingulate gyri  R Cuneus  R Posterior cingulate gyrus | 2486  1211  762  719  703  650  638  591  553  494  420  384  362  299  273  269  195  160  123  110  100  96  63  58  57  54  22  21  16  13  2  2 | 11906 | < 0.001 |
| 2 | R Middle frontal gyrus  R IFG, triangular part  OUTSIDE | 130  66  28 | 224 | < 0.001 |
| 3 | L Inferior temporal gyrus  L Fusiform gyrus  OUTSIDE  L Middle temporal gyrus  L Hippocampus | 127  38  24  11  3 | 203 | 0.001 |
| 4 | OUTSIDE  L Inferior temporal gyrus  L Temporal pole: STG  L Fusiform gyrus  L Middle temporal gyrus  L Parahippocampal gyrus  L Temporal pole: MTG  L Amygdala  L Hippocampus  L IFG, orbital part | 67  51  30  24  8  4  3  3  3  1 | 194 | 0.001 |
| 5 | OUTSIDE  R SFG, dorsolateral  R Middle frontal gyrus | 69  46  40 | 155 | 0.004 |
| 6 | L Middle frontal gyrus  L IFG, triangular part | 59  58 | 117 | 0.016 |
| 7 | R SFG, dorsolateral  R SFG, medial  OUTSIDE | 79  35  2 | 116 | 0.017 |

*Significance threshold T(21) = 3.53; Cluster extent threshold = 116 voxels.*

**( InfOwnPos > InfOwnNeg ) – negative association with Model Intercept**

| **Cluster** | **Regions** | **Number of voxels in region** | **Cluster size** | **FWE-corrected p-value** |
| --- | --- | --- | --- | --- |
| 1 | R Insula  OUTSIDE  R Rolandic operculum  R IFG, opercular part  R Putamen | 79  25  12  5  2 | 123 | 0.013 |

*Significance threshold T(21) = 3.53; Cluster extent threshold = 123 voxels.*

**[Section V.C.](#Table_of_Contents)** Regression against (InfPos > InfNeg)

*------------------------------------------------------------------------------------------------------------------------------------------*

***[Model 14:](#Section_5_Table)*** $\left( InfPos > InfNeg \right)= \beta_{0}+ \beta_{1}\left( CESD[T4\text{-}slope] \right)+\beta_{2}(CESD[T4\text{-}intercept])$

*------------------------------------------------------------------------------------------------------------------------------------------*

**( InfPos > InfNeg ) – positive association with CESD Time4 Slope**

| **Cluster** | **Regions** | **Number of voxels in region** | **Cluster size** | **FWE-corrected p-value** |
| --- | --- | --- | --- | --- |
| 1 | R Middle frontal gyrus R SFG, dorsolateral R IFG, triangular part OUTSIDE R SFG, medial R IFG, opercular part R SFG, medial orbital R Insula R IFG, orbital part R MFG, orbital part R SFG, orbital part | 424 198 184 182 100 30 18 17 4 4 3 | 1164 | < 0.001 |
| 2 | R IP, supramarginal and angular gyri R Angular gyrus R Supramarginal gyrus OUTSIDE R Superior parietal gyrus | 228  112 53 23 1 | 417 | < 0.001 |
| 3 | OUTSIDE L Inferior temporal gyrus L Middle temporal gyrus L Hippocampus L Fusiform gyrus | 158 57 29 23 14 | 281 | < 0.001 |
| 4 | OUTSIDE L Middle frontal gyrus L IFG, triangular part L Insula L Anterior cingulate and paracingulate gyri L Caudate nucleus | 125 88 22 3 2  1 | 241 | < 0.001 |
| 5 | R SFG, medial R SFG, dorsolateral L SFG, medial R Supplementary motor area | 138 61 7 2 | 208 | < 0.001 |
| 6 | OUTSIDE R Thalamus R Caudate nucleus R Median cingulate and paracingulate gyri | 119 27 8 1 | 155 | 0.003 |
| 7 | L Precentral gyrus L SFG, dorsolateral L Middle frontal gyrus OUTSIDE | 127 11 7 1 | 146 | 0.005 |
| 8 | R SFG, dorsolateral R Middle frontal gyrus R Supplementary motor area | 88 16 6 | 110 | 0.021 |
| 9 | OUTSIDE L Angular gyrus L IP, supramarginal and angular gyri | 102 3 3 | 108 | 0.022 |
| 10 | R Supplementary motor area R SFG, dorsolateral OUTSIDE | 73 29 1 | 103 | 0.028 |
| 11 | R IFG, triangular part R Middle frontal gyrus | 78 16 | 94 | 0.041 |

*Significance threshold T(21) = 3.53; Cluster extent threshold = 94 voxels.*

**( InfPos > InfNeg ) – negative association with CESD Time4 Intercept**

| **Cluster** | **Regions** | **Number of voxels in region** | **Cluster size** | **FWE-corrected p-value** |
| --- | --- | --- | --- | --- |
| 1 | R Middle frontal gyrus OUTSIDE R IFG, orbital part R IFG, triangular part R SFG, medial R SFG, dorsolateral R IFG, opercular part R Insula R MFG, orbital part R SFG, medial orbital R SFG, orbital part | 337 211 189 175 137 129 76 19 5 2 2 | 1282 | < 0.001 |
| 2 | R IP, but supramarginal and angular gyri R Angular gyrus OUTSIDE R Supramarginal gyrus R Superior parietal gyrus | 254  155 128 12 11 | 560 | < 0.001 |
| 3 | OUTSIDE L Thalamus L Hippocampus L Posterior cingulate gyrus | 198 93 10 4 | 305 | < 0.001 |
| 4 | OUTSIDE L IP, supramarginal and angular gyri L Postcentral gyrus L Superior parietal gyrus | 155 89  24 2 | 270 | < 0.001 |
| 5 | L Middle frontal gyrus L IFG, triangular part OUTSIDE | 129 80 52 | 261 | < 0.001 |
| 6 | L Superior temporal gyrus L Inferior temporal gyrus OUTSIDE L Middle temporal gyrus L Temporal pole: STG L Fusiform gyrus L Parahippocampal gyrus L IFG, orbital part | 62 54 40 27 22 18 8 2 | 233 | < 0.001 |
| 7 | L Precuneus L Superior parietal gyrus | 145 40 | 185 | 0.001 |
| 8 | R SFG, dorsolateral R Middle frontal gyrus R Supplementary motor area | 134 14 7 | 155 | 0.003 |
| 9 | OUTSIDE L Inferior temporal gyrus L Fusiform gyrus L Hippocampus L Middle temporal gyrus L Parahippocampal gyrus | 49 41 28 22 4 1 | 145 | 0.005 |
| 10 | OUTSIDE R Thalamus | 77 45 | 122 | 0.013 |
| 11 | OUTSIDE R Thalamus R Hippocampus | 90 21 10 | 121 | 0.013 |
| 12 | Vermis 4-5 R Cerebellum 4-5 Vermis 3 | 102 3 1 | 106 | 0.024 |
| 13 | OUTSIDE L Anterior cingulate and paracingulate gyri R Caudate nucleus R Anterior cingulate and paracingulate gyri | 89 5  3 3 | 100 | 0.032 |

*Significance threshold T(21) = 3.53; Cluster extent threshold = 100 voxels.*

**( InfPos > InfNeg ) – positive association with Model Intercept**

| **Cluster** | **Regions** | **Number of voxels in region** | **Cluster size** | **FWE-corrected p-value** |
| --- | --- | --- | --- | --- |
| 1 | R Lingual gyrus R Calcarine fissure and surrounding cortex | 128 1 | 129 | 0.041 |
| 2 | L Lingual gyrus L Calcarine fissure and surrounding cortex L Middle occipital gyrus L Superior occipital gyrus | 49 39  4 2 | 94 | 0.009 |

*Significance threshold T(21) = 3.53; Cluster extent threshold = 94 voxels.*

**( InfPos > InfNeg ) – negative association with Model Intercept**

| **Cluster** | **Regions** | **Number of voxels in region** | **Cluster size** | **FWE-corrected p-value** |
| --- | --- | --- | --- | --- |
| 1 | L Insula OUTSIDE L Superior temporal gyrus L Rolandic operculum L Putamen L Heschl gyrus L IFG, opercular part L IFG, triangular part L Postcentral gyrus L IFG, orbital part L Middle temporal gyrus L Pallidum L Amygdala | 589 235 217 133 102 74 25 4 3 3 2 2 2 | 1391 | < 0.001 |
| 2 | R Insula R Rolandic operculum R Superior temporal gyrus R Amygdala OUTSIDE R Heschl gyrus R Putamen R IFG, opercular part R Hippocampus R Temporal pole: STG R Precentral gyrus R Pallidum | 288 272 190 90 78 53 51 41 20 17 2 1 | 1103 | < 0.001 |
| Clusters Spanning a Single Region | OUTSIDE |  | 103 | 0.028 |

*Significance threshold T(21) = 3.53; Cluster extent threshold = 103 voxels.*

*------------------------------------------------------------------------------------------------------------------------------------------*

***[Model 15:](#Section_5_Table)*** $\left( InfPos > InfNeg \right)= \beta_{0}+ \beta_{1}\left( CESD[T1\text{-}4\text{-}intercept] \right)$

*------------------------------------------------------------------------------------------------------------------------------------------*

**( InfPos > InfNeg ) – negative association with CESD Time1-4 Intercept**

| **Cluster** | **Regions** | **Number of voxels in region** | **Cluster size** | **FWE-corrected p-value** |
| --- | --- | --- | --- | --- |
| 1 | R Middle frontal gyrus R IFG, orbital part OUTSIDE R IFG, triangular part R Insula R SFG, dorsolateral R MFG, orbital part | 246 212 183 53 21 7 4 | 726 | < 0.001 |
| 2 | R IP, supramarginal and angular gyri R Angular gyrus OUTSIDE R Superior parietal gyrus R Supramarginal gyrus | 224  152 152 11 6 | 545 | < 0.001 |
| 3 | OUTSIDE L Thalamus L Hippocampus L Posterior cingulate gyrus | 224 135 13 8 | 380 | < 0.001 |
| 4 | OUTSIDE L IP, supramarginal and angular gyri L Postcentral gyrus | 225 82  25 | 332 | < 0.001 |
| 5 | L Superior temporal gyrus L Inferior temporal gyrus OUTSIDE L Middle temporal gyrus L Temporal pole: STG L Fusiform gyrus L Parahippocampal gyrus L IFG, orbital part | 71 59 47 45 27 22 11 3 | 285 | < 0.001 |
| 6 | L Middle frontal gyrus L IFG, triangular part OUTSIDE | 98 58 47 | 203 | < 0.001 |
| 7 | R SFG, medial R SFG, dorsolateral OUTSIDE | 133 67 1 | 201 | < 0.001 |
| 8 | R SFG, dorsolateral R SFG, medial R Supplementary motor area R Middle frontal gyrus | 159 12 7 7 | 185 | < 0.001 |
| 9 | L Precuneus L Superior parietal gyrus | 148 35 | 183 | 0.001 |
| 10 | OUTSIDE R Amygdala R Hippocampus R Temporal pole: STG R Superior temporal gyrus R Middle temporal gyrus R Insula R Putamen R Fusiform gyrus | 79 24 24 18 16 3 3 1 1 | 169 | 0.002 |
| 11 | OUTSIDE R Thalamus R Hippocampus R Posterior cingulate gyrus | 102 25 12 1 | 140 | 0.007 |
| 12 | Vermis 4-5 R Cerebellum 4-5 Vermis 3 | 132 3 2 | 137 | 0.008 |
| 13 | R IFG, opercular part R IFG, triangular part OUTSIDE | 77 42 4 | 123 | 0.013 |
| 14 | OUTSIDE R Thalamus | 74 40 | 114 | 0.019 |
| 15 | L Insula L IFG, opercular part L Rolandic operculum L Precentral gyrus OUTSIDE L Putamen | 68 18 13 7 5 2 | 113 | 0.02 |
| 16 | R Hippocampus R Parahippocampal gyrus OUTSIDE R Fusiform gyrus R Putamen | 55 30 15 6 1 | 107 | 0.026 |

*Significance threshold T(22) = 3.50; Cluster extent threshold = 107 voxels.*

**( InfPos > InfNeg ) – positive association with Model Intercept**

| **Cluster** | **Regions** | **Number of voxels in region** | **Cluster size** | **FWE-corrected p-value** |
| --- | --- | --- | --- | --- |
| 1 | R Lingual gyrus R Calcarine fissure and surrounding cortex | 132 2 | 134 | 0.009 |

*Significance threshold T(22) = 3.50; Cluster extent threshold = 134 voxels.*

**( InfPos > InfNeg ) – negative association with Model Intercept**

| **Cluster** | **Regions** | **Number of voxels in region** | **Cluster size** | **FWE-corrected p-value** |
| --- | --- | --- | --- | --- |
| 1 | L Insula OUTSIDE L Superior temporal gyrus L Rolandic operculum L Putamen L Heschl gyrus L IFG, opercular part L IFG, triangular part L Amygdala L Postcentral gyrus L IFG, orbital part L Middle temporal gyrus L Pallidum | 628 247 235 140 122 79 27 5 4 3 3 2 2 | 1497 | < 0.001 |
| 2 | R Rolandic operculum R Insula R Superior temporal gyrus R Heschl gyrus R IFG, opercular part OUTSIDE R Putamen R Precentral gyrus R Temporal pole: STG | 299 295 188 50 44 44 5 5 3 | 933 | < 0.001 |
| 3 | R Amygdala R Putamen OUTSIDE R Temporal pole: STG R Hippocampus R Pallidum R Parahippocampal gyrus | 87 39 32 16 16 1 1 | 192 | 0.001 |

*Significance threshold T(22) = 3.50; Cluster extent threshold = 192 voxels.*

*------------------------------------------------------------------------------------------------------------------------------------------*

***[Model 16:](#Section_5_Table)*** $\left( InfPos > InfNeg \right)= \beta_{0}+ \beta_{1}\left( BAI[T4\text{-}intercept] \right)$

*------------------------------------------------------------------------------------------------------------------------------------------*

**( InfPos > InfNeg ) – negative association with BAI Time4 Intercept**

| **Cluster** | **Regions** | **Number of voxels in region** | **Cluster size** | **FWE-corrected p-value** |
| --- | --- | --- | --- | --- |
| 1 | OUTSIDE R Middle frontal gyrus R SFG, dorsolateral L IP, supramarginal and angular gyri R IP, supramarginal and angular gyri L Postcentral gyrus R Precuneus L Median cingulate and paracingulate gyri L Precuneus R Superior occipital gyrus R Angular gyrus R Median cingulate and paracingulate gyri L Precentral gyrus R Postcentral gyrus L Paracentral lobule R Middle occipital gyrus L Superior parietal gyrus R SFG, medial R Superior parietal gyrus L Middle occipital gyrus R Precentral gyrus R Paracentral lobule L Superior temporal gyrus L Angular gyrus R IFG, triangular part R Supplementary motor area L Superior occipital gyrus L Supplementary motor area R Supramarginal gyrus L Supramarginal gyrus R Posterior cingulate gyrus L Posterior cingulate gyrus L SFG, dorsolateral L Middle temporal gyrus R IFG, orbital part R Cuneus L SFG medial L Anterior cingulate and paracingulate gyri L Middle frontal gyrus R Anterior cingulate and paracingulate gyri R SFG, orbital part | 1742 933 876 870  658  531 500 492  469 467 465 460  448 435 433 428 410 378 362 309 247 232 228 228 189 178 158 151 150 145 143 98 91 86 38 29 28 25  11 8  5 | 14134 | < 0.001 |
| 2 | OUTSIDE L Thalamus R Thalamus R Hippocampus R Parahippocampal gyrus R Fusiform gyrus R Cerebellum 4-5 L Hippocampus R Cerebellum 6 L Parahippocampal gyrus R Lingual gyrus | 586 339 171 66 59 57 52 24 11 3 1 | 1369 | < 0.001 |
| 3 | L Middle frontal gyrus L IFG, triangular part OUTSIDE L SFG, dorsolateral L Precentral gyrus L IFG, opercular part | 515 190 94 3 3 2 | 807 | < 0.001 |
| 4 | OUTSIDE L Superior temporal gyrus L Insula L Inferior temporal gyrus L Fusiform gyrus L Middle temporal gyrus L Temporal pole: STG L Hippocampus L Amygdala L IFG, orbital part L Rolandic operculum L Parahippocampal gyrus | 192 181 106 99 61 59 45 19 8 4 2 1 | 777 | < 0.001 |
| 5 | OUTSIDE R Insula R Temporal pole: STG R Middle temporal gyrus R Temporal pole: MTG R Inferior temporal gyrus R Superior temporal gyrus R IFG, orbital part R Putamen R Hippocampus R Fusiform gyrus R Amygdala | 233 201 130 59 32 29 27 23 12 6 5 3 | 760 | < 0.001 |
| 6 | Vermis 4-5 Vermis 3 Vermis 6 L Cerebellum 4-5 R Cerebellum 4-5 OUTSIDE L Lingual gyrus R Lingual gyrus L Cerebellum 3 | 292 55 38 23 22 20 13 5 3 | 471 | < 0.001 |
| 7 | L Crus Cerebellum 2 L Crus Cerebellum 1 L Cerebellum 8 L Cerebellum 9 L Cerebellum 7b L Cerebellum 6 | 173 146 65 48 31 2 | 465 | < 0.001 |
| 8 | L Lingual gyrus L Cerebellum 4-5 L Fusiform gyrus L Cerebellum 6 | 91 74 53 22 | 240 | < 0.001 |
| 9 | L SFG, medial L SFG, dorsolateral L Middle frontal gyrus | 63 42 14 | 119 | 0.01 |
| 10 | OUTSIDE R Anterior cingulate and paracingulate gyri R Caudate nucleus | 85 17  1 | 103 | 0.02 |
| 11 | R Cerebellum 6 R Fusiform gyrus | 68 17 | 85 | 0.047 |

*Significance threshold T(22) = 3.50; Cluster extent threshold = 85 voxels.*

**( InfPos > InfNeg ) – positive association with Model Intercept**

| **Cluster** | **Regions** | **Number of voxels in region** | **Cluster size** | **FWE-corrected p-value** |
| --- | --- | --- | --- | --- |
| 1 | R Lingual gyrus R Calcarine fissure and surrounding cortex | 159 6 | 165 | 0.001 |
| 2 | L Lingual gyrus L Calcarine fissure and surrounding cortex L Middle occipital gyrus L Superior occipital gyrus | 57 30  2 2 | 91 | 0.035 |

*Significance threshold T(22) = 3.50; Cluster extent threshold = 91 voxels.*

**( InfPos > InfNeg ) – negative association with Model Intercept**

| **Cluster** | **Regions** | **Number of voxels in region** | **Cluster size** | **FWE-corrected p-value** |
| --- | --- | --- | --- | --- |
| 1 | L Insula OUTSIDE L Superior temporal gyrus L Rolandic operculum L Putamen L Heschl gyrus L Hippocampus L IFG, opercular part L Parahippocampal gyrus L Amygdala L IFG, orbital part L Postcentral gyrus L Temporal pole: STG L Pallidum L Lingual gyrus L IFG, triangular part L Middle temporal gyrus | 633 273 242 142 123 72 29 21 9 6 5 3 2 2 2 2 1 | 1567 | < 0.001 |
| 2 | R Insula R Rolandic operculum R Superior temporal gyrus OUTSIDE R Amygdala R Heschl gyrus R IFG, opercular part R Putamen R Hippocampus R Temporal pole: STG R Precentral gyrus R Parahippocampal gyrus | 304 295 189 130 59 48 41 38 20 18 6 1 | 1149 | < 0.001 |
| 3 | OUTSIDE L Middle frontal gyrus L Anterior cingulate and paracingulate gyri L SFG, medial | 55 23 9 1 | 88 | 0.041 |
| 4 | Vermis 4-5 L Cerebellum 4-5 L Lingual gyrus OUTSIDE | 51 31 4 1 | 87 | 0.043 |

*Significance threshold T(22) = 3.50; Cluster extent threshold = 87 voxels.*

*------------------------------------------------------------------------------------------------------------------------------------------*

***[Model 17:](#Section_5_Table)*** $\left( InfPos > InfNeg \right)= \beta_{0}+ \beta_{1}\left( BAI[T1\text{-}4\text{-}intercept] \right)$

*------------------------------------------------------------------------------------------------------------------------------------------*

**( InfPos > InfNeg ) – negative association with BAI Time1-4 Intercept**

| **Cluster** | **Regions** | **Number of voxels in region** | **Cluster size** | **FWE-corrected p-value** |
| --- | --- | --- | --- | --- |
| 1 | OUTSIDE R Middle frontal gyrus R SFG, dorsolateral L IP, supramarginal and angular gyri R IP, supramarginal and angular gyri L Postcentral gyrus R Precuneus L Precentral gyrus R SFG, medial R Angular gyrus L Median cingulate and paracingulate gyri R Superior occipital gyrus L Precuneus R Postcentral gyrus L Superior parietal gyrus R Median cingulate and paracingulate gyri L Paracentral lobule R Middle occipital gyrus R Superior parietal gyrus L Thalamus L Middle occipital gyrus R Precentral gyrus R IFG, triangular part R Paracentral lobule L Angular gyrus L Superior temporal gyrus R Thalamus L Superior occipital gyrus R Supplementary motor area R Supramarginal gyrus L Supramarginal gyrus L Supplementary motor area R Posterior cingulate gyrus L SFG, dorsolateral L Middle temporal gyrus L Posterior cingulate gyrus R Hippocampus R Parahippocampal gyrus L SFG, medial R Fusiform gyrus R Cerebellum 4-5 R IFG, orbital part R Cuneus L Anterior cingulate and paracingulate gyri L Hippocampus L Cuneus L Middle frontal gyrus R Cerebellum 6 R Anterior cingulate and paracingulate gyri L Caudate nucleus L Pallidum R SFG, orbital part R SFG, medial orbital R Crus Cerebellum 2 R Lingual gyrus R Crus Cerebellum 1 L Putamen R Calcarine fissure and surrounding cortex L Parahippocampal gyrus | 2669 1055 989 899 682 669  553 530 529 511 488 484  481 480 457 439 430  419 392 377 292 292 260 247  247 233 224 211 209 175 152 148 133 115 97 81 79 59 55 54 48 47 29 27  20 19 17 14 11  10 7 6 5 4 3 2 1 1  1 | 17168 | < 0.001 |
| 2 | L Middle frontal gyrus L IFG, triangular part OUTSIDE L SFG, medial L SFG, dorsolateral L Precentral gyrus L SFG, orbital part L IFG, opercular part | 622 267 113 91 73 6 5 2 | 1179 | < 0.001 |
| 3 | OUTSIDE L Inferior temporal gyrus L Middle temporal gyrus L Superior temporal gyrus L Insula L Fusiform gyrus L Hippocampus L Temporal pole: STG L Amygdala L IFG, orbital part L Parahippocampal gyrus | 249 159 143 99 94 68 31 19 8 2 1 | 873 | < 0.001 |
| 4 | OUTSIDE R Insula R Temporal pole: STG R Middle temporal gyrus R Temporal pole: MTG R Inferior temporal gyrus R IFG, orbital part R Superior temporal gyrus R Putamen R Hippocampus R Fusiform gyrus R Amygdala | 232 214 120 63 31 28 27 24 15 5 4 2 | 765 | < 0.001 |
| 5 | Vermis 4-5 L Lingual gyrus L Cerebellum 4-5 Vermis 3 L Cerebellum 6 L Fusiform gyrus Vermis 6 R Cerebellum 4-5 OUTSIDE R Lingual gyrus L Cerebellum 3 | 282 98 72 48 48 48 39 25 19 5 2 | 686 | < 0.001 |
| 6 | L Crus Cerebellum 1 L Crus Cerebellum 2 L Cerebellum 8 L Cerebellum 9 L Fusiform gyrus L Cerebellum 7b L Inferior occipital gyrus L Cerebellum 6 | 212 189 93 56 52 34 15 6 | 657 | < 0.001 |
| 7 | OUTSIDE R Anterior cingulate and paracingulate gyri L Anterior cingulate and paracingulate gyri R Caudate nucleus | 103 19  3  1 | 126 | 0.007 |
| 8 | R Cerebellum 6 R Fusiform gyrus | 85 26 | 111 | 0.013 |
| 9 | R Precentral gyrus R IFG, opercular part R IFG, triangular part OUTSIDE | 47 38 10 7 | 102 | 0.02 |
| 10 | L Superior temporal gyrus L Temporal pole: STG L Insula L Rolandic operculum OUTSIDE | 66 10 5 2 1 | 84 | 0.047 |
| 11 | L IFG, opercular part L Precentral gyrus L IFG, triangular part L Rolandic operculum | 46 25 7 6 | 84 | 0.047 |

*Significance threshold T(22) = 3.50; Cluster extent threshold = 84 voxels.*

**( InfPos > InfNeg ) – positive association with Model Intercept**

| **Cluster** | **Regions** | **Number of voxels in region** | **Cluster size** | **FWE-corrected p-value** |
| --- | --- | --- | --- | --- |
| 1 | R Lingual gyrus R Calcarine fissure and surrounding cortex | 160 6 | 166 | 0.001 |
| 2 | L Lingual gyrus L Calcarine fissure and surrounding cortex L Middle occipital gyrus L Superior occipital gyrus | 57 30  2 2 | 91 | 0.033 |

*Significance threshold T(22) = 3.50; Cluster extent threshold = 91 voxels.*

**( InfPos > InfNeg ) – negative association with Model Intercept**

| **Cluster** | **Regions** | **Number of voxels in region** | **Cluster size** | **FWE-corrected p-value** |
| --- | --- | --- | --- | --- |
| 1 | L Insula OUTSIDE L Superior temporal gyrus L Rolandic operculum L Putamen L Heschl gyrus L Hippocampus L IFG, opercular part L Parahippocampal gyrus L IFG, orbital part L Amygdala L Postcentral gyrus L Middle temporal gyrus L Temporal pole: STG L Pallidum L Lingual gyrus L IFG, triangular part | 629 282 241 143 127 72 29 21 9 5 4 3 2 2 2 2 2 | 1575 | < 0.001 |
| 2 | R Insula R Rolandic operculum R Superior temporal gyrus OUTSIDE R Amygdala R Heschl gyrus R Putamen R IFG, opercular part R Hippocampus R Temporal pole: STG R Precentral gyrus R Parahippocampal gyrus | 313 300 191 139 57 48 45 44 20 17 6 1 | 1181 | < 0.001 |
| 3 | OUTSIDE L Middle frontal gyrus L Anterior cingulate and paracingulate gyri L SFG, medial | 55 24 9  2 | 90 | 0.035 |
| 4 | Vermis 4-5 L Cerebellum 4-5 L Lingual gyrus OUTSIDE | 49 33 4 1 | 87 | 0.041 |

*Significance threshold T(22) = 3.50; Cluster extent threshold = 87 voxels.*

*------------------------------------------------------------------------------------------------------------------------------------------*

***[Model 18:](#Section_5_Table)*** $\left( InfPos > InfNeg \right)= \beta_{0}+ \beta_{1}\left( CESD[T4\text{-}intercept] \right)+ \beta_{2}\left( BAI[T4\text{-}intercept] \right)$

*------------------------------------------------------------------------------------------------------------------------------------------*

**( InfPos > InfNeg ) – positive association with CESD Time4 Intercept**

| **Cluster** | **Regions** | **Number of voxels in region** | **Cluster size** | **FWE-corrected p-value** |
| --- | --- | --- | --- | --- |
| 1 | L Fusiform gyrus L Cerebellum 6 L Lingual gyrus L Crus Cerebellum 1 OUTSIDE | 62 52 32 2 1 | 149 | 0.003 |

*Significance threshold T(21) = 3.53; Cluster extent threshold = 149 voxels.*

**( InfPos > InfNeg ) – negative association with BAI Time4 Intercept**

| **Cluster** | **Regions** | **Number of voxels in region** | **Cluster size** | **FWE-corrected p-value** |
| --- | --- | --- | --- | --- |
| 1 | L IP, supramarginal and angular gyri L Precentral gyrus OUTSIDE R Median cingulate and paracingulate gyri L Median cingulate and paracingulate gyri L Postcentral gyrus L Superior parietal gyrus L Paracentral lobule L Superior occipital gyrus L Precuneus L Middle occipital gyrus L Superior temporal gyrus L Angular gyrus R Posterior cingulate gyrus L Supramarginal gyrus R Precuneus L SFG, dorsolateral L Supplementary motor area L Posterior cingulate gyrus L Middle temporal gyrus Vermis 4-5 L Middle frontal gyrus Vermis 3 | 649  485 443 348  346  334 249 186 176 165 149 115 115 90 79 64 48 45 42 18 16 10 1 | 4173 | < 0.001 |
| 2 | R IP, supramarginal and angular gyri OUTSIDE R SFG, dorsolateral R Precuneus R Superior occipital gyrus R Postcentral gyrus R Middle occipital gyrus R Angular gyrus R Precentral gyrus R Supramarginal gyrus R Superior parietal gyrus L Precuneus L Calcarine fissure and surrounding cortex R SFG, medial R Cuneus R Middle frontal gyrus R Calcarine fissure and surrounding cortex L SFG, medial L Superior parietal gyrus | 544  261 251 228 223 207 188 182 167 148 144 128 79  69 51 44 39  7 1 | 2961 | < 0.001 |
| 3 | L Crus Cerebellum 1 L Crus Cerebellum 2 L Fusiform gyrus L Lingual gyrus L Cerebellum 6 Vermis 4-5 L Cerebellum 4-5 L Cerebellum 8 R Lingual gyrus L Cerebellum 7b Vermis 6 L Inferior occipital gyrus Vermis 3 R Cerebellum 4-5 OUTSIDE | 246 227 213 178 167 85 56 52 44 33 22 12 6 2 2 | 1345 | < 0.001 |
| 4 | R Middle frontal gyrus R SFG, dorsolateral R SFG, medial R IFG, triangular part R Anterior cingulate and paracingulate gyri | 604 93 73 48 6 | 824 | < 0.001 |
| 5 | L Middle frontal gyrus L IFG, triangular part L SFG, dorsolateral OUTSIDE | 297 61 54 38 | 450 | < 0.001 |
| 6 | R Cerebellum 6 R Fusiform gyrus R Crus Cerebellum 1 R Lingual gyrus | 259 70 22 5 | 356 | < 0.001 |
| 7 | L Thalamus OUTSIDE R Thalamus L Hippocampus | 145 119 20 6 | 290 | < 0.001 |
| 8 | R Paracentral lobule R Precentral gyrus R Postcentral gyrus L Paracentral lobule OUTSIDE | 128 20 14 12 1 | 175 | < 0.001 |
| 9 | L Median cingulate and paracingulate gyri R Median cingulate and paracingulate gyri OUTSIDE L Anterior cingulate and paracingulate gyri | 67  48  27 15 | 157 | 0.002 |
| 10 | R Hippocampus OUTSIDE R Parahippocampal gyrus R Lingual gyrus R Fusiform gyrus | 38 37 31 3 1 | 110 | 0.014 |
| 11 | OUTSIDE L Superior temporal gyrus L Insula L Middle temporal gyrus L Hippocampus | 34 32 28 5 5 | 104 | 0.019 |
| 12 | OUTSIDE R Temporal pole: STG R Temporal pole: MTG R Middle temporal gyrus R Inferior temporal gyrus R Fusiform gyrus | 39 21 19 13 6 1 | 99 | 0.024 |
| 13 | L Precentral gyrus OUTSIDE L Middle frontal gyrus | 40 33 25 | 98 | 0.025 |

*Significance threshold T(21) = 3.53; Cluster extent threshold = 98 voxels.*

**( InfPos > InfNeg ) – positive association with Model Intercept**

| **Cluster** | **Regions** | **Number of voxels in region** | **Cluster size** | **FWE-corrected p-value** |
| --- | --- | --- | --- | --- |
| 1 | R Lingual gyrus R Calcarine fissure and surrounding cortex | 178 6 | 184 | < 0.001 |
| 2 | L Lingual gyrus L Calcarine fissure and surrounding cortex L Middle occipital gyrus L Superior occipital gyrus OUTSIDE | 71 55  7 5 2 | 140 | 0.004 |

*Significance threshold T(21) = 3.53; Cluster extent threshold = 140 voxels.*

**( InfPos > InfNeg ) – negative association with Model Intercept**

| **Cluster** | **Regions** | **Number of voxels in region** | **Cluster size** | **FWE-corrected p-value** |
| --- | --- | --- | --- | --- |
| 1 | L Insula OUTSIDE L Superior temporal gyrus L Rolandic operculum L Putamen L Heschl gyrus L Hippocampus L IFG, opercular part L Parahippocampal gyrus L Amygdala L IFG, orbital part L Postcentral gyrus L Temporal pole: STG L Lingual gyrus L IFG, triangular part L Middle temporal gyrus | 630 252 244 135 114 72 29 21 8 4 4 3 2 2 2 1 | 1523 | < 0.001 |
| 2 | R Insula R Rolandic operculum R Superior temporal gyrus R Heschl gyrus OUTSIDE R IFG, opercular part R Precentral gyrus R Temporal pole: STG R Putamen | 294 275 187 51 45 38 5 2 1 | 898 | < 0.001 |
| 3 | R Amygdala OUTSIDE R Hippocampus R Temporal pole: STG R Putamen R Pallidum R Parahippocampal gyrus | 88 30 21 17 11 1 1 | 169 | 0.001 |

*Significance threshold T(21) = 3.53; Cluster extent threshold = 169 voxels.*

*------------------------------------------------------------------------------------------------------------------------------------------*

***[Model 19:](#Section_5_Table)*** $\left( InfPos > InfNeg \right)= \beta_{0}+ \beta_{1}\left( Prepartum Dep Sx \right)$

*------------------------------------------------------------------------------------------------------------------------------------------*

**( InfPos > InfNeg ) – negative association with Prepartum Depression Symptoms**

| **Cluster** | **Regions** | **Number of voxels in region** | **Cluster size** | **FWE-corrected p-value** |
| --- | --- | --- | --- | --- |
| 1 | OUTSIDE R IFG, orbital part R Middle frontal gyrus R IFG, triangular part R Insula R MFG, orbital part R SFG, medial R SFG, dorsolateral | 447 138 101 63 41 7 1 1 | 799 | < 0.001 |
| 2 | L IFG, triangular part L Middle frontal gyrus OUTSIDE L MFG, orbital part L IFG, orbital part L SFG, orbital part L SFG, dorsolateral | 184 127 83 79 70 5 1 | 549 | < 0.001 |
| 3 | OUTSIDE L Middle temporal gyrus L Inferior temporal gyrus L Hippocampus L Fusiform gyrus | 120 78 65 30 26 | 319 | < 0.001 |
| 4 | L SFG, medial L SFG, dorsolateral L SFG, medial orbital L SFG, orbital part OUTSIDE L Anterior cingulate and paracingulate gyri | 177 55 24 3 3 1 | 263 | < 0.001 |
| 5 | R SFG, medial R SFG, medial orbital R SFG, dorsolateral L SFG, medial orbital | 135 69 26 2 | 232 | < 0.001 |
| 6 | OUTSIDE R Thalamus R Hippocampus R Posterior cingulate gyrus | 135 40 23 1 | 199 | 0.001 |
| 7 | R IFG, opercular part R IFG, triangular part OUTSIDE | 88 45 29 | 162 | 0.003 |
| 8 | OUTSIDE R Angular gyrus R IP, supramarginal and angular gyri R Middle occipital gyrus | 71 57 30  1 | 159 | 0.003 |
| 9 | L Middle temporal gyrus OUTSIDE L Fusiform gyrus L Inferior temporal gyrus L Parahippocampal gyrus L Superior temporal gyrus L Amygdala | 61 32 22 16 15 4 4 | 154 | 0.004 |
| 10 | L Thalamus OUTSIDE L Pallidum | 125 19 1 | 145 | 0.005 |
| 11 | L SFG, dorsolateral L Supplementary motor area OUTSIDE | 88 33 4 | 125 | 0.011 |
| 12 | OUTSIDE L Hippocampus L Putamen L Amygdala L Insula | 80 19 15 4 4 | 122 | 0.013 |
| 13 | OUTSIDE R Hippocampus R Putamen R Parahippocampal gyrus R Pallidum | 56 25 24 4 1 | 110 | 0.021 |
| 14 | OUTSIDE L Superior parietal gyrus L Precuneus L Angular gyrus L Middle occipital gyrus | 83 11 7 1 1 | 103 | 0.028 |
| 15 | OUTSIDE L Precentral gyrus | 95 1 | 96 | 0.039 |

*Significance threshold T(22) = 3.50; Cluster extent threshold = 96 voxels.*

**( InfPos > InfNeg ) – positive association with Model Intercept**

| **Cluster** | **Regions** | **Number of voxels in region** | **Cluster size** | **FWE-corrected p-value** |
| --- | --- | --- | --- | --- |
| 1 | R Lingual gyrus R Calcarine fissure and surrounding cortex | 135 2 | 137 | 0.007 |
| 2 | L Lingual gyrus L Calcarine fissure and surrounding cortex L Superior occipital gyrus L Middle occipital gyrus | 50 38  4 3 | 95 | 0.04 |

*Significance threshold T(22) = 3.50; Cluster extent threshold = 95 voxels.*

**( InfPos > InfNeg ) – negative association with Model Intercept**

| **Cluster** | **Regions** | **Number of voxels in region** | **Cluster size** | **FWE-corrected p-value** |
| --- | --- | --- | --- | --- |
| 1 | L Insula OUTSIDE L Superior temporal gyrus L Putamen L Rolandic operculum L Heschl gyrus L IFG, opercular part L Postcentral gyrus L IFG, orbital part L Pallidum L Amygdala L Middle temporal gyrus | 556 251 161 156 144 80 15 5 5 2 2 1 | 1378 | < 0.001 |
| 2 | R Rolandic operculum R Insula R Superior temporal gyrus OUTSIDE R Putamen R Amygdala R Heschl gyrus R IFG, opercular part R Hippocampus R Temporal pole: STG R Precentral gyrus R Pallidum | 297 277 182 71 67 59 48 44 10 6 5 2 | 1068 | < 0.001 |

*Significance threshold T(22) = 3.50; Cluster extent threshold = 1068 voxels.*

*------------------------------------------------------------------------------------------------------------------------------------------*

***[Model 20:](#Section_5_Table)*** $\left( InfPos > InfNeg \right)= \beta_{0}+ \beta_{1}\left( Prepartum Anx Sx \right)$

*------------------------------------------------------------------------------------------------------------------------------------------*

**( InfPos > InfNeg ) – negative association with Prepartum Anxiety Symptoms**

| **Cluster** | **Regions** | **Number of voxels in region** | **Cluster size** | **FWE-corrected p-value** |
| --- | --- | --- | --- | --- |
| 1 | L Precentral gyrus L Postcentral gyrus L IP, supramarginal and angular gyri OUTSIDE L Superior parietal gyrus L Paracentral lobule | 212 205 178  72 10 1 | 678 | < 0.001 |
| 2 | R Middle frontal gyrus R IFG, opercular part R IFG, triangular part OUTSIDE R Precentral gyrus R SFG, dorsolateral | 169 150 61 12 9 1 | 402 | < 0.001 |
| 3 | R IP, supramarginal and angular gyri R Angular gyrus R Superior parietal gyrus R Postcentral gyrus R Supramarginal gyrus OUTSIDE | 139  70 58 45 25 5 | 342 | < 0.001 |
| 4 | L Middle frontal gyrus OUTSIDE L IFG, opercular part L IFG, triangular part L Precentral gyrus L SFG, dorsolateral | 137 72 54 37 2 1 | 303 | < 0.001 |
| 5 | L IP, supramarginal and angular gyri L Superior parietal gyrus L Angular gyrus L Middle occipital gyrus | 104  90 52 24 | 270 | < 0.001 |
| 6 | R SFG, medial R SFG, dorsolateral L SFG, medial OUTSIDE | 136 55 38 4 | 233 | < 0.001 |
| 7 | L Inferior occipital gyrus L Middle occipital gyrus OUTSIDE L Fusiform gyrus L Lingual gyrus | 82 45 38 13 7 | 185 | 0.001 |
| 8 | R Precentral gyrus OUTSIDE R SFG, dorsolateral R Middle frontal gyrus | 86 50 37 10 | 183 | 0.001 |
| 9 | R Middle frontal gyrus OUTSIDE R IFG, triangular part | 118 23 18 | 159 | 0.003 |
| 10 | L Paracentral lobule L Supplementary motor area OUTSIDE R Paracentral lobule R Supplementary motor area | 104 9 5 2 1 | 121 | 0.014 |
| 11 | R SFG, medial L SFG, medial R Anterior cingulate and paracingulate gyri | 95 19 4 | 118 | 0.015 |
| 12 | R Angular gyrus OUTSIDE R IP, supramarginal and angular gyri R Supramarginal gyrus | 67 22 18  1 | 108 | 0.023 |
| 13 | OUTSIDE R Cerebellum 4-5 R Cerebellum 3 R Parahippocampal gyrus | 62 20 13 1 | 96 | 0.039 |

*Significance threshold T(22) = 3.50; Cluster extent threshold = 96 voxels.*

**( InfPos > InfNeg ) – positive association with Model Intercept**

| **Cluster** | **Regions** | **Number of voxels in region** | **Cluster size** | **FWE-corrected p-value** |
| --- | --- | --- | --- | --- |
| 1 | R Lingual gyrus R Calcarine fissure and surrounding cortex | 149 2 | 151 | 0.004 |
| 2 | L Lingual gyrus L Calcarine fissure and surrounding cortex L Middle occipital gyrus L Superior occipital gyrus | 56 30  3 2 | 91 | 0.048 |

*Significance threshold T(22) = 3.50; Cluster extent threshold = 91 voxels.*

**( InfPos > InfNeg ) – negative association with Model Intercept**

| **Cluster** | **Regions** | **Number of voxels in region** | **Cluster size** | **FWE-corrected p-value** |
| --- | --- | --- | --- | --- |
| 1 | L Insula OUTSIDE L Superior temporal gyrus L Rolandic operculum L Putamen L Heschl gyrus L IFG, opercular part L IFG, orbital part L Postcentral gyrus L Amygdala | 486 195 151 120 113 62 13 7 3 1 | 1151 | < 0.001 |
| 2 | R Rolandic operculum R Insula R Superior temporal gyrus R Heschl gyrus R IFG, opercular part OUTSIDE R Precentral gyrus R Temporal pole: STG R Putamen | 280 250 183 49 43 34 5 3 1 | 848 | < 0.001 |
| 3 | R Amygdala OUTSIDE R Putamen R Hippocampus R Temporal pole: STG | 51 32 30 4 2 | 119 | 0.015 |

*Significance threshold T(22) = 3.50; Cluster extent threshold = 119 voxels.*

*------------------------------------------------------------------------------------------------------------------------------------------*

***[Model 21:](#Section_5_Table)*** $\left( InfPos > InfNeg \right)= \beta_{0}+ \beta_{1}\left( EPDS[T1\text{-}raw] \right)$

*------------------------------------------------------------------------------------------------------------------------------------------*

**( InfPos > InfNeg ) – positive association with Model Intercept**

| **Cluster** | **Regions** | **Number of voxels in region** | **Cluster size** | **FWE-corrected p-value** |
| --- | --- | --- | --- | --- |
| 1 | R Lingual gyrus R Calcarine fissure and surrounding cortex | 143 1 | 144 | 0.009 |

*Significance threshold T(22) = 3.50; Cluster extent threshold = 144 voxels.*

**( InfPos > InfNeg ) – negative association with Model Intercept**

| **Cluster** | **Regions** | **Number of voxels in region** | **Cluster size** | **FWE-corrected p-value** |
| --- | --- | --- | --- | --- |
| 1 | L Insula OUTSIDE L Superior temporal gyrus L Rolandic operculum L Putamen L Heschl gyrus L IFG, opercular part L Postcentral gyrus L Amygdala L IFG, triangular part L Middle temporal gyrus | 427 178 172 135 91 63 18 3 2 2 1 | 1092 | < 0.001 |
| 2 | R Rolandic operculum R Insula R Superior temporal gyrus R Heschl gyrus R IFG, opercular part OUTSIDE R Precentral gyrus R Temporal pole: STG R Putamen | 275 226 188 48 35 25 4 2 1 | 804 | < 0.001 |
| 3 | R Amygdala OUTSIDE R Putamen R Temporal pole: STG R Hippocampus | 54 30 14 6 6 | 110 | 0.031 |

*Significance threshold T(22) = 3.50; Cluster extent threshold = 110 voxels.*

*------------------------------------------------------------------------------------------------------------------------------------------*

***[Model 22:](#Section_5_Table)*** $\left( InfPos > InfNeg \right)= \beta_{0}+ \beta_{1}\left( CESD[T1\text{-}raw] \right)$

*------------------------------------------------------------------------------------------------------------------------------------------*

**( InfPos > InfNeg ) – negative association with CESD Time1 Raw Scores**

| **Cluster** | **Regions** | **Number of voxels in region** | **Cluster size** | **FWE-corrected p-value** |
| --- | --- | --- | --- | --- |
| 1 | R Middle frontal gyrus R IFG, triangular part OUTSIDE R SFG, dorsolateral R IFG, opercular part R SFG, medial R IFG, orbital part R SFG, medial orbital R Insula R MFG, orbital part R Precentral gyrus R SFG, orbital part R Anterior cingulate and paracingulate gyri L SFG, medial L SFG, medial orbital R Rolandic operculum | 882 658 529 287 285 284 210 61 59 19 13 10 3  3 1 1 | 3305 | < 0.001 |
| 2 | OUTSIDE L IP, supramarginal and angular gyri L Thalamus L Angular gyrus R Thalamus L Superior temporal gyrus L Postcentral gyrus L Middle temporal gyrus L Superior parietal gyrus L Precuneus L Middle occipital gyrus L Hippocampus R Hippocampus L Posterior cingulate gyrus L Supramarginal gyrus | 745 235  135 87 66 64 49 43 28 19 19 8 5 3 2 | 1508 | < 0.001 |
| 3 | R IP, supramarginal and angular gyri R Angular gyrus OUTSIDE R Middle occipital gyrus R Supramarginal gyrus R Superior parietal gyrus R Superior temporal gyrus R Superior occipital gyrus | 446  357 246 133 125 16 12 1 | 1336 | < 0.001 |
| 4 | OUTSIDE L Middle frontal gyrus L IFG, triangular part L SFG, medial L IFG, orbital part L SFG, dorsolateral L MFG, orbital part L Anterior cingulate and paracingulate gyri R Caudate nucleus R Anterior cingulate and paracingulate gyri L Insula L Caudate nucleus | 391 290 255 125 79 75 45  32 16 12  11 1 | 1332 | < 0.001 |
| 5 | R SFG, dorsolateral R SFG, medial R Supplementary motor area R Middle frontal gyrus R Precentral gyrus OUTSIDE L SFG, medial | 604 250 187 87 31 20 14 | 1193 | < 0.001 |
| 6 | OUTSIDE L Middle temporal gyrus L Inferior temporal gyrus L Hippocampus L Superior temporal gyrus L Fusiform gyrus L Amygdala L Insula | 261 152 99 56 41 25 10 6 | 650 | < 0.001 |
| 7 | R Precuneus R Superior parietal gyrus R Superior occipital gyrus OUTSIDE | 202 78 24 4 | 308 | < 0.001 |
| 8 | OUTSIDE R Thalamus R Caudate nucleus R Median cingulate and paracingulate gyri | 200 62 25 10 | 297 | < 0.001 |
| 9 | L Precentral gyrus L SFG, dorsolateral L Supplementary motor area L Middle frontal gyrus L Paracentral lobule OUTSIDE | 202 18 14 8 3 2 | 247 | < 0.001 |
| 10 | R Putamen OUTSIDE R Insula R Pallidum | 87 64 61 2 | 214 | < 0.001 |
| 11 | L Precuneus L Superior parietal gyrus | 167 47 | 214 | < 0.001 |
| 12 | L Inferior temporal gyrus OUTSIDE L Fusiform gyrus L Middle temporal gyrus L Hippocampus | 88 47 24 13 1 | 173 | 0.001 |
| 13 | L Crus Cerebellum 1 L Crus Cerebellum 2 L Cerebellum 6 | 143 15 2 | 160 | 0.002 |
| 14 | OUTSIDE R Thalamus | 87 63 | 150 | 0.003 |
| 15 | R Temporal pole: STG R Middle temporal gyrus R Inferior temporal gyrus OUTSIDE R Temporal pole: MTG R Insula | 64 22 17 13 10 6 | 132 | 0.007 |
| 16 | OUTSIDE L Caudate nucleus | 91 24 | 115 | 0.015 |
| 17 | OUTSIDE L Postcentral gyrus L Precentral gyrus | 70 17 11 | 98 | 0.031 |
| 18 | OUTSIDE L Precentral gyrus L IFG, opercular part | 80 5 4 | 89 | 0.046 |

*Significance threshold T(22) = 3.50; Cluster extent threshold = 89 voxels.*

**( InfPos > InfNeg ) – positive association with Model Intercept**

| **Cluster** | **Regions** | **Number of voxels in region** | **Cluster size** | **FWE-corrected p-value** |
| --- | --- | --- | --- | --- |
| 1 | R Lingual gyrus R Calcarine fissure and surrounding cortex | 132 2 | 134 | 0.007 |

*Significance threshold T(22) = 3.50; Cluster extent threshold = 134 voxels.*

**( InfPos > InfNeg ) – negative association with Model Intercept**

| **Cluster** | **Regions** | **Number of voxels in region** | **Cluster size** | **FWE-corrected p-value** |
| --- | --- | --- | --- | --- |
| 1 | L Insula OUTSIDE L Superior temporal gyrus L Putamen L Rolandic operculum L Heschl gyrus L IFG, opercular part L IFG, orbital part L IFG, triangular part L Postcentral gyrus L Middle temporal gyrus L Pallidum L Amygdala | 591 256 197 139 132 73 26 4 4 3 2 2 2 | 1431 | < 0.001 |
| 2 | R Insula R Rolandic operculum R Superior temporal gyrus OUTSIDE R Putamen R IFG, opercular part R Amygdala R Heschl gyrus R Temporal pole: STG R Hippocampus R Precentral gyrus R Pallidum | 316 308 187 104 96 59 52 47 16 6 6 2 | 1199 | < 0.001 |
| Clusters Spanning a Single Region | OUTSIDE |  | 106 | 0.022 |

*Significance threshold T(22) = 3.50; Cluster extent threshold = 106 voxels.*

*------------------------------------------------------------------------------------------------------------------------------------------*

***[Model 23:](#Section_5_Table)*** $\left( InfPos > InfNeg \right)= \beta_{0}+ \beta_{1}\left( BAI[T1\text{-}raw] \right)$

*------------------------------------------------------------------------------------------------------------------------------------------*

**( InfPos > InfNeg ) – negative association with BAI Time1 Raw Scores**

| **Cluster** | **Regions** | **Number of voxels in region** | **Cluster size** | **FWE-corrected p-value** |
| --- | --- | --- | --- | --- |
| 1 | R Middle frontal gyrus R IFG, triangular part R SFG, medial R SFG, dorsolateral R IFG, opercular part OUTSIDE L SFG, medial R Precentral gyrus R SFG, medial orbital R SFG, orbital part R Anterior cingulate and paracingulate gyri R IFG, orbital part | 681 501 375 193 141 84 65 54 12 9 8  5 | 2128 | < 0.001 |
| 2 | L Precentral gyrus L IP, supramarginal and angular gyri L Postcentral gyrus OUTSIDE L Superior parietal gyrus L SFG, dorsolateral L Middle frontal gyrus L Angular gyrus L Supramarginal gyrus L Median cingulate and paracingulate gyri | 355 314  226 180 46 31 14 9 3 2 | 1180 | < 0.001 |
| 3 | L IFG, triangular part L Middle frontal gyrus OUTSIDE L IFG, opercular part L Precentral gyrus L Insula L SFG, dorsolateral | 335 146 124 91 79 4 1 | 780 | < 0.001 |
| 4 | R SFG, medial R SFG, dorsolateral L SFG, medial L Supplementary motor area R Supplementary motor area R Median cingulate and paracingulate gyri OUTSIDE R Anterior cingulate and paracingulate gyri L SFG, dorsolateral | 331 202 147 28 13 11  10 5  4 | 751 | < 0.001 |
| 5 | R IP, supramarginal and angular gyri OUTSIDE R Angular gyrus R Supramarginal gyrus R Superior parietal gyrus R Middle occipital gyrus | 273  180 171 78 10 8 | 720 | < 0.001 |
| 6 | R SFG, dorsolateral R Supplementary motor area R Precentral gyrus R Middle frontal gyrus OUTSIDE | 262 152 106 69 42 | 631 | < 0.001 |
| 7 | OUTSIDE L Middle temporal gyrus L Inferior temporal gyrus L Superior temporal gyrus L Fusiform gyrus L Hippocampus L Insula L Amygdala | 189 175 93 48 30 30 13 1 | 579 | < 0.001 |
| 8 | L Crus Cerebellum 1 L Crus Cerebellum 2 L Cerebellum 8 L Cerebellum 9 L Cerebellum 7b L Cerebellum 6 | 236 120 87 22 19 14 | 498 | < 0.001 |
| 9 | L Angular gyrus L Superior temporal gyrus L Middle temporal gyrus OUTSIDE L Middle occipital gyrus L IP, supramarginal and angular gyri L Supramarginal gyrus | 120 104 72 70 27 9  2 | 404 | < 0.001 |
| 10 | OUTSIDE R Thalamus L Thalamus R Parahippocampal gyrus R Hippocampus R Lingual gyrus | 153 93 82 9 7 2 | 346 | < 0.001 |
| 11 | L Cerebellum 6 L Fusiform gyrus L Cerebellum 4-5 L Lingual gyrus Vermis 4-5 | 105 44 42 24 1 | 216 | < 0.001 |
| 12 | OUTSIDE L Caudate nucleus R Caudate nucleus | 114 40 24 | 178 | 0.001 |
| 13 | R Postcentral gyrus R Superior parietal gyrus R IP, supramarginal and angular gyri R Precentral gyrus | 129 18 8  2 | 157 | 0.002 |
| 14 | OUTSIDE R Thalamus R Caudate nucleus | 97 40 8 | 145 | 0.004 |
| 15 | L Paracentral lobule L Supplementary motor area | 101 20 | 121 | 0.01 |
| 16 | L Middle occipital gyrus L Inferior occipital gyrus OUTSIDE | 105 7 4 | 116 | 0.012 |
| 17 | R Paracentral lobule R Precentral gyrus R Postcentral gyrus | 73 25 14 | 112 | 0.015 |
| 18 | R Insula OUTSIDE R Putamen | 58 37 8 | 103 | 0.022 |
| 19 | L Median cingulate and paracingulate gyri R Median cingulate and paracingulate gyri OUTSIDE L Anterior cingulate and paracingulate gyri | 30  28  27 8 | 93 | 0.035 |
| 20 | R Middle temporal gyrus OUTSIDE R Inferior temporal gyrus | 79 9 2 | 90 | 0.04 |

*Significance threshold T(22) = 3.50; Cluster extent threshold = 90 voxels.*

**( InfPos > InfNeg ) – positive association with Model Intercept**

| **Cluster** | **Regions** | **Number of voxels in region** | **Cluster size** | **FWE-corrected p-value** |
| --- | --- | --- | --- | --- |
| 1 | R Lingual gyrus R Calcarine fissure and surrounding cortex | 154 5 | 159 | 0.002 |

*Significance threshold T(22) = 3.50; Cluster extent threshold = 159 voxels.*

**( InfPos > InfNeg ) – negative association with Model Intercept**

| **Cluster** | **Regions** | **Number of voxels in region** | **Cluster size** | **FWE-corrected p-value** |
| --- | --- | --- | --- | --- |
| 1 | L Insula OUTSIDE L Superior temporal gyrus L Rolandic operculum L Putamen L Heschl gyrus L IFG, opercular part L Postcentral gyrus L IFG, triangular part L Middle temporal gyrus L Amygdala | 512 228 204 138 134 69 19 4 3 2 1 | 1314 | < 0.001 |
| 2 | R Rolandic operculum R Insula R Superior temporal gyrus OUTSIDE R Putamen R IFG, opercular part R Heschl gyrus R Amygdala R Precentral gyrus R Temporal pole: STG R Hippocampus R Pallidum | 321 289 191 93 64 49 47 44 7 6 3 1 | 1115 | < 0.001 |

*Significance threshold T(22) = 3.50; Cluster extent threshold = 1115 voxels.*

*------------------------------------------------------------------------------------------------------------------------------------------*

***[Model 24:](#Section_5_Table)*** $\left( InfPos > InfNeg \right)= \beta_{0}+ \beta_{1}\left( CESD[T4\text{-}raw] \right)$

*------------------------------------------------------------------------------------------------------------------------------------------*

**( InfPos > InfNeg ) – positive association with Model Intercept**

| **Cluster** | **Regions** | **Number of voxels in region** | **Cluster size** | **FWE-corrected p-value** |
| --- | --- | --- | --- | --- |
| Clusters Spanning a Single Region | R Lingual gyrus |  | 83 | 0.031 |

*Significance threshold T(13) = 3.85; Cluster extent threshold = 83 voxels.*

**( InfPos > InfNeg ) – negative association with Model Intercept**

| **Cluster** | **Regions** | **Number of voxels in region** | **Cluster size** | **FWE-corrected p-value** |
| --- | --- | --- | --- | --- |
| 1 | R Amygdala R Hippocampus OUTSIDE R Temporal pole: STG R Parahippocampal gyrus | 52 44 2 1 1 | 100 | 0.012 |
| 2 | R Superior temporal gyrus R Heschl gyrus R Insula | 74 4 2 | 80 | 0.036 |

*Significance threshold T(13) = 3.85; Cluster extent threshold = 80 voxels.*

*------------------------------------------------------------------------------------------------------------------------------------------*

***[Model 25:](#Section_5_Table)*** $\left( InfPos > InfNeg \right)= \beta_{0}+ \beta_{1}\left( BAI[T4\text{-}raw] \right)$

*------------------------------------------------------------------------------------------------------------------------------------------*

**( InfPos > InfNeg ) – negative association with BAI Time4 Raw Scores**

| **Cluster** | **Regions** | **Number of voxels in region** | **Cluster size** | **FWE-corrected p-value** |
| --- | --- | --- | --- | --- |
| 1 | R Lingual gyrus R Cerebellum 6 Vermis 6 R Cerebellum 4-5 | 65 42 15 2 | 124 | 0.002 |
| 2 | R Superior parietal gyrus R IP, supramarginal and angular gyri | 59 38 | 97 | 0.009 |
| 3 | R Median cingulate and paracingulate gyri L Median cingulate and paracingulate gyri OUTSIDE R Posterior cingulate gyrus L Posterior cingulate gyrus | 54  17  15 4 1 | 91 | 0.012 |
| 4 | R Fusiform gyrus R Inferior occipital gyrus R Crus Cerebellum 1 R Lingual gyrus R Cerebellum 6 | 42 25 6 3 1 | 77 | 0.029 |
| 5 | R Posterior cingulate gyrus OUTSIDE L Posterior cingulate gyrus R Precuneus | 30 22 12 4 | 68 | 0.05 |

*Significance threshold T(13) = 3.85; Cluster extent threshold = 68 voxels.*

**( InfPos > InfNeg ) – positive association with Model Intercept**

| **Cluster** | **Regions** | **Number of voxels in region** | **Cluster size** | **FWE-corrected p-value** |
| --- | --- | --- | --- | --- |
| Clusters Spanning a Single Region | R Lingual gyrus |  | 145 | < 0.001 |

*Significance threshold T(13) = 3.85; Cluster extent threshold = 145 voxels.*

**( InfPos > InfNeg ) – negative association with Model Intercept**

| **Cluster** | **Regions** | **Number of voxels in region** | **Cluster size** | **FWE-corrected p-value** |
| --- | --- | --- | --- | --- |
| 1 | L Superior temporal gyrus L Heschl gyrus L Rolandic operculum L Insula | 36 22 7 5 | 70 | 0.044 |

*Significance threshold T(13) = 3.85; Cluster extent threshold = 70 voxels.*

*------------------------------------------------------------------------------------------------------------------------------------------*

***[Model 26:](#Section_5_Table)*** $\left( InfPos > InfNeg \right)= \beta_{0}+ \beta_{1}\left( CESD[T1\text{-raw}] \right) + \beta_{2}\left( CESD[T4\text{-}raw] \right)$

*------------------------------------------------------------------------------------------------------------------------------------------*

**( InfPos > InfNeg ) – negative association with Model Intercept**

| **Cluster** | **Regions** | **Number of voxels in region** | **Cluster size** | **FWE-corrected p-value** |
| --- | --- | --- | --- | --- |
| 1 | R Amygdala R Hippocampus R Temporal pole: STG OUTSIDE | 59 45 6 1 | 111 | 0.006 |

*Significance threshold T(12) = 3.93; Cluster extent threshold = 111 voxels.*

*------------------------------------------------------------------------------------------------------------------------------------------*

***[Model 27:](#Section_5_Table)*** $\left( InfPos > InfNeg \right)= \beta_{0}+ \beta_{1}\left( BAI[T1\text{-raw}] \right)+ \beta_{2}\left( BAI[T4\text{-raw}] \right)$

*------------------------------------------------------------------------------------------------------------------------------------------*

**( InfPos > InfNeg ) – positive association with BAI Time1 Raw Scores**

| **Cluster** | **Regions** | **Number of voxels in region** | **Cluster size** | **FWE-corrected p-value** |
| --- | --- | --- | --- | --- |
| 1 | R IFG, triangular part R Middle frontal gyrus R IFG, opercular part R Precentral gyrus OUTSIDE R Rolandic operculum R Insula | 255 107 90 47 45 20 9 | 573 | < 0.001 |
| 2 | L Postcentral gyrus L Precentral gyrus L IP, supramarginal and angular gyri L Superior parietal gyrus L SFG, dorsolateral OUTSIDE | 211 198 45  13 12 2 | 481 | < 0.001 |
| 3 | L Precentral gyrus L IFG, triangular part L IFG, opercular part OUTSIDE L Middle frontal gyrus | 72 57 54 33 1 | 217 | < 0.001 |
| 4 | L Cerebellum 6 L Fusiform gyrus L Cerebellum 4-5 L Lingual gyrus | 90 68 46 3 | 207 | < 0.001 |
| 5 | L SFG, medial R SFG, medial OUTSIDE | 82 54 1 | 137 | < 0.001 |
| 6 | R Supplementary motor area OUTSIDE R SFG, dorsolateral R Precentral gyrus | 43 31 28 1 | 103 | 0.002 |
| 7 | R SFG, medial R SFG, dorsolateral L SFG, medial OUTSIDE | 61 15 1 1 | 78 | 0.012 |
| 8 | R Superior temporal gyrus R Middle temporal gyrus OUTSIDE | 46 19 5 | 70 | 0.021 |
| 9 | R Postcentral gyrus R Superior parietal gyrus | 52 7 | 59 | 0.047 |

*Significance threshold T(12) = 3.93; Cluster extent threshold = 59 voxels.*

**( InfPos > InfNeg ) – negative association with BAI Time1 Raw Scores**

| **Cluster** | **Regions** | **Number of voxels in region** | **Cluster size** | **FWE-corrected p-value** |
| --- | --- | --- | --- | --- |
| 1 | R IFG, triangular part R Middle frontal gyrus R IFG, opercular part R Precentral gyrus OUTSIDE R Rolandic operculum R Insula | 255 107 90 47 45 20 9 | 573 | < 0.001 |
| 2 | L Postcentral gyrus L Precentral gyrus L IP, supramarginal and angular gyri L Superior parietal gyrus L SFG, dorsolateral OUTSIDE | 211 198 45  13 12 2 | 481 | < 0.001 |
| 3 | L Precentral gyrus L IFG, triangular part L IFG, opercular part OUTSIDE L Middle frontal gyrus | 72 57 54 33 1 | 217 | < 0.001 |
| 4 | L Cerebellum 6 L Fusiform gyrus L Cerebellum 4-5 L Lingual gyrus | 90 68 46 3 | 207 | < 0.001 |
| 5 | L SFG, medial R SFG, medial OUTSIDE | 82 54 1 | 137 | < 0.001 |
| 6 | R Supplementary motor area OUTSIDE R SFG, dorsolateral R Precentral gyrus | 43 31 28 1 | 103 | 0.002 |
| 7 | R SFG, medial R SFG, dorsolateral L SFG, medial OUTSIDE | 61 15 1 1 | 78 | 0.012 |
| 8 | R Superior temporal gyrus R Middle temporal gyrus OUTSIDE | 46 19 5 | 70 | 0.021 |
| 9 | R Postcentral gyrus R Superior parietal gyrus | 52 7 | 59 | 0.047 |

*Significance threshold T(12) = 3.93; Cluster extent threshold = 59 voxels.*

**( InfPos > InfNeg ) – negative association with BAI Time4 Raw Scores**

| **Cluster** | **Regions** | **Number of voxels in region** | **Cluster size** | **FWE-corrected p-value** |
| --- | --- | --- | --- | --- |
| 1 | R Fusiform gyrus R Inferior occipital gyrus R Crus Cerebellum 1 R Lingual gyrus R Cerebellum 6 | 34 25 5 3 1 | 68 | 0.024 |
| 2 | R Lingual gyrus R Cerebellum 6 Vermis 6 | 33 22 5 | 60 | 0.043 |

*Significance threshold T(12) = 3.93; Cluster extent threshold = 60 voxels.*

**( InfPos > InfNeg ) – positive association with Model Intercept**

| **Cluster** | **Regions** | **Number of voxels in region** | **Cluster size** | **FWE-corrected p-value** |
| --- | --- | --- | --- | --- |
| 1 | R Lingual gyrus R Calcarine fissure and surrounding cortex | 141 2 | 143 | < 0.001 |

*Significance threshold T(12) = 3.93; Cluster extent threshold = 143 voxels.*

**( InfPos > InfNeg ) – negative association with Model Intercept**

| **Cluster** | **Regions** | **Number of voxels in region** | **Cluster size** | **FWE-corrected p-value** |
| --- | --- | --- | --- | --- |
| 1 | R Precuneus R Cuneus R Calcarine fissure and surrounding cortex R Superior occipital gyrus | 56 52 18  1 | 127 | 0.001 |
| 2 | L Insula OUTSIDE L Rolandic operculum L Putamen L Heschl gyrus | 41 26 9 7 1 | 84 | 0.008 |
| 3 | L Superior temporal gyrus L Heschl gyrus L Rolandic operculum L Insula | 29 19 6 5 | 59 | 0.047 |

*Significance threshold T(12) = 3.93; Cluster extent threshold = 59 voxels.*

*------------------------------------------------------------------------------------------------------------------------------------------*

***[Model 28:](#Section_5_Table)*** $\left( InfPos > InfNeg \right)= \beta_{0}+ \beta_{1}\left( Dep/Anx Hx \right)$

*------------------------------------------------------------------------------------------------------------------------------------------*

**( InfPos > InfNeg ) – negative association with Model Intercept**

| **Cluster** | **Regions** | **Number of voxels in region** | **Cluster size** | **FWE-corrected p-value** |
| --- | --- | --- | --- | --- |
| 1 | L Insula OUTSIDE L Putamen L Superior temporal gyrus L Rolandic operculum L Heschl gyrus L Olfactory cortex | 148 123 77 40 35 24 2 | 449 | < 0.001 |
| 2 | R Rolandic operculum R Superior temporal gyrus R Insula R Heschl gyrus OUTSIDE R IFG, opercular part R Temporal pole: STG | 139 96 55 25 18 9 1 | 343 | < 0.001 |
| 3 | R Putamen OUTSIDE R Amygdala | 67 32 5 | 104 | 0.039 |

*Significance threshold T(22) = 3.50; Cluster extent threshold = 104 voxels.*

*------------------------------------------------------------------------------------------------------------------------------------------*

***[Model 29:](#Section_5_Table)*** $\left( InfPos > InfNeg \right)= \beta_{0}+ \beta_{1}\left( Dep/Anx Dx \right)$

*------------------------------------------------------------------------------------------------------------------------------------------*

**( InfPos > InfNeg ) – negative association with Current Dep/Anx Diagnosis**

| **Cluster** | **Regions** | **Number of voxels in region** | **Cluster size** | **FWE-corrected p-value** |
| --- | --- | --- | --- | --- |
| 1 | R IFG, triangular part R IFG, opercular part OUTSIDE R Middle frontal gyrus R Precentral gyrus R Postcentral gyrus R Insula R IFG, orbital part R Supramarginal gyrus R Rolandic operculum | 619 488 438 313 222 179 9 9 6 3 | 2286 | < 0.001 |
| 2 | L Precentral gyrus L Postcentral gyrus L IFG, triangular part L IFG, opercular part OUTSIDE L Middle frontal gyrus L Rolandic operculum | 290 258 124 116 109 89 2 | 988 | < 0.001 |
| 3 | R Precentral gyrus R SFG, dorsolateral R Middle frontal gyrus OUTSIDE R Supplementary motor area | 296 155 102 45 44 | 642 | < 0.001 |
| 4 | R Putamen OUTSIDE R Insula R Pallidum R IFG, orbital part R SFG, orbital part R Gyrus rectus | 115 102 86 38 13 7 6 | 367 | < 0.001 |
| 5 | L Middle temporal gyrus L Superior temporal gyrus OUTSIDE | 202 31 1 | 234 | < 0.001 |
| 6 | R Middle occipital gyrus R Middle temporal gyrus OUTSIDE | 125 70 4 | 199 | < 0.001 |
| 7 | R Middle temporal gyrus OUTSIDE R Superior temporal gyrus | 85 82 12 | 179 | 0.001 |
| 8 | R Middle temporal gyrus R Superior temporal gyrus OUTSIDE | 70 54 36 | 160 | 0.002 |
| 9 | OUTSIDE R Thalamus | 100 60 | 160 | 0.002 |
| 10 | L Putamen OUTSIDE L Insula | 106 28 10 | 144 | 0.004 |
| 11 | L Lingual gyrus OUTSIDE L Fusiform gyrus L Calcarine fissure and surrounding cortex | 113 23 2 1 | 139 | 0.005 |
| 12 | R Superior parietal gyrus R Superior occipital gyrus R Cuneus R Precuneus | 65 30 26 17 | 138 | 0.005 |
| 13 | R IP, supramarginal and angular gyri R Superior parietal gyrus R Postcentral gyrus OUTSIDE R Angular gyrus | 44  42 41 5 2 | 134 | 0.006 |
| 14 | R SFG, medial L SFG, medial R SFG, medial orbital OUTSIDE L Anterior cingulate and paracingulate gyri | 58 38 17 2 1 | 116 | 0.013 |
| 15 | L IFG, orbital part L Insula OUTSIDE L SFG, orbital part L MFG, orbital part | 38 33 18 14 11 | 114 | 0.014 |
| 16 | L Superior occipital gyrus OUTSIDE L Middle occipital gyrus L Cuneus L Calcarine fissure and surrounding cortex | 82 16 8 5 1 | 112 | 0.015 |
| 17 | L Caudate nucleus OUTSIDE | 53 39 | 92 | 0.038 |
| 18 | L Postcentral gyrus OUTSIDE L Precentral gyrus L IP, supramarginal and angular gyri | 71 12 8 1 | 92 | 0.038 |
| 19 | R Supplementary motor area R SFG, medial R SFG, dorsolateral | 37 35 19 | 91 | 0.04 |
| Clusters Spanning a Single Region | L IFG, triangular part |  | 189 | 0.001 |

*Significance threshold T(22) = 3.50; Cluster extent threshold = 91 voxels.*

**( InfPos > InfNeg ) – positive association with Model Intercept**

| **Cluster** | **Regions** | **Number of voxels in region** | **Cluster size** | **FWE-corrected p-value** |
| --- | --- | --- | --- | --- |
| 1 | L Lingual gyrus R Lingual gyrus L Calcarine fissure and surrounding cortex L Cerebellum 6 L Middle occipital gyrus L Crus Cerebellum 1 L Fusiform gyrus L Superior occipital gyrus R Calcarine fissure and surrounding cortex OUTSIDE R Cuneus | 266 160 68  28 21 15 15 10 8  5 1 | 597 | < 0.001 |
| 2 | R Middle temporal gyrus R Superior temporal gyrus R Middle occipital gyrus OUTSIDE R Inferior occipital gyrus R Inferior temporal gyrus | 297 121 94 31 20 6 | 569 | < 0.001 |
| 3 | L Middle occipital gyrus L Middle temporal gyrus L Inferior occipital gyrus OUTSIDE | 268 158 2 1 | 429 | < 0.001 |
| 4 | R IFG, opercular part R Precentral gyrus R Middle frontal gyrus R IFG, triangular part OUTSIDE | 104 29 26 4 1 | 164 | 0.002 |

*Significance threshold T(22) = 3.50; Cluster extent threshold = 164 voxels.*

**( InfPos > InfNeg ) – negative association with Model Intercept**

| **Cluster** | **Regions** | **Number of voxels in region** | **Cluster size** | **FWE-corrected p-value** |
| --- | --- | --- | --- | --- |
| 1 | L Insula L Superior temporal gyrus OUTSIDE L Rolandic operculum L Heschl gyrus L Putamen | 135 39 28 27 19 4 | 252 | < 0.001 |
| 2 | R Superior temporal gyrus R Rolandic operculum R Insula R Heschl gyrus R IFG, opercular part OUTSIDE | 88 63 56 17 1 1 | 226 | < 0.001 |

*Significance threshold T(22) = 3.50; Cluster extent threshold = 226 voxels.*

*------------------------------------------------------------------------------------------------------------------------------------------*

***[Model 30:](#Section_5_Table)*** $\left( InfPos > InfNeg \right)= \beta_{0}+ \beta_{1}\left( Dep Dose \right)+ \beta_{2}\left( Anx Dose \right)$

*------------------------------------------------------------------------------------------------------------------------------------------*

**( InfPos > InfNeg ) – negative association with Anxiety "Dose"**

| **Cluster** | **Regions** | **Number of voxels in region** | **Cluster size** | **FWE-corrected p-value** |
| --- | --- | --- | --- | --- |
| 1 | R Precentral gyrus R Middle frontal gyrus OUTSIDE R SFG, dorsolateral | 76 46 31 2 | 155 | 0.003 |
| 2 | L Lingual gyrus OUTSIDE L Calcarine fissure and surrounding cortex L Fusiform gyrus L Middle occipital gyrus | 60 46 10  8 3 | 127 | 0.01 |
| Clusters Spanning a Single Region | OUTSIDE |  | 169 | 0.002 |

*Significance threshold T(12) = 3.53; Cluster extent threshold = 127 voxels.*

**( InfPos > InfNeg ) – positive association with Model Intercept**

| **Cluster** | **Regions** | **Number of voxels in region** | **Cluster size** | **FWE-corrected p-value** |
| --- | --- | --- | --- | --- |
| 1 | L Middle occipital gyrus L Middle temporal gyrus OUTSIDE | 232 92 6 | 330 | < 0.001 |
| 2 | R Middle temporal gyrus R Middle occipital gyrus R Inferior occipital gyrus R Inferior temporal gyrus | 113 45 11 5 | 174 | 0.002 |
| 3 | R Lingual gyrus L Lingual gyrus R Calcarine fissure and surrounding cortex L Calcarine fissure and surrounding cortex | 106 22 1  1 | 130 | 0.009 |

*Significance threshold T(21) = 3.53; Cluster extent threshold = 130 voxels.*

**( InfPos > InfNeg ) – negative association with Model Intercept**

| **Cluster** | **Regions** | **Number of voxels in region** | **Cluster size** | **FWE-corrected p-value** |
| --- | --- | --- | --- | --- |
| 1 | R Superior temporal gyrus R Insula R Heschl gyrus | 68 32 7 | 107 | 0.023 |

*Significance threshold T(21) = 3.53; Cluster extent threshold = 107 voxels.*

*------------------------------------------------------------------------------------------------------------------------------------------*

***[Model 31:](#Section_5_Table)*** $\left( InfPos > InfNeg \right)= \beta_{0}+ \beta_{1}\left( Cortisol[T4\text{-}linear\text{-term} \right)$

$+ \beta_{2}\left( Cortisol[T4\text{-}intercept \right)$

*------------------------------------------------------------------------------------------------------------------------------------------*

**( InfPos > InfNeg ) – positive association with Model Intercept**

| **Cluster** | **Regions** | **Number of voxels in region** | **Cluster size** | **FWE-corrected p-value** |
| --- | --- | --- | --- | --- |
| 1 | R Lingual gyrus R Calcarine fissure and surrounding cortex | 168 1 | 169 | 0.003 |
| 2 | L Lingual gyrus L Calcarine fissure and surrounding cortex L Middle occipital gyrus L Superior occipital gyrus | 77 29  2 2 | 110 | 0.03 |

*Significance threshold T(21) = 3.53; Cluster extent threshold = 110 voxels.*

**( InfPos > InfNeg ) – negative association with Model Intercept**

| **Cluster** | **Regions** | **Number of voxels in region** | **Cluster size** | **FWE-corrected p-value** |
| --- | --- | --- | --- | --- |
| 1 | L Insula L Superior temporal gyrus OUTSIDE L Rolandic operculum L Putamen L Heschl gyrus L IFG, opercular part L Amygdala L Postcentral gyrus L Middle temporal gyrus | 387 168 163 123 75 64 8 4 3 1 | 996 | < 0.001 |
| 2 | R Rolandic operculum R Insula R Superior temporal gyrus R Heschl gyrus R IFG, opercular part OUTSIDE R Temporal pole: STG R Precentral gyrus R Putamen | 318 225 196 54 41 23 7 5 1 | 870 | < 0.001 |

*Significance threshold T(21) = 3.53; Cluster extent threshold = 870 voxels.*

**[Section V.D.](#Table_of_Contents)** Regression against (InfOwnPos > InfOtherPos)

*------------------------------------------------------------------------------------------------------------------------------------------*

***[Model 32:](#Section_5_Table)*** $\left( InfOwnPos > InfOtherPos \right)= \beta_{0}+ \beta_{1}\left( CESD[T1\text{-}4] \right)$

*------------------------------------------------------------------------------------------------------------------------------------------*

**( InfOwnPos > InfOtherPos ) – positive association with CESD[T1-4]**

| **Cluster** | **Regions** | **Number of voxels in region** | **Cluster size** | **FWE-corrected p-value** |
| --- | --- | --- | --- | --- |
| 1 | R Cerebellum 6 OUTSIDE L Middle temporal gyrus L Cerebellum 6 L Crus Cerebellum 1 R Crus Cerebellum 1 R Cerebellum 8 L Superior temporal gyrus L Cerebellum 9 L Lingual gyrus Vermis 6 L Hippocampus L Fusiform gyrus R Cerebellum 4-5 L Cerebellum 8 Vermis 7 L Inferior temporal gyrus R Lingual gyrus Vermis 8 L Supramarginal gyrus L Inferior occipital gyrus Vermis 9 Vermis 4-5 R Crus Cerebellum 2 L Angular gyrus R Fusiform gyrus L Crus Cerebellum 2 L Cerebellum 4-5 R Cerebellum 9 L Calcarine fissure and surrounding cortex Vermis 9 R Cerebellum 10 L Middle occipital gyrus R Cerebellum 7b L Amygdala | 1144 1103 826 706 619 515 352 345 279 268 246 199 192 160 152 135 122 109 103 102 83 73 69 65 51 49 35 28 24 21  15 13 10 6 2 | 8221 | < 0.001 |
| 2 | R Superior temporal gyrus R Temporal pole: STG R Middle temporal gyrus OUTSIDE R Insula | 284 27 26 11 2 | 350 | < 0.001 |
| 3 | OUTSIDE L Thalamus | 191 17 | 208 | 0.001 |
| 4 | L Middle frontal gyrus L IFG, triangular part L IFG, opercular part OUTSIDE L Precentral gyrus | 50 46 23 16 6 | 141 | 0.007 |
| 5 | OUTSIDE R Hippocampus R Amygdala R Pallidum | 84 29 12 3 | 128 | 0.012 |
| 6 | R Insula R IFG, triangular part OUTSIDE | 47 45 27 | 119 | 0.017 |
| 7 | L Angular gyrus L IP, supramarginal and angular gyri L Superior parietal gyrus | 51 46  7 | 104 | 0.031 |
| 8 | L Caudate nucleus OUTSIDE | 78 24 | 102 | 0.034 |
| 9 | OUTSIDE R Parahippocampal gyrus R Hippocampus R Lingual gyrus | 42 22 19 12 | 95 | 0.046 |

*Significance threshold T(22) = 3.50; Cluster extent threshold = 95 voxels.*

**( InfOwnPos > InfOtherPos ) – negative association with CESD[T1-4]**

| **Cluster** | **Regions** | **Number of voxels in region** | **Cluster size** | **FWE-corrected p-value** |
| --- | --- | --- | --- | --- |
| 1 | L Paracentral lobule R Postcentral gyrus R Precentral gyrus R Supplementary motor area L Precentral gyrus R SFG, dorsolateral R Superior parietal gyrus R Paracentral lobule L Supplementary motor area L SFG, dorsolateral L Postcentral gyrus OUTSIDE L Superior parietal gyrus L Precuneus R IP, supramarginal and angular gyri R Middle frontal gyrus L Middle frontal gyrus R Precuneus | 472 363 342 333 319 254 173 172 169 167 156 75 71 19 11  9 7 1 | 3113 | < 0.001 |
| 2 | OUTSIDE L Postcentral gyrus L IP, supramarginal and angular gyri | 164 11 5 | 180 | 0.002 |
| 3 | R SFG, dorsolateral R Middle frontal gyrus OUTSIDE | 56 37 4 | 97 | 0.042 |

*Significance threshold T(22) = 3.50; Cluster extent threshold = 97 voxels.*

**( InfOwnPos > InfOtherPos ) – positive association with Model Intercept**

| **Cluster** | **Regions** | **Number of voxels in region** | **Cluster size** | **FWE-corrected p-value** |
| --- | --- | --- | --- | --- |
| 1 | L Caudate nucleus OUTSIDE R Olfactory cortex R Anterior cingulate and paracingulate gyri L Olfactory cortex L Anterior cingulate and paracingulate gyri | 89 68 21 14  11 1 | 204 | 0.001 |

*Significance threshold T(22) = 3.50; Cluster extent threshold = 204 voxels.*

*------------------------------------------------------------------------------------------------------------------------------------------*

***[Model 33:](#Section_5_Table)*** $\left( InfOwnPos > InfOtherPos \right)= \beta_{0}+ \beta_{1}\left( BAI[T1\text{-}4] \right)$

*------------------------------------------------------------------------------------------------------------------------------------------*

**( InfOwnPos > InfOtherPos ) – positive association with BAI[T1-4]**

| **Cluster** | **Regions** | **Number of voxels in region** | **Cluster size** | **FWE-corrected p-value** |
| --- | --- | --- | --- | --- |
| 1 | R IFG, triangular part R Insula OUTSIDE R IFG, opercular part R IFG, orbital part | 156 120 46 31 11 | 364 | < 0.001 |
| 2 | R Cerebellum 6 R Crus Cerebellum 2 R Crus Cerebellum 1 R Cerebellum 4-5 OUTSIDE R Cerebellum 8 Vermis 7 Vermis 4-5 | 212 35 22 14 14 12 6 1 | 316 | < 0.001 |
| 3 | L Middle temporal gyrus L Superior temporal gyrus OUTSIDE | 160 41 18 | 219 | 0.001 |
| 4 | OUTSIDE L Lingual gyrus L Calcarine fissure and surrounding cortex | 68 59 9 | 136 | 0.010 |
| 5 | R Superior temporal gyrus R Temporal pole: STG R Middle temporal gyrus | 86 24 1 | 111 | 0.025 |
| 6 | L Middle temporal gyrus L Superior temporal gyrus OUTSIDE | 75 21 5 | 101 | 0.038 |

*Significance threshold T(22) = 3.50; Cluster extent threshold = 101 voxels.*

**( InfOwnPos > InfOtherPos ) – negative association with BAI[T1-4]**

| **Cluster** | **Regions** | **Number of voxels in region** | **Cluster size** | **FWE-corrected p-value** |
| --- | --- | --- | --- | --- |
| 1 | R Postcentral gyrus L Paracentral lobule R Superior parietal gyrus L Superior parietal gyrus R Precentral gyrus L Precuneus L Precentral gyrus R Supplementary motor area R Paracentral lobule R SFG, dorsolateral OUTSIDE R Precuneus L Postcentral gyrus L Supplementary motor area L SFG, dorsolateral R Angular gyrus R IP, supramarginal and angular gyri R Superior occipital gyrus L IP, supramarginal and angular gyri R Median cingulate and paracingulate gyri R Middle occipital gyrus L Middle frontal gyrus R Middle frontal gyrus R Supramarginal gyrus | 737 571 439 408 362 356 332 317 279 276 267 244 207 202 119 80 57  41 13  12  4 4 3 1 | 5331 | < 0.001 |
| 2 | OUTSIDE L IP, supramarginal and angular gyri L Postcentral gyrus L Median cingulate and paracingulate gyri | 256 13  4 4 | 277 | < 0.001 |
| 3 | L SFG, medial orbital OUTSIDE L Anterior cingulate and paracingulate gyri R SFG, medial orbital L SFG, orbital part L Gyrus rectus | 45 26 19  11 4 2 | 107 | 0.030 |

*Significance threshold T(22) = 3.50; Cluster extent threshold = 107 voxels.*

**( InfOwnPos > InfOtherPos ) – positive association with Model Intercept**

| **Cluster** | **Regions** | **Number of voxels in region** | **Cluster size** | **FWE-corrected p-value** |
| --- | --- | --- | --- | --- |
| 1 | L Caudate nucleus OUTSIDE R Olfactory cortex R Anterior cingulate and paracingulate gyri L Olfactory cortex L Anterior cingulate and paracingulate gyri | 71 68 20 15  15 1 | 190 | 0.001 |

*Significance threshold T(22) = 3.50; Cluster extent threshold = 190 voxels.*

*------------------------------------------------------------------------------------------------------------------------------------------*

***[Model 34:](#Section_5_Table)*** $\left( InfOwnPos > InfOtherPos \right)= \beta_{0}+ \beta_{1}\left( Dep Hx \right) + \beta_{2}\left( Dep Dx \right)$

*------------------------------------------------------------------------------------------------------------------------------------------*

**( InfOwnPos > InfOtherPos ) – positive association with Dep Dx**

| **Cluster** | **Regions** | **Number of voxels in region** | **Cluster size** | **FWE-corrected p-value** |
| --- | --- | --- | --- | --- |
| 1 | L Middle temporal gyrus L Superior temporal gyrus OUTSIDE | 171 10 3 | 184 | 0.002 |
| 2 | L Lingual gyrus OUTSIDE L Fusiform gyrus | 103 61 8 | 172 | 0.003 |
| 3 | OUTSIDE L Inferior temporal gyrus L Fusiform gyrus | 62 26 7 | 95 | 0.049 |

*Significance threshold T(21) = 3.53; Cluster extent threshold = 95 voxels.*

**( InfOwnPos > InfOtherPos ) – negative association with Dep Dx**

| **Cluster** | **Regions** | **Number of voxels in region** | **Cluster size** | **FWE-corrected p-value** |
| --- | --- | --- | --- | --- |
| 1 | R Postcentral gyrus R SFG, dorsolateral L Postcentral gyrus R Precentral gyrus L Precentral gyrus L Paracentral lobule OUTSIDE L Precuneus L Superior parietal gyrus R Middle frontal gyrus R SFG, medial R Superior parietal gyrus L SFG, medial R Paracentral lobule L SFG, dorsolateral R Supplementary motor area L IP, supramarginal and angular gyri R IP, supramarginal and angular gyri L Supplementary motor area R Precuneus L Middle frontal gyrus R Supramarginal gyrus R IFG, opercular part L Median cingulate and paracingulate gyri | 650 640 513 504 464 351 339 328 300 296 220 210 205 154 107 88 74  64  31 12 9 6 5 2 | 5572 | < 0.001 |
| 2 | L Middle occipital gyrus L Calcarine fissure and surrounding cortex L Inferior occipital gyrus L Superior occipital gyrus L Lingual gyrus L Cuneus | 107 56  45 20 3 1 | 232 | < 0.001 |
| 3 | R Precuneus R Superior occipital gyrus R Superior parietal gyrus OUTSIDE L Precuneus | 124 42 24 23 7 | 220 | 0.001 |
| 4 | L Parahippocampal gyrus L Fusiform gyrus L Hippocampus OUTSIDE L Cerebellum 4-5 | 89 17 12 9 4 | 131 | 0.012 |

*Significance threshold T(21) = 3.53; Cluster extent threshold = 131 voxels.*

*------------------------------------------------------------------------------------------------------------------------------------------*

***[Model 35:](#Section_5_Table)*** $\left( InfOwnPos > InfOtherPos \right)= \beta_{0}+ \beta_{1}\left( Anx Hx \right)+ \beta_{2}\left( Anx Dx \right)$

*------------------------------------------------------------------------------------------------------------------------------------------*

**( InfOwnPos > InfOtherPos ) – positive association with Anx Hx**

| **Cluster** | **Regions** | **Number of voxels in region** | **Cluster size** | **FWE-corrected p-value** |
| --- | --- | --- | --- | --- |
| 1 | L Cerebellum 8 R Cerebellum 6 L Cerebellum 9 L Crus Cerebellum 1 OUTSIDE R Cerebellum 4-5 Vermis 6 L Crus Cerebellum 2 L Cerebellum 6 Vermis 7 Vermis 8 L Cerebellum 7b Vermis 4-5 R Lingual gyrus Vermis 9 | 205 161 145 85 84 77 74 73 39 36 32 21 7 4 1 | 1044 | < 0.001 |
| 2 | R Cerebellum 8 OUTSIDE R Cerebellum 6 R Crus Cerebellum 1 R Crus Cerebellum 2 R Cerebellum 10 R Cerebellum 9 R Cerebellum 7b | 279 55 38 23 7 1 1 1 | 405 | < 0.001 |
| 3 | OUTSIDE R Thalamus Vermis 3 R Lingual gyrus L Thalamus R Hippocampus R Precuneus R Posterior cingulate gyrus | 249 50 30 30 27 6 2 1 | 395 | < 0.001 |
| 4 | OUTSIDE R Caudate nucleus | 152 7 | 159 | 0.004 |
| 5 | R Cerebellum 9 OUTSIDE Vermis 9 Vermis 9 | 55 26 12 2 | 95 | 0.048 |

*Significance threshold T(21) = 3.53; Cluster extent threshold = 95 voxels.*

**( InfOwnPos > InfOtherPos ) – negative association with Anx Hx**

| **Cluster** | **Regions** | **Number of voxels in region** | **Cluster size** | **FWE-corrected p-value** |
| --- | --- | --- | --- | --- |
| 1 | L Middle frontal gyrus L SFG, medial L SFG, dorsolateral R SFG, medial L Supplementary motor area R SFG, dorsolateral R Supplementary motor area | 268 106 73 52 31 17 5 | 552 | < 0.001 |
| 2 | R Middle frontal gyrus R SFG, dorsolateral OUTSIDE R IFG, opercular part | 244 43 5 2 | 294 | < 0.001 |
| 3 | L Paracentral lobule L Precentral gyrus L Postcentral gyrus L Precuneus OUTSIDE | 227 15 4 1 1 | 248 | < 0.001 |
| 4 | R IFG, orbital part R Middle frontal gyrus R IFG, triangular part R MFG, orbital part | 93 62 36 5 | 196 | 0.001 |
| 5 | R Precentral gyrus R SFG, dorsolateral R Postcentral gyrus | 127 32 17 | 176 | 0.002 |
| 6 | L Parahippocampal gyrus L Fusiform gyrus OUTSIDE L Hippocampus | 78 48 3 1 | 130 | 0.012 |
| 7 | L Middle occipital gyrus L Calcarine fissure and surrounding cortex L Inferior occipital gyrus L Superior occipital gyrus | 61 46  12 1 | 120 | 0.017 |
| 8 | L Inferior temporal gyrus L Middle temporal gyrus L Fusiform gyrus OUTSIDE L Hippocampus | 47 25 16 5 2 | 95 | 0.048 |

*Significance threshold T(21) = 3.53; Cluster extent threshold = 95 voxels.*

**( InfOwnPos > InfOtherPos ) – positive association with Model Intercept**

| **Cluster** | **Regions** | **Number of voxels in region** | **Cluster size** | **FWE-corrected p-value** |
| --- | --- | --- | --- | --- |
| 1 | L Caudate nucleus OUTSIDE L Olfactory cortex R Olfactory cortex R Anterior cingulate and paracingulate gyri L Anterior cingulate and paracingulate gyri | 42 36 24 16 11  3 | 132 | 0.011 |
| 2 | L Supplementary motor area R SFG, medial R Supplementary motor area OUTSIDE L SFG, medial | 77 34 11 2 1 | 125 | 0.014 |
| 3 | R Middle frontal gyrus R IFG, orbital part R IFG, triangular part R MFG, orbital part | 73 26 21 3 | 123 | 0.015 |

*Significance threshold T(21) = 3.53; Cluster extent threshold = 123 voxels.*

**[Section V.E.](#Table_of_Contents)** Regression against (InfOwnNeg > InfOtherNeg)

*------------------------------------------------------------------------------------------------------------------------------------------*

***[Model 36:](#Section_5_Table)*** $\left( InfOwnNeg > InfOtherNeg \right)= \beta_{0}+ \beta_{1}\left( CESD[T1\text{-}4] \right)$

*------------------------------------------------------------------------------------------------------------------------------------------*

**( InfOwnNeg > InfOtherNeg ) – positive association with CESD[T1-4]**

| **Cluster** | **Regions** | **Number of voxels in region** | **Cluster size** | **FWE-corrected p-value** |
| --- | --- | --- | --- | --- |
| 1 | R Crus Cerebellum 2 R Cerebellum 8 R Cerebellum 7b Vermis 8 | 99 3 2 1 | 105 | 0.038 |

*Significance threshold T(22) = 3.50; Cluster extent threshold = 105 voxels.*

**( InfOwnNeg > InfOtherNeg ) – negative association with CESD[T1-4]**

| **Cluster** | **Regions** | **Number of voxels in region** | **Cluster size** | **FWE-corrected p-value** |
| --- | --- | --- | --- | --- |
| 1 | L Insula L IFG, triangular part L IFG, orbital part OUTSIDE | 113 38 18 11 | 180 | 0.003 |

*Significance threshold T(22) = 3.50; Cluster extent threshold = 180 voxels.*

**( InfOwnNeg > InfOtherNeg ) – positive association with Model Intercept**

| **Cluster** | **Regions** | **Number of voxels in region** | **Cluster size** | **FWE-corrected p-value** |
| --- | --- | --- | --- | --- |
| 1 | R Middle temporal gyrus R Inferior temporal gyrus OUTSIDE R Middle occipital gyrus R Inferior occipital gyrus R Fusiform gyrus | 306 261 148 122 55 36 | 928 | < 0.001 |
| 2 | L Calcarine fissure and surrounding cortex L Lingual gyrus L Superior occipital gyrus L Cerebellum 6 L Middle occipital gyrus L Inferior occipital gyrus L Crus Cerebellum 1 OUTSIDE Vermis 6 L Cuneus | 136  96 48 42 40 8 7 7 4 3 | 391 | < 0.001 |
| 3 | L Middle occipital gyrus L Middle temporal gyrus OUTSIDE L Inferior occipital gyrus L Inferior temporal gyrus | 158 78 25 21 9 | 291 | < 0.001 |
| 4 | R Superior parietal gyrus R Precuneus OUTSIDE R IP, supramarginal and angular gyri R Paracentral lobule R Postcentral gyrus R Angular gyrus | 138 80 33 12  11 4 1 | 279 | < 0.001 |
| 5 | L Crus Cerebellum 1 L Cerebellum 8 L Cerebellum 7b OUTSIDE L Crus Cerebellum 2 | 67 29 11 9 2 | 118 | 0.023 |
| 6 | R Superior occipital gyrus R Cuneus R Calcarine fissure and surrounding cortex | 56 45 1 | 102 | 0.043 |
| 7 | OUTSIDE R SFG, orbital part R Caudate nucleus R MFG, orbital part R Anterior cingulate and paracingulate gyri R IFG, orbital part | 57 22 13 6 1  1 | 100 | 0.047 |

*Significance threshold T(22) = 3.50; Cluster extent threshold = 100 voxels.*

**( InfOwnNeg > InfOtherNeg ) – negative association with Model Intercept**

| **Cluster** | **Regions** | **Number of voxels in region** | **Cluster size** | **FWE-corrected p-value** |
| --- | --- | --- | --- | --- |
| 1 | R Superior temporal gyrus R Heschl gyrus R Temporal pole: STG R Middle temporal gyrus R Rolandic operculum R Insula OUTSIDE | 556 73 31 25 15 7 1 | 708 | < 0.001 |
| 2 | L Superior temporal gyrus L Middle temporal gyrus L Heschl gyrus L Rolandic operculum OUTSIDE | 445 100 41 12 1 | 599 | < 0.001 |

*Significance threshold T(22) = 3.50; Cluster extent threshold = 599 voxels.*

*------------------------------------------------------------------------------------------------------------------------------------------*

***[Model 37:](#Section_5_Table)*** $\left( InfOwnNeg > InfOtherNeg \right)= \beta_{0}+ \beta_{1}\left( BAI[T1\text{-}4] \right)$

*------------------------------------------------------------------------------------------------------------------------------------------*

**( InfOwnNeg > InfOtherNeg ) – positive association with Model Intercept**

| **Cluster** | **Regions** | **Number of voxels in region** | **Cluster size** | **FWE-corrected p-value** |
| --- | --- | --- | --- | --- |
| 1 | R Middle temporal gyrus R Inferior temporal gyrus OUTSIDE R Middle occipital gyrus R Inferior occipital gyrus R Fusiform gyrus R Crus Cerebellum 1 | 312 253 155 127 56 52 2 | 957 | < 0.001 |
| 2 | L Calcarine fissure and surrounding cortex L Lingual gyrus L Superior occipital gyrus L Cerebellum 6 L Middle occipital gyrus L Inferior occipital gyrus L Crus Cerebellum 1 OUTSIDE Vermis 6 L Fusiform gyrus L Cuneus | 125  88 48 45 34 8 7 6 5 2 2 | 370 | < 0.001 |
| 3 | L Middle occipital gyrus L Middle temporal gyrus OUTSIDE L Inferior occipital gyrus L Inferior temporal gyrus | 149 81 29 19 8 | 286 | < 0.001 |
| 4 | R Superior parietal gyrus R Precuneus OUTSIDE R Paracentral lobule R IP, supramarginal and angular gyri R Postcentral gyrus R Angular gyrus | 95 74 34 11 11  4 1 | 230 | 0.001 |
| 5 | L Crus Cerebellum 1 L Cerebellum 8 L Cerebellum 7b OUTSIDE L Crus Cerebellum 2 | 69 29 14 9 5 | 126 | 0.017 |
| 6 | R Superior occipital gyrus R Cuneus R Calcarine fissure and surrounding cortex | 71 48 1 | 120 | 0.022 |
| 7 | OUTSIDE R SFG, orbital part R Caudate nucleus R MFG, orbital part R IFG, orbital part R Anterior cingulate and paracingulate gyri | 60 24 13 8 2 1 | 108 | 0.035 |

*Significance threshold T(22) = 3.50; Cluster extent threshold = 108 voxels.*

**( InfOwnNeg > InfOtherNeg ) – negative association with Model Intercept**

| **Cluster** | **Regions** | **Number of voxels in region** | **Cluster size** | **FWE-corrected p-value** |
| --- | --- | --- | --- | --- |
| 1 | R Superior temporal gyrus R Heschl gyrus R Temporal pole: STG R Middle temporal gyrus R Rolandic operculum R Insula OUTSIDE | 557 73 33 23 14 4 1 | 705 | < 0.001 |
| 2 | L Superior temporal gyrus L Middle temporal gyrus L Heschl gyrus L Rolandic operculum OUTSIDE | 443 102 41 10 1 | 597 | < 0.001 |

*Significance threshold T(22) = 3.50; Cluster extent threshold = 597 voxels.*

*------------------------------------------------------------------------------------------------------------------------------------------*

***[Model 38:](#Section_5_Table)*** $\left( InfOwnNeg > InfOtherNeg \right)= \beta_{0}+ \beta_{1}\left( Dep Hx \right) + \beta_{2}\left( Dep Dx \right)$

*------------------------------------------------------------------------------------------------------------------------------------------*

**( InfOwnNeg > InfOtherNeg ) – positive association with Dep Hx**

| **Cluster** | **Regions** | **Number of voxels in region** | **Cluster size** | **FWE-corrected p-value** |
| --- | --- | --- | --- | --- |
| 1 | R SFG, dorsolateral R Middle frontal gyrus OUTSIDE | 46 44 21 | 111 | 0.026 |

*Significance threshold T(21) = 3.53; Cluster extent threshold = 111 voxels.*

**( InfOwnNeg > InfOtherNeg ) – positive association with Model Intercept**

| **Cluster** | **Regions** | **Number of voxels in region** | **Cluster size** | **FWE-corrected p-value** |
| --- | --- | --- | --- | --- |
| 1 | R Middle temporal gyrus R Inferior temporal gyrus OUTSIDE R Middle occipital gyrus R Fusiform gyrus R Inferior occipital gyrus | 158 150 36 18 7 4 | 373 | < 0.001 |
| 2 | L Lingual gyrus L Superior occipital gyrus L Calcarine fissure and surrounding cortex L Middle occipital gyrus L Cerebellum 6 L Cuneus Vermis 6 OUTSIDE L Inferior occipital gyrus L Fusiform gyrus | 73 55 53  39 34 12 8 6 3 1 | 284 | < 0.001 |

*Significance threshold T(21) = 3.53; Cluster extent threshold = 284 voxels.*

**( InfOwnNeg > InfOtherNeg ) – negative association with Model Intercept**

| **Cluster** | **Regions** | **Number of voxels in region** | **Cluster size** | **FWE-corrected p-value** |
| --- | --- | --- | --- | --- |
| 1 | L Superior temporal gyrus L Middle temporal gyrus L Heschl gyrus L Rolandic operculum | 190 29 22 4 | 245 | < 0.001 |
| 2 | R Superior temporal gyrus R Middle temporal gyrus | 122 3 | 125 | 0.015 |
| 3 | R Superior temporal gyrus R Heschl gyrus R Temporal pole: STG R Insula R Rolandic operculum OUTSIDE | 83 24 8 7 1 1 | 124 | 0.015 |

*Significance threshold T(21) = 3.53; Cluster extent threshold = 124 voxels.*

*------------------------------------------------------------------------------------------------------------------------------------------*

***[Model 39:](#Section_5_Table)*** $\left( InfOwnNeg > InfOtherNeg \right)= \beta_{0}+ \beta_{1}\left( Anx Hx \right)+ \beta_{2}\left( Anx Dx \right)$

*------------------------------------------------------------------------------------------------------------------------------------------*

**( InfOwnNeg > InfOtherNeg ) – positive association with Model Intercept**

| **Cluster** | **Regions** | **Number of voxels in region** | **Cluster size** | **FWE-corrected p-value** |
| --- | --- | --- | --- | --- |
| 1 | R Inferior temporal gyrus R Middle temporal gyrus OUTSIDE R Middle occipital gyrus R Inferior occipital gyrus R Fusiform gyrus | 235 187 89 65 30 16 | 622 | < 0.001 |
| 2 | L Lingual gyrus L Calcarine fissure and surrounding cortex L Middle occipital gyrus L Superior occipital gyrus L Fusiform gyrus L Inferior occipital gyrus OUTSIDE | 72 34  25 15 9 8 7 | 170 | 0.003 |
| 3 | R Fusiform gyrus OUTSIDE R Cerebellum 6 R Cerebellum 4-5 R Inferior temporal gyrus | 89 24 12 9 9 | 143 | 0.008 |

*Significance threshold T(21) = 3.53; Cluster extent threshold = 143 voxels.*

**( InfOwnNeg > InfOtherNeg ) – negative association with Model Intercept**

| **Cluster** | **Regions** | **Number of voxels in region** | **Cluster size** | **FWE-corrected p-value** |
| --- | --- | --- | --- | --- |
| 1 | L Superior temporal gyrus L Middle temporal gyrus L Heschl gyrus L Rolandic operculum | 208 51 12 1 | 272 | < 0.001 |
| 2 | R Superior temporal gyrus R Middle temporal gyrus R Heschl gyrus | 181 18 3 | 202 | 0.001 |

*Significance threshold T(21) = 3.53; Cluster extent threshold = 202 voxels.*

**[Section V.F.](#Table_of_Contents)** [Additional Exploratory Models](#Table_of_Contents)

Additional models were used to explore if variation in future depression and anxiety symptoms were better accounted for by difference in neural response to a mother’s own infant or exposure to lifetime or parenting-related stress. The Life Experiences Survey (LE)^[7]^ and the Parenting Stress Scale (PS)^[8]^ collected at all 4 time points were averaged to produce a measure of general life and parenting stress (*LE_T1-4_, PS_T1-4_)*. It was found that only *LE_T1-4_* was significantly correlated with future anxiety (BAI at Time 4) symptoms and neither measure was linearly correlated with future depression (CESD at Time 4). Subject-level mean contrast values for *InfOwnPos > InfOwnNeg* were then extracted from voxels that had already displayed significant negative association with depression or anxiety symptoms going forward (*i.e.* from the clusters of negative association with either *BAI Time1-4 Intercept* or *CESD Time1-4 Intercept* reported in the main manuscript).

The predictors (*LE_T1-4_, PS_T1-4_, (InfOwnPos > InfOwnNeg)_BAI_,* and *(InfOwnPos > InfOwnNeg)_CESD_)* were regressed against time 4 BAI or CESD scores in the following full models:

$\boldsymbol{BAI}_{\boldsymbol{T}\boldsymbol{4}}\boldsymbol{=}\boldsymbol{\beta}_{\boldsymbol{0}}\boldsymbol{+}\boldsymbol{\beta}_{\boldsymbol{1}}\left( \boldsymbol{InfOwnPos>InfOwnNeg} \right)_{\boldsymbol{BAI}}\boldsymbol{+}\boldsymbol{\beta}_{\boldsymbol{2}}\boldsymbol{LE}_{\boldsymbol{T}\boldsymbol{1-4}}\boldsymbol{+}\boldsymbol{\beta}_{\boldsymbol{3}}\boldsymbol{PS}_{\boldsymbol{T}\boldsymbol{1-4}}$ **(1)**

$\boldsymbol{CESD}_{\boldsymbol{T}\boldsymbol{4}}\boldsymbol{=}\boldsymbol{\beta}_{\boldsymbol{0}}\boldsymbol{+}\boldsymbol{\beta}_{\boldsymbol{1}}\left( \boldsymbol{InfOwnPos>InfOwnNeg} \right)_{\boldsymbol{CESD}}\boldsymbol{+}\boldsymbol{\beta}_{\boldsymbol{2}}\boldsymbol{LE}_{\boldsymbol{T}\boldsymbol{1-4}}\boldsymbol{+}\boldsymbol{\beta}_{\boldsymbol{3}}\boldsymbol{PS}_{\boldsymbol{T}\boldsymbol{1-4}}$ **(2)**

The cluster mean signal values significantly predicted both time 4 symptom scores with no meaningful reduction in coefficients when stress measures across time points are added to the models. It seems even though the stress scales relate to symptom outcomes at the zero-order correlation level, they do not contribute as significant predictors when added to a model including the neural signal predictor.

**References**

[1] Tzourio-Mazoyer, N., Landeau, B., Papathanassiou, D., Crivello, F., Etard, O., Delcroix, N., ... & Joliot, M. (2002). Automated anatomical labeling of activations in SPM using a macroscopic anatomical parcellation of the MNI MRI single-subject brain. *Neuroimage*, 15(1), 273-289.

[2] Radloff LS (1977): The CES-D scale: A self-report depression scale for research in the general population. Applied Psychological Measurement 1(3): 385–401.

[3] Beck AT, Epstein N, Brown G, Steer RA (1988): An inventory for measuring clini-cal anxiety: Psychometric properties. J Consult Clin Psychol 56(6): 893–897.

[4] First MB, Spitzer RL, Gibbon M, Williams JBW (1994): *Structured clinical inter-view for Axis I DSM-IV disorders*. New York: Biometrics Research.

[5] Cox, J. L., Holden, J. M., & Sagovsky, R. (1987). Detection of postnatal depression: development of the 10-item Edinburgh Postnatal Depression Scale. *The British journal of psychiatry*, 150(6), 782-786.

[6] Laurent, H. (2017). Early calibration of the HPA axis by maternal psychopathology. *Psychoneuroendocrinology*, 78, 177-184.

[7] Sarason, I. G., Johnson, J. H., & Siegel, J. M. (1978). Assessing the impact of life changes: development of the Life Experiences Survey. *Journal of consulting and clinical psychology*, 46(5), 932.

[8] Berry, J. O., & Jones, W. H. (1995). The parental stress scale: Initial psychometric evidence. *Journal of Social and Personal Relationships*, 12(3), 463-472.
